# RRP15 deficiency induces ribosome stress to inhibit colorectal cancer proliferation and metastasis via LZTS2-mediated β-catenin suppression

**DOI:** 10.1038/s41419-023-05578-6

**Published:** 2023-02-07

**Authors:** Zhixiong Dong, Jinhai Li, Wenqing Dai, Dongbo Yu, Youjuan Zhao, Shuanghui Liu, Xuanwen Li, Zhengzheng Zhang, Rui Zhang, Xue Liang, Qingran Kong, Shengnan Jin, Hao Jiang, Wei Jiang, Chunming Ding

**Affiliations:** 1 Zhejiang Provincial Key Laboratory of Medical Genetics, Key Laboratory of Laboratory Medicine, Ministry of Education, China, School of Laboratory Medicine and Life Science, Wenzhou Medical University, Wenzhou, Zhejiang 325000 P. R. China; 2 Department of Liver and Gall Surgery, the Third Affiliated Hospital of Wenzhou Medical University, Zhejiang, 325200 P. R. China; 3 Central Laboratory of the First Affiliated Hospital, Weifang Medical University, Weifang, Shandong 261000 P. R. China; 4 State Key Laboratory of Molecular Oncology, Cancer Institute and Hospital, Chinese Academy of Medical Sciences and Peking Union Medical College, Beijing, 100021 P. R. China; 5 Department of Biomedical Informatics, School of Life Sciences, Central South University, Changsha, Hunan 410013 P. R. China

**Keywords:** Tumour biomarkers, Colorectal cancer, Colorectal cancer

## Abstract

Ribosome biogenesis (RiBi) plays a pivotal role in carcinogenesis by regulating protein translation and stress response. Here, we find that RRP15, a nucleolar protein critical for RiBi and checkpoint control, is frequently upregulated in primary CRCs and higher RRP15 expression positively correlated with TNM stage (*P* < 0.0001) and poor survival of CRC patients (*P* = 0.0011). Functionally, silencing RRP15 induces ribosome stress, cell cycle arrest, and apoptosis, resulting in suppression of cell proliferation and metastasis. Overexpression of RRP15 promotes cell proliferation and metastasis. Mechanistically, ribosome stress induced by RRP15 deficiency facilitates translation of TOP mRNA *LZTS2* (Leucine zipper tumor suppressor 2), leading to the nuclear export and degradation of β-catenin to suppress Wnt/β-catenin signaling in CRC. In conclusion, ribosome stress induced by RRP15 deficiency inhibits CRC cell proliferation and metastasis via suppressing the Wnt/β-catenin pathway, suggesting a potential new target in high-RiBi CRC patients.

## Introduction

Colorectal cancer (CRC) is the third most commonly diagnosed malignant tumors worldwide [1]. Up to now, the prognosis of CRC particularly advanced CRC remains unsatisfactory due to its postoperative recurrence and metastasis, and the 5-year overall survival rate of CRC patients is still less than 60% [2]. Therefore, it is critical to search effective biomarkers and/or targets, explore their role in CRC progression, and investigate the prognostic and therapeutic values in clinic.

The accumulation of diverse genetic mutations and epigenetic variation activates several oncogenic signaling critical for the pathogenesis of CRC [3]. Among them, abnormal activation of Wnt signaling is commonly observed in CRC carcinogenesis. The main reason of Wnt activation is loss-of-functional mutation of an important tumor suppressor *adenomatous polyposis coli* (*APC*). After *APC* is mutated and inactivated, which can akin to Wnt ligand stimulation, β-catenin is released from destruction complex and subsequently translocated into nucleus and interacts with TCF (T-cell factor)/LEF (Lymphoid enhancer binding factor), which controls multiple genes expression involving in cell proliferation, metastasis and chemotherapeutic drugs resistance, such as *cyclin D1*, *c-Myc*, *MMP-9*, *AXIN2*, etc [4]. In addition, β-catenin subcellular localization is also fine-tuned by other approaches. For instance, it has been reported that several proteins including forkhead box protein M1 (FoxM1), B-cell lymphoma 9 (BCL9) fascinate β-catenin importing into the nucleus whereas other proteins, such as LZTS2, APC, AXIN, and Ran binding protein 3 (RanBP3) promote β-catenin exporting from nucleus [5–10]. Therefore, interruptions of various steps involving β-catenin nuclear-cytoplasmic shuttling have potential to improve anticancer treatment.

RiBi is a multistep biological progress that initiates in the nucleolus and terminates in the formation of functional ribosomes in the cytoplasm, and multiple regulators associated with pre-rRNA transcription, rRNA processing, ribosome assembly, and transport are involved in the progress. To meet the demand of aberrant growth and proliferation, cancer cells require increased protein synthesis and overactive translation that depends on hyperactive RiBi. Therefore, larger size and increased number of nucleoli have been recognized as hallmarks of many types of malignant neoplasm [11]. The feature renders cancer cells a potentially exploitable vulnerability that can be utilized to kill cancer cells in clinical tumor therapy. For example, CX-5461, a first-in-human specific RNA polymerase I (Pol I) inhibitor, has been utilized in phase I clinical trials for therapy of advanced hematological cancers and solid tumors [12, 13].

RRP15, a ribosome RNA processing protein presented in the nucleolus, plays critical roles in RiBi via controlling pre-rRNA transcription, rRNA processing, and pre-ribosome subunits assembly [14–16]. Meanwhile, Wu et al. and us showed that RRP15 participates in regulating cell proliferation, cell cycle progression, and apoptosis [16, 17]. We found that perturbation of RRP15-dependent RiBi in p53 proficient non-transformed cells activated the classical impaired ribosome biogenesis checkpoint (IRBC) and induced cell cycle G1-G1/S arrest, while depletion of RRP15 in p53-deficient tumor cells induced S-G2/M arrest and apoptosis via activating the ATR-Chk1-γH2AX axis and DNA replication/damage checkpoint responses [16]. These selective checkpoint responses, cell cycle arrest, and/or cytotoxicity induced by RRP15-dependent ribosome stress in cells with different p53 function indicated that RRP15 might be a prospective target for cancer therapy. Although RRP15 was reported to overexpress in hepatocellular carcinoma (HCC), and knockdown of RRP15 in HCC cells induced senescence or apoptosis depending on p53 status [18], the precise molecular mechanism(s) by which RRP15 is involved in human carcinogenesis remains unclear.

Therefore, we explore the role of RRP15 in regulating CRC progression and reveal that RRP15 is frequently upregulated in human CRC, and its high expression is positively associated with TNM stage and worse prognosis of CRC patients. Accordingly, we demonstrate that RRP15 expression is positively related to the proliferative and metastatic ability of CRC cells in vitro and in vivo. RRP15 knockdown induced ribosome stress, reprogram protein translation profile to enhance the translation of TOP mRNA *LZTS2*, leading to nuclear export and degradation of β-catenin. These findings support that RRP15 may serve as a predictive marker and target with therapeutic potential in CRC.

## Results

### RRP15 is upregulated and predicts poor survival in CRC patients

To assess the clinical significance of RRP15 in CRC progression, we analyzed *RRP15* mRNA expression in CRC tissues using the online database (http://ualcan.path.uab.edu/index.html), and found that the mRNA level of *RRP15* in CRC tissues were significantly higher than that in normal tissues (Fig. S1A, B). In addition, data from *The Cancer Genome Atlas* (TCGA) showed that *RRP15* mRNA expression level is closely related to disease-free survival (DFS) (*P* = 0.0016) and progression-free survival (PFS) (*P* = 0.004) of CRC patients (Fig. S1C, D).

**Fig. 1:**
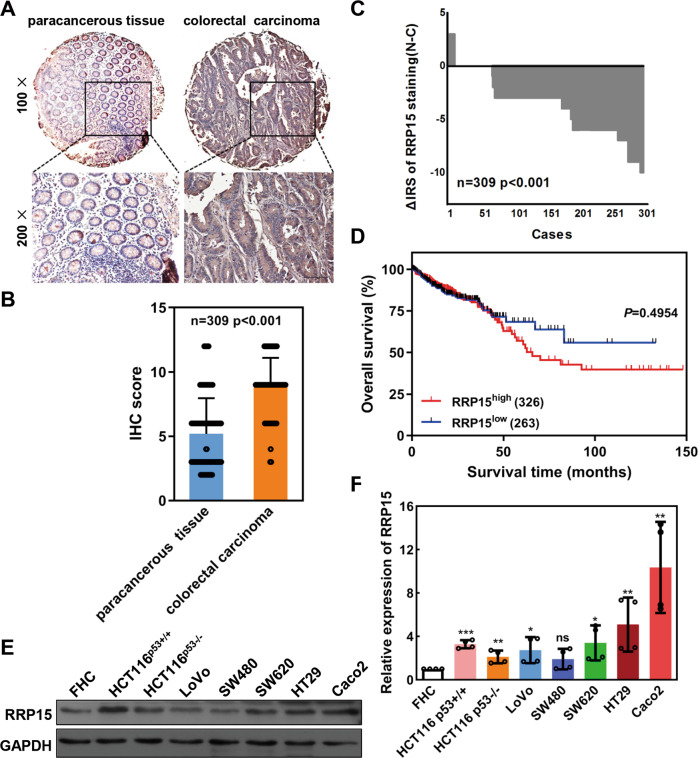
RRP15 is frequently upregulated in CRC and associated with prognosis of CRC patients. **A** Representative immunohistochemical staining images of RRP15 in CRC TMAs. Note: top panel, magnification ×100; bottom panel, magnification ×200. Scale bar: 100 μm. **B** The IHC score was calculated to qualify the immunostaining intensities of RRP15 in (**A**). **C** The distribution of the difference in staining intensities of RRP15 in each colorectal carcinoma tissue compared with paired adjacent non-cancerous tissue. N, paired adjacent non-cancerous tissues; C, colorectal carcinoma tissues. **D** Kaplan–Meier survival curve of CRC patients with high or low RRP15 expression. Curve comparison was performed with log-rank (Mantel–Cox) test. (**E**) Western blot analysis of RRP15 expression in different CRC cell lines compared to a normal colon epithelial cell line (FHC). **F** Quantification of the relative protein level of RRP15 in (**E**). Values are means ± SD (standard deviation) derived from four independent experiments. ns: no significant; *P < 0.05, **P < 0.01 and ***P < 0.001 compared to FHC cell.

Consistent with TCGA data, the expression of *RRP15* mRNA is frequently increased in tumor tissues as compared with the corresponding paracarcinoma tissues from 20 CRC patients (Fig. S1E, F). To further analyze the RRP15 protein expression, we chose a cohort of 309 CRC cases with tissue microarrays and survival data (Table 1). RRP15 protein expression examined by IHC was significantly upregulated in cancer tissues as compared with surrounding normal tissues (*P* < 0.001, Fig. 1A, B), with 234 cases showing upregulation of RRP15 in cancer tissue (Fig. 1C). The upregulation of RRP15 was significantly correlated with TNM stage (*P* < 0.0001, Table 1). Patients with upregulated RRP15 had worse overall survival (OS) (*P* = 0.0011, Fig. 1D). These results collectively reveal that RRP15 expression is elevated in CRC and correlates with unfavorable clinicopathological characteristics and poor prognosis, which hints that RRP15 may contribute to CRC development and serve as a potential prognostic marker.

**Table 1:** Relationship between RRP15 expression and clinicopathological features of CRC patients.

Variables		RRP15 expression (*n* = 309 cases)		*P*
		Low (%)	High (%)	
All patients	309 (100)	75 (24.3)	234 (75.7)	
Age (years)				0.789
	≤60	33 (44.0)	98 (41.9)	
	>60	42 (56.0)	136 (58.1)	
Gender				0.418
	Males	41 (54.7)	142 (60.7)	
	Females	34 (45.3)	92 (39.3)	
TNM stage				
	I	22 (29.3)	15 (6.4)	<0.0001
	II	29 (38.7)	129 (55.1)	
	III	21 (28.0)	82 (35.0)	
	IV	3 (4.0)	8 (3.4)	
Tumor diameter				0.109
	≤5 cm	59 (78.7)	161 (68.8)	
	>5 cm	16 (21.3)	73 (31.2)	
Lymph node metastasis				1
	N0	49 (65.3)	154 (65.8)	
	N1/N2/N3	26 (34.7)	80 (34.2)	
Distant metastasis				1
	M0	72 (96.0)	225 (96.2)	
	M1	3 (4.0)	9 (3.8)

### RRP15 regulates cell proliferation, cell cycle progression, and apoptosis of CRC cells in vitro

To investigate the role of RRP15 in CRC development, we examined RRP15 expression in a normal colorectal epithelial cell (FHC) and seven human-derived CRC cell lines by western blot and qRT-PCR. The results showed that most CRC cells expressed higher RRP15 at both protein and mRNA levels compared with FHC cells, except for the SW480 cell line, a cell line with low metastatic potential, whose RRP15 expression was comparable to FHC cells (Figs. 1E, F and S1G). Consistent with the critical role of RRP15 in nucleolar formation and RiBi, immunostaining results showed that CRC cells displayed larger size and increased number of nucleoli when compared to FHC cells (Fig. S2). These results suggest that CRC cells upregulate RRP15 to enhance RiBi and meet the requirements of cell proliferation.

**Fig. 2:**
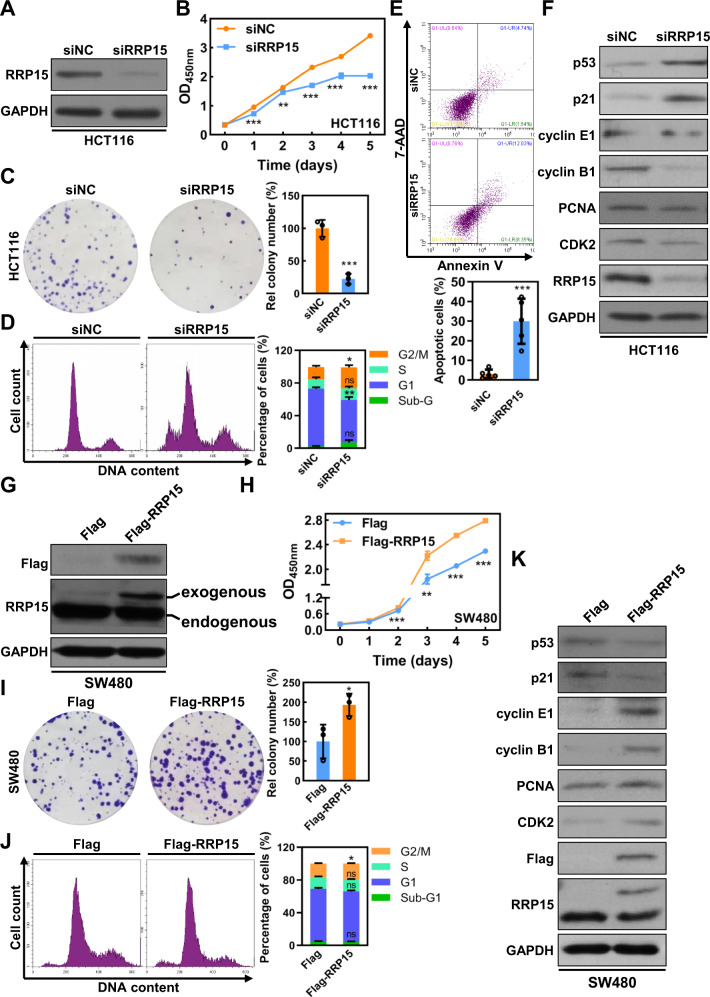
RRP15 positively regulates proliferation of CRC cells. **A** Western blot analysis of RRP15 expression in HCT116 cells transfected with siNC or siRRP15. **B** Proliferation curves of HCT116 cells after transfected with siNC or siRRP15 or indicated time. **C** Colony formation assay to detect the cell proliferation ability of HCT116 cells after RRP15 KD. The colony numbers were counted using Image J software. Cell cycle profile (**D**) and apoptosis (**E**) of HCT116 cells after transfected with siNC or siRRP15. **F** Western blot analysis of the cell proliferative and cell cycle-related proteins of HCT116 cells after indicated siRNA transfection. **G** Western blot analysis of RRP15 expression in SW480 cells transduced with pCDH-RRP15 or vector. Proliferation ability of SW480 cells with stably RRP15 OE and controls were examined using CCK8 (**H**) and colony formation (**I**) assay. **J** Cell cycle profile of SW480 cells with stably RRP15 OE and controls. **K** Western blot analysis of the cell proliferative and cell cycle-related proteins of SW480 cells with stably RRP15 OE and controls. The experiments were repeated at least three times independently. Data are shown as mean ± standard deviations. *P < 0.05, **P < 0.01, and ***P < 0.001.

RRP15 knockdown (KD) was performed in HCT116 using a siRNA targeting the RRP15 3′UTR (Fig. 2A). As previously reported in HeLa cells, RRP15 KD led to ribosome stress with dispersed localization of nucleolar markers and decreased 47S rRNA level in HCT116 cells (Fig. S3) [16]. Proliferative assay results showed that RRP15 KD significantly inhibited the cell proliferation and colony formation (Fig. 2B, C). We also established a RRP15-knockdown HCT116 cell line (shRRP15) (Fig. S4A), and demonstrated that the proliferative ability of HCT116-shRRP15 cells was significantly impaired compared with controls (Fig. S4B). Meanwhile, to eliminate the potential off-target of siRNA, a second siRNA targeting the RRP15 CDS region (siRRP15-CDS) was designed. As shown in Fig. S4C, D, siRRP15-CDS also significantly inhibited RRP15 expression and proliferation of HCT116 cells. Moreover, FACS and Annexin V/7-AAD staining assays showed that RRP15 KD caused cell cycle arrest at G2/M phase and apoptosis in HCT116 cells (Fig. 2D, E). Consistently, RRP15 KD caused an increased expression of p53, p21, cleaved caspase 9, and cleaved caspase 7, and resulted in a decreased expression of PCNA, cyclin E, cyclin B1 and CDK2 in HCT116 cells with wild-type p53 (Figs. 2F and S4E). Similar phenomena were observed in SW620 cells (another RRP15 high-expressed cell with mutated p53) when RRP15 KD (Fig. S4F–K). In general, we demonstrate that RRP15 KD causes ribosome stress, thereby inhibits cell proliferation via inducing cell cycle arrest and apoptosis in CRC cells.

In addition, we constructed a RRP15-overexpressed SW480 cell line with lentivirus. The overexpression of RRP15 was confirmed by western blot (Fig. 2G). Consistent with previous report [16], 47S rRNA level was significantly increased in RRP15 OE cells (Fig. S4L). Compared with control cells, RRP15 OE dramatically enhanced cell proliferation and accelerated cell cycle progression (Fig. 2H–J), and was accompanied by a distinct increase of PCNA, cyclin E, cyclin B1, and CDK2 but a decrease expression of p53 and p21 (Fig. 2K). Nevertheless, RRP15 OE did not affect cell apoptosis in SW480 cells (Fig. S4M). However, RRP15 OE only weakly increased the proliferative ability in HCT116 cells with higher RRP15 expression (Fig. S4N–O).

Our previous study revealed that RRP15 depletion specifically induced apoptosis in cancer cells (HeLa and MCF7) but not in an immortalized diploid cell line (hTERT-RPE1) [16]. Therefore, to test whether RRP15 KD exclusively induced apoptosis in CRC cells, we inhibited RRP15 expression in FHC cells, and found that RRP15 KD also significantly inhibited cells proliferation of FHC cells, but did not cause a significant increase in apoptotic cells (Fig. S5). Collectively, these results indicate that RRP15 expression in CRC cells is closely correlated with cell proliferation and cell cycle progression, and RRP15 may be a potential target to induce cancer-specific apoptosis in CRC cells.

### RRP15 facilitates migration and invasion of CRC cells in vitro

**Fig. 3:**
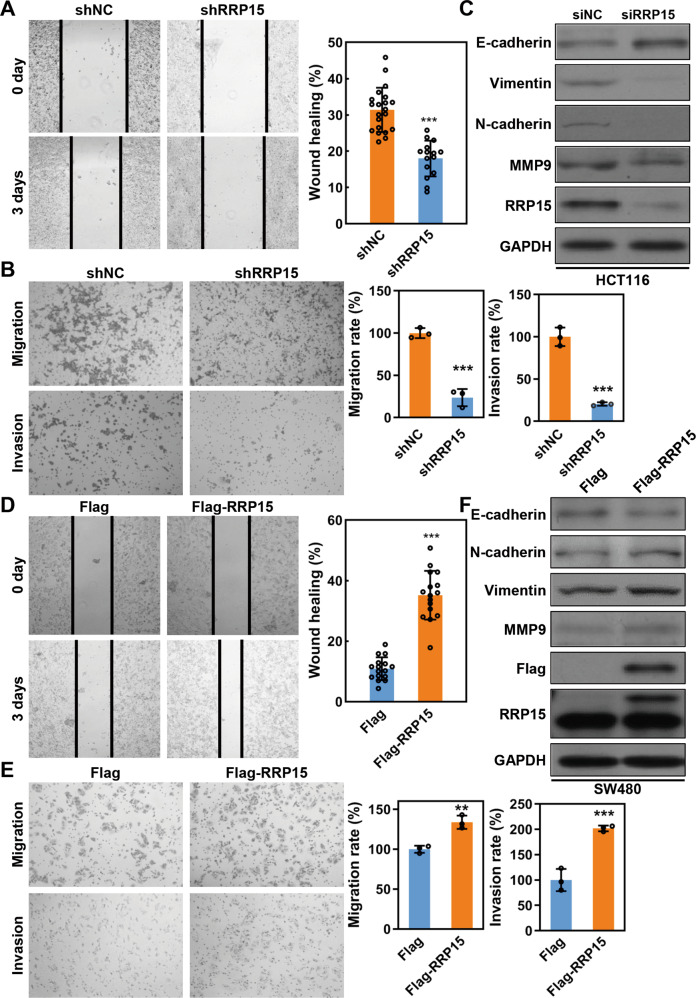
RRP15 positively regulates migration and invasion of CRC cells. Wound healing assay (**A**), transwell migration and invasion assay (**B**) were used to assess the migratory and invasive ability of HCT116 cells with stably shRRP15 KD or controls. **C** Western blot for EMT-related proteins levels in HCT116 cells transfected with siNC or siRRP15. Wound healing assay (**D**), transwell migration and invasion assay (**E**) of SW480 cells with stably RRP15 OE and controls. **F** Western blot for EMT-related proteins levels in SW480 cells after RRP15 OE. All experiments were performed at least three times. Data are shown as mean ± standard deviations. **P < 0.01 and ***P < 0.001.

We next investigated the effects of RRP15 expression on the metastatic potential of CRC cells. Wound healing assay showed that RRP15 KD significantly delayed the cell motility to the wound area in HCT116 cells (Fig. 3A). Transwell assay further revealed that RRP15 KD dramatically decreased the migration and invasion ability of HCT116 cells (Fig. 3B). We also examined the expression of EMT-related factors in RRP15 KD CRC cells. Compared with control cells, a decreased expression of mesenchymal markers (Vimentin, N-cadherin and MMP9) and an increased expression of epithelial marker E-cadherin were observed in RRP15 KD cells (Figs. 3C and S6A). Meanwhile, RRP15 OE enhanced the migration and invasion capacity of CRC cells, increased the expression of mesenchymal markers, and decreased the expression of E-cadherin (Figs. 3D–F and S6B, C). Therefore, our results indicate that RRP15 could expedite the metastasis of CRC cells, at least in part, via enhancing EMT.

### RRP15 promotes CRC tumor growth and metastasis in vivo

**Fig. 4:**
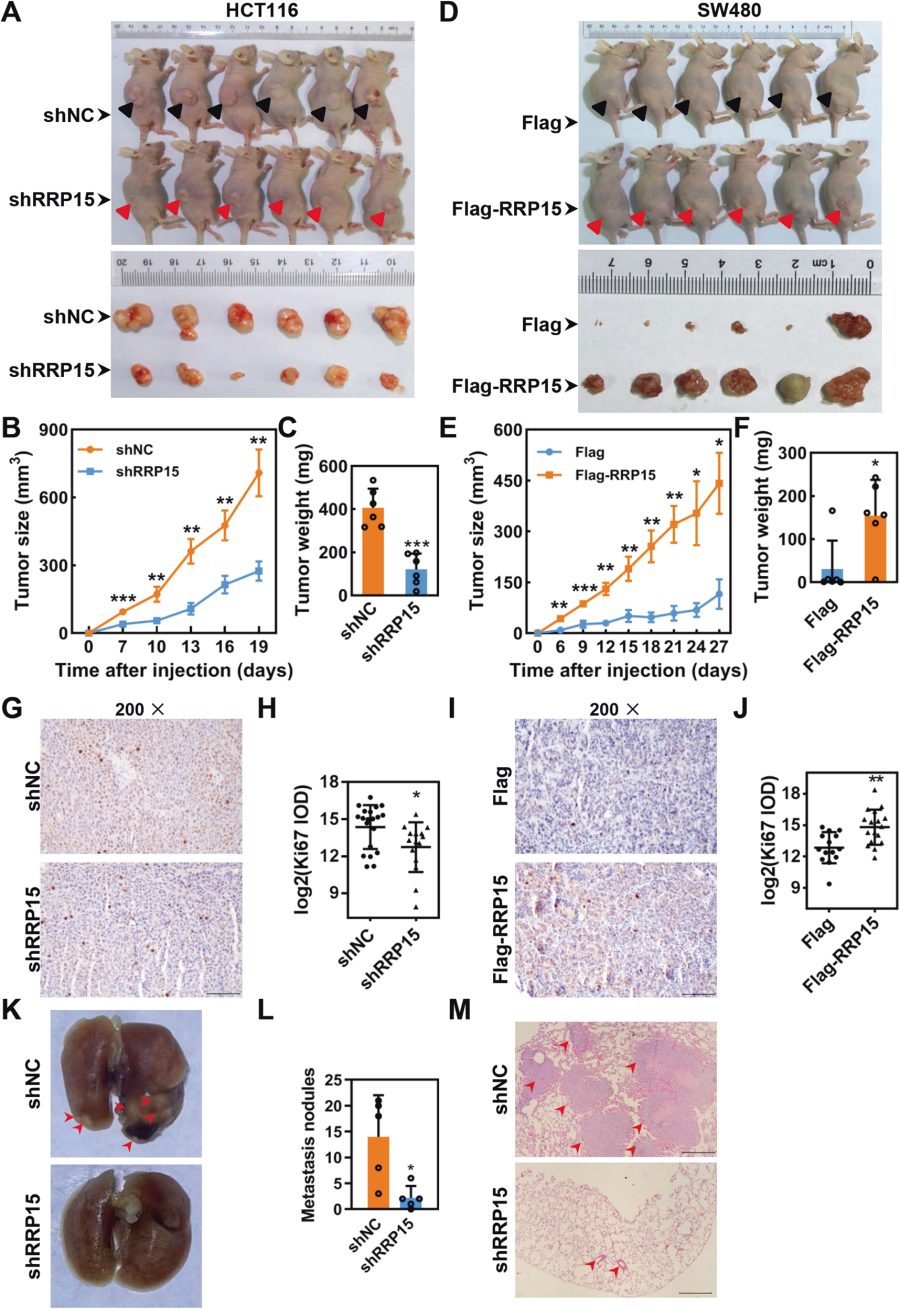
RRP15 positively regulates CRC progression in vivo. **A** Image of subcutaneous xenograft tumor formed by HCT116 cells with stably shRRP15 KD or controls in nude mice (*n* = 6). Tumor growth curve (**B**) and statistical analysis of the weight of tumors (**C**) in (**A**) (*n* = 6). **D** Image of subcutaneous xenograft tumor formed by SW480 cells with stably RRP15 OE and controls. Tumor growth curve (**E**) and statistical analysis of the weight of tumors (**F**) in (**D**) (*n* = 6) xenograft tumor growth and increases tumor weights. **G**–**J** Representative images of Ki67 staining in tissues from indicated xenograft tumor. The intensities of each image were analyzed by IOD through Image-Pro Plus 6.0 software. **K**, **L** Images of lung sections from mice injected via the tail vein with HCT116 cells with stable RRP15 KD or controls on Day 42 postinjection (*n* = 5). Red arrows indicate metastatic nodules on the lung surface and the tumor number is counted. **M** Representative images of H&E staining in lung sections from (**K**), and red arrows indicate metastatic nodules. Scale bar: 100 μm. Data are shown as mean ± standard deviations. *P < 0.05, **P < 0.01, and ***P < 0.001.

To further assess the effect of RRP15 on CRC tumor growth in vivo, HCT116-shRRP15, SW480-Flag-RRP15, and their corresponding control cells were injected subcutaneously into nude mice. The results showed that RRP15 KD significantly retarded the tumor growth (Fig. 4A–C), whereas ectopic RRP15 obviously accelerated the tumor growth (Fig. 4D–F). Moreover, Ki67 staining showed that, compared with corresponding controls, lower IOD of Ki67 was observed in shRRP15 tumors, whereas Ki-67 intensity in RRP15 OE tumors was markedly higher (Fig. 4G–J).

To verify the effect of RRP15 on CRC metastasis in vivo, a lung metastasis model was established by tail vein injection of HCT116-shRRP15. As shown in Fig. 4K, L, RRP15 KD significantly decreased the number of pulmonary metastatic nodules in mice when compared with controls. In addition, H&E-staining also displayed consistent results in mice lung tissues (Fig. 4M). Altogether, these data suggest that inhibition of RRP15 expression impairs CRC growth and metastasis in vivo.

### RRP15 regulates Wnt/β-catenin signaling pathway in CRC cells via regulating the subcellular localization of β-catenin

**Fig. 5:**
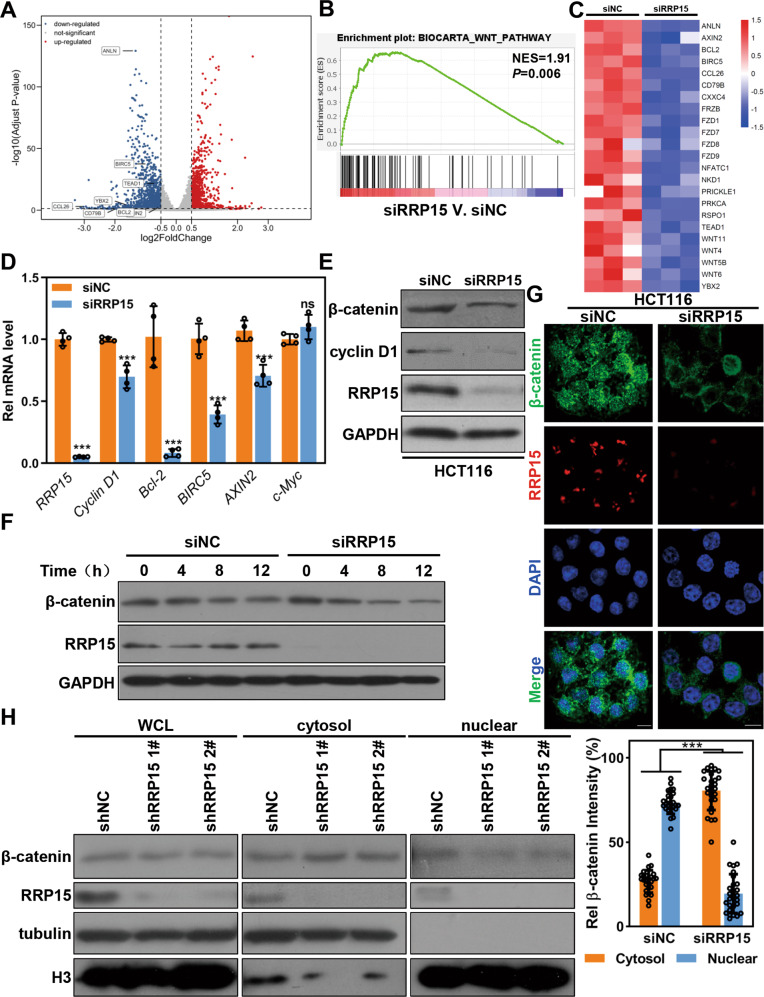
RRP15 positively regulates Wnt/β-catenin signaling pathway in CRC cells. **A** A volcano plot comparing the genes expression between siNC and siRRP15 transfected HCT116 cells. Plotted for each transcript is the negative log10 of the *P* value and the log2 of the fold change (FC) of gene expression of siRRP15 versus siNC transfected cells. The vertical dotted-lines represent log_2_FC are ±0.5, and the horizontal dotted-line represents an FDR threshold of 5%. **B** GSEA analysis of the relationship between RRP15 expression with Wnt pathway (NES = 1.91, *P* < 0.006, FDR = 0.05). **C** Heatmap of differentially expressed Wnt/β-catenin signaling pathway genes in siRRP15 or siNC transfected HCT116 cells. **D** qRT-PCR analysis of indicated Wnt/β-catenin targets in siRRP15 or siNC transfected HCT116 cells. **E** Western blot for β-catenin and cyclin D1 expression in siRRP15 or siNC transfected HCT116 cells. **F** β-catenin degradation analysis in siRRP15 or siNC transfected HCT116 cells after indicated treatment. **G** Representative images of immunostaining with indicated antibodies in siRRP15 or siNC transfected HCT116 cells. DNA was labeled with DAPI. Scale bar, 10 μm. The experiments were performed in triplicate. Relative β-catenin staining intensity in nucleus and cytosol were analyzed and data were obtained from at least 20 cells. **H** Nuclear or cytoplasmic lysates from HCT116 cells with stably RRP15 KD or controls were extracted and subjected to western blot analysis with indicated antibodies. The experiments were performed at least three times, and data are shown as mean ± standard deviations. ***P < 0.001, ns: not significant.

IRBC triggers p53-dependent cell cycle checkpoint activation and cell cycle arrest [19–21]. We thus first investigated the role of p53 in RRP15-mediated CRC inhibition. Co-deletion of p53 only partially reversed the proliferative inhibition and apoptosis and did not affect migration induced by RRP15 KD in HCT116 cells (Fig. S7). Considering similar phenotypes caused by RRP15 KD in SW620 cells with mutant p53, we speculated that there may be other novel p53-independent IRBC pathway(s) to mediate RRP15-induced CRC inhibition. To explore the mechanism by which RRP15 regulates CRC progression, we profiled the transcriptome of siRRP15 transfected HCT116 cells (Fig. S8A). We found 1473 transcripts downregulated and 1049 transcripts upregulated in siRRP15 compared to siNC (Log_2_FC < −0.5 or >0.5, and false discovery rate <0.05) (Fig. 5A). Gene set enrichment analysis (GSEA) and heatmap revealed a significant enrichment in Wnt/β-catenin pathway (Fig. 5B, C). We further performed qRT-PCR and verified that RRP15 KD significantly reduced the mRNA levels of genes directly regulated by β-catenin such as *cyclin D1*, *Bcl-2*, *BIRC5*, and *AXIN2* in CRC cells (Figs. 5D and S8B, C).

Consistently, RRP15 KD significantly reduced the expression of β-catenin protein and its target gene cyclin D1 in CRC cells (Figs. 5E and S8D). However, RRP15 KD did not cause a significant decrease in *β-catenin* mRNA (Fig. S8E), which implied that RRP15 may regulate β-catenin expression at the post-transcriptional level. RRP15 KD was reported to perturb nucleolar formation and induce RiBi impairment of both large and small subunits [16], and we have observed that RRP15 KD triggered severe RiBi deficiency with nucleolar proteins dispersing into nucleoplasm and a significant decrease of 47S rRNA (Fig. S3). Therefore, RRP15 KD may cause a decrease of β-catenin translation and/or an increase of β-catenin degradation. To differentiate these possibilities, we detected the expression of β-catenin in siNC and siRRP15 transfected cells after treatment with proteasome inhibitor MG132. Western blot results showed that RRP15 KD induced β-catenin reduction by accelerating its protein degradation (Fig. S8F).

Therefore, we further examined the stability of β-catenin in CRC cells after RRP15 KD. The results showed that RRP15 KD resulted in a significant decrease of β-catenin stability (Fig. 5F). Given β-catenin is regulated by dynamic nuclear trafficking, and cytoplasmic β-catenin could be degraded by ubiquitination-mediated proteasome system, we detected the effects of RRP15 on subcellular localization of β-catenin and found that β-catenin was enriched in cytoplasm when RRP15 KD in HCT116 cells (Fig. 5G). Subcellular nuclear and cytoplasmic fractions further verified that RRP15 KD caused an increase of β-catenin in cytosol but diminished in nucleus in CRC cells (Fig. 5H). Consistently, RRP15 OE increased β-catenin and cyclin D1 expression, and dramatically promoted the nuclear accumulation of β-catenin in SW480 cells (Fig. S8G–J).

In addition, to investigate the relationship between RRP15-dependent RiBi and Wnt/β-catenin signaling pathway, we utilized actinomycin D (actD), a compound that can specifically inhibit RNA polymerase I activity at low dose thereby trigger severe IRBC [22], to treat HCT116 cells, and found that actD treatment triggered dispersed localization of nucleolin and UBF (Fig. S9A, B), diminished expression of β-catenin protein and Wnt/β-catenin targets (Fig. S9C, D), and caused an increased cytoplasmic localization of β-catenin in HCT116 cells (Fig. S9E). Collectively, these results suggest that RRP15-dependent ribosome stress regulates Wnt/β-catenin signaling by modulating the subcellular localization and protein stability of β-catenin.

### RRP15 and β-catenin are co-expressed in CRC tissues and positively associated with poor prognosis of CRC patients

**Fig. 6:**
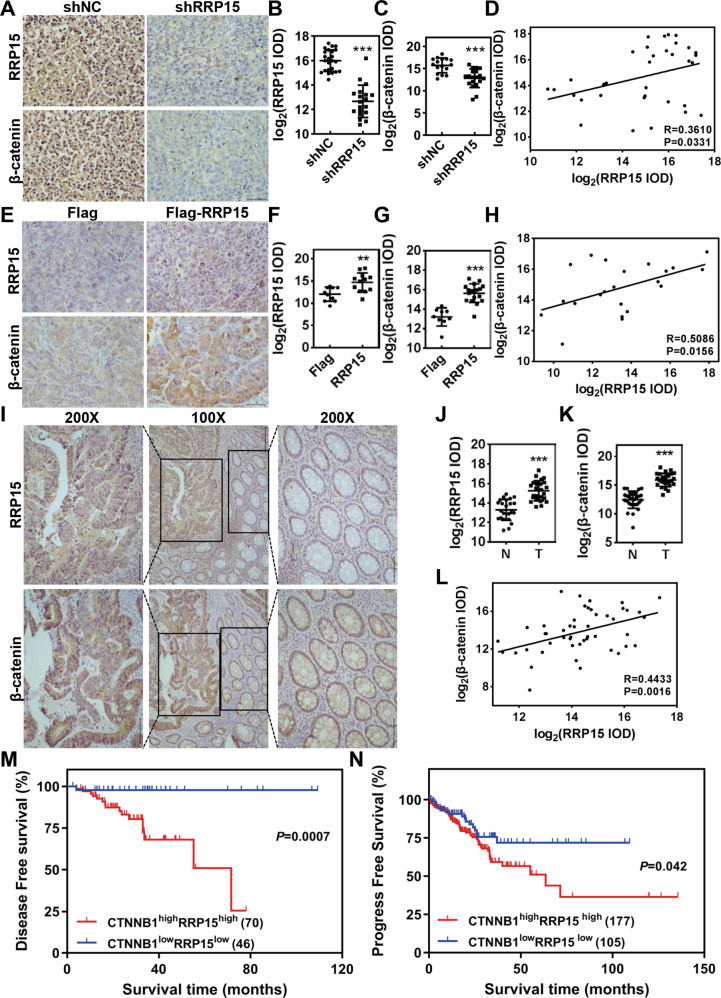
RRP15 and β-catenin are positively correlated in tissues and associated with prognosis of CRC patients. **A**–**H** Representative immunohistochemical staining images of RRP15 and β-catenin in tissues from indicated subcutaneous xenograft tumors. The immunostaining intensities of RRP15 and β-catenin were quantified by IOD and the relationship between β-catenin and RRP15 were analyzed. Three more random areas of each sample were measured. **I**–**L** Representative immunohistochemical staining images of RRP15 and β-catenin in tissues from CRC patients. The immunostaining intensities of RRP15 and β-catenin were quantified by IOD and the relationship between β-catenin and RRP15 were analyzed. Three more random cancerous or non-cancerous areas of each sample were measured. DFS (**M**) and PFS (**N**) of CRC patients from TCGA cohort with *CTNNB1*^high^/*RRP15*^high^ or *CTNNB1*^low^/*RRP15*^low^ were analyzed by Kaplan–Meier survival analysis. Scale bar: 100 μm. data are shown as mean ± standard deviations. **P < 0.01 and ***P < 0.001.

We further examined the relationship between RRP15 and β-catenin in xenograft and pathological tumor tissues. Results of IHC staining showed that the expression of β-catenin in xenograft tumor tissues was diminished when RRP15 KD (Fig. 6A–C), and a positive correlation between β-catenin and RRP15 expression was observed (Fig. 6D, R = 0.361, *P* = 0.0331). Reversely, β-catenin expression was significantly elevated in xenograft tumor tissues when RRP15 OE, and displayed positively associated with RRP15 expression (Fig. 6E–H, R = 0.5086, *P* = 0.0156). We further analyzed the expression of β-catenin and RRP15 in clinical CRC tissues. Similarly, β-catenin and RRP15 protein levels were upregulated in CRC tissues comparing with adjacent tissues, and their expression also was positively correlated (Fig. 6I–L, R = 0.4433, *P* = 0.0016). Consistently, TCGA data analysis showed that the mRNA levels of *CTNNB1* and *RRP15* were positively correlated in CRC tissues (Fig. S10, R = 0.41, *P* = 2.2e^−16^). Moreover, *CTNNB1*^high^/*RRP15*^high^ CRC patients had poor DFS and PFS survival ratios when compared with *CTNNB1*^low^/*RRP15*^low^ patients (Fig. 6M, N). Taken together, we demonstrate that RRP15 is positively related to β-catenin in CRC tissues, and their co-expression is served as predictive markers of prognosis in CRC patients.

### RRP15 regulates Wnt/β-catenin signaling pathway via ribosome stress-induced LZTS2 translation in CRC cells

**Fig. 7:**
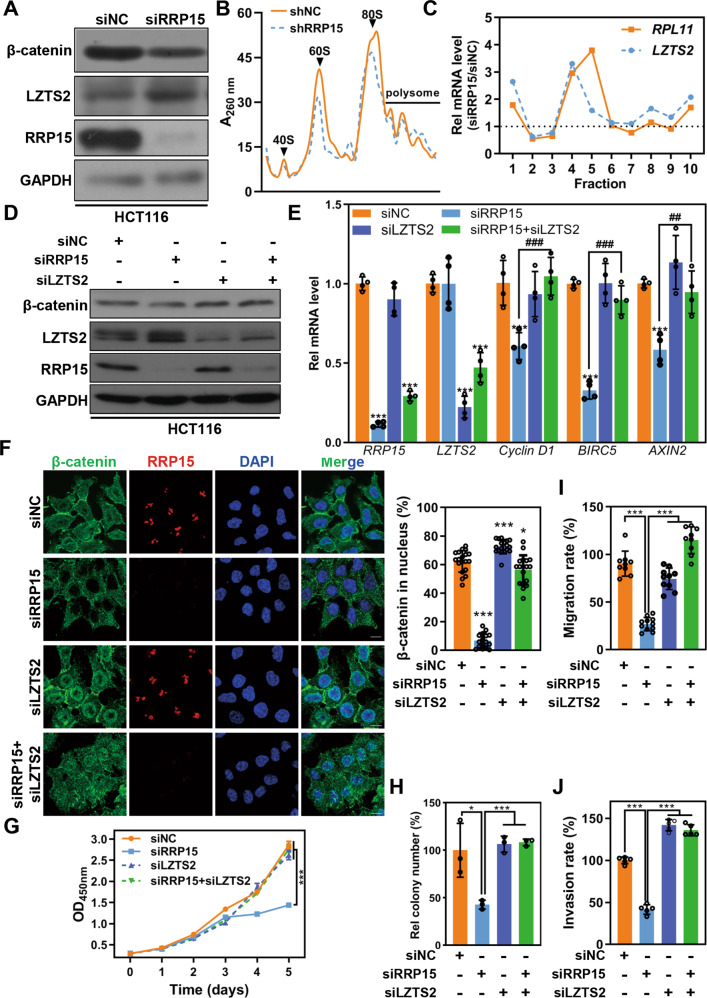
RRP15 regulates β-catenin expression and CRC progression depending on reprogramming LZTS2 translation. **A** Western blot for indicated proteins expression in siRRP15 or siNC transfected HCT116 cells. **B** Using sucrose gradient centrifuge to purify ribosome of indicated cells and the ribosome profile are showed. **C** Quantification of the distribution of the *LZTS2* mRNA and *RPL11* mRNA along polysome profiles in HCT116 cells with stably shRRP15 KD or controls. RNA was measured by qPCR and normalized to *Renilla luciferase* mRNA. **D** Western blot for β-catenin expression in HCT116 cells transfected with indicated siRNA. **E** qRT-PCR analysis of indicated Wnt/β-catenin downstream targets in HCT116 cells transfected with indicated siRNA. **F** Representative immunostaining images of RRP15 and β-catenin in HCT116 cells transfected with indicated siRNA. Relative β-catenin staining intensity in nucleus was counted and data were obtained from at least 20 cells. The effects of LZTS2 KD on RRP15-mediated cell proliferation capacity (**G**, **H**) and metastatic capacity (**I**, **J**) were measured. All experiments were performed at least three times. Data are shown as mean ± standard deviations. *P < 0.05, **P < 0.01, ***P < 0.001, ^##^*P* < 0.01, and ^###^*P* < 0.001.

RRP15 KD inhibited Wnt/β-catenin signaling in both p53 proficient and deficient CRC cells, while p53 co-deletion only partially reversed the inhibitory effects to Wnt/β-catenin targets transcription induced by RRP15 KD (Fig. S11). Therefore, we sought to explore other mechanism(s) by which RRP15 regulates Wnt/β-catenin signaling. Previous studies found that, upon impairment of 40S small subunits biogenesis, the affinity of ribosome with a group of mRNAs with a terminal oligopyrimidine (TOP mRNA) tract would increase, resulting in the upregulation of the corresponding proteins [23–25]. We analyzed the mRNA structures of negative regulators of Wnt/β-catenin signaling, and found that *LZTS2*, *DKK1* and *GSK3β* mRNA belong to TOP mRNA. RRP15 KD significantly enhanced the expression of LZTS2, DKK1 and GSK3β (Figs. 7A, S8A and S12A). However, knockdown of RPS6, a known component of 40S ribosome whose knockdown causes impairment of 40S biogenesis and activation of TOP mRNA translation [24], only induced a significant increase of LZTS2, but not DKK1 and GSK3β (Fig. S12A). Previous studies reported that LZTS2 may cause the export of β-catenin from nucleus [10, 26]. Therefore, we focused on investigating how RRP15 KD altered LZTS2 expression in CRC.

In RRP15 KD CRC cells, the mRNA level of *LZTS2* was not significantly changed (Fig. S12B), which implied that the translation of *LZTS2* may be upregulated upon RRP15 deficiency-induced ribosome stress. To test this hypothesis, we purified polysome from RRP15 KD and control cells lysate, and analyzed the distribution of *LZTS2* mRNA on polysome gradients. RRP15 KD markedly impaired the RiBi of both subunits in HCT116 cells (Fig. 7B). The analysis of mRNA distribution indicated that the amount of *LZTS2* mRNA on polysome gradients was significantly enhanced in RRP15 KD cells, which is consistent with another known TOP mRNA *RPL11* (Fig. 7C). Therefore, we hypothesized that RRP15 dependent impairment of RiBi stimulates the translation of TOP mRNA *LZTS2*, thereby negatively regulating Wnt/β-catenin signaling pathway to inhibit CRC progression.

**Fig. 8:**
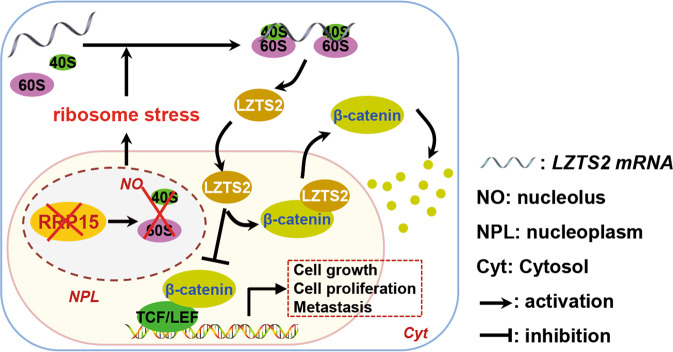
The model of RRP15 regulates CRC progression. RRP15 plays a critical role in regulating RiBi. RRP15 deficiency-induced severe ribosome stress enhances the affinity of TOP mRNA *LZTS2* with ribosome and promotes its translation, causing nuclear export and degradation of β-catenin, and thereby inhibiting cell proliferation and metastasis of CRC.

To further test the hypothesis, we explored the effects of LZTS2 KD on RRP15-dependent Wnt/β-catenin inhibition and cellular phenotype. Although no significant change observed in p53 and p21, co-transfection of siRRP15 and siLZTS2 effectively reversed RRP15 KD-induced β-catenin nuclear export and down-regulation (Figs. 7D–F and S13A), as well as considerably rescued RRP15 KD-mediated inhibition of proliferative and metastatic activity in CRC cells (Figs. 7G–J and S13B–E). Taken together, our results demonstrate that RRP15 deficiency-induced ribosome stress enhances the affinity of TOP mRNA *LZTS2* with ribosome and promotes its translation, causing nuclear export and degradation of β-catenin, and thereby inhibiting cell proliferation and metastasis of CRC (Fig. 8).

## Discussion

In this study, we reveal that RRP15 is significantly upregulated in CRC tissues and its high expression positively is associated with poor prognosis of CRC patients. RRP15 positively regulates Wnt/β-catenin signaling pathway which is crucial for CRC proliferation and metastasis. Mechanistically, we demonstrate that, RRP15 deficiency induced ribosome stress reprogram protein translational profile to enhance the expression of LZTS2, which thereby contributed to nuclear export and degradation of β-catenin.

RiBi is a crucial process for cell growth, proliferation, metabolism and stress response, etc. Dysregulation of RiBi and parallel protein synthesis are frequent events in CRC. It has been reported that multiple RiBi-related proteins are aberrant in CRC and involve in regulating cell proliferation, metastasis, metabolism, and chemoresistance. For example, Myc induces transcription mediated by Pol I, II, and III, and plays a critical role in rDNA transcription, pre-rRNA processing, and ribosome assembly [27, 28]. Myc is almost universally amplified or overexpressed in CRC due to *APC* mutation and drives malignant transformation of CRC through regulating growth, proliferation, metabolism, angiogenesis, DNA repair, and protein synthesis [29, 30]. In addition, 45S rRNA, RPL15, RPS24, POLR1D, and GNL3 are upregulated in CRC and promote CRC progression [31–36]. These evidences definitely support the opinion that CRC is a kind of cancer with hyperactive RiBi which render CRC more vulnerable to several agents targeting to RiBi. Indeed, oxaliplatin, a fundamental chemotherapeutic agent for clinical treatment of CRC and other gastrointestinal cancers, kills CRC cells by inducing ribosome stress [37]. In our study, we found that RRP15 is overexpressed in CRC tissues and cell lines. Given RRP15 play a crucial role in RiBi, we can speculate that, due to hyperactive RiBi and accompanied translation addiction, CRC patients with upregulated RRP15 might more sensitive to ribosome stress inducer. Consistent with this speculation, our results revealed that RRP15 KD could specifically induce apoptosis in CRC cells but not normal epithelial cells.

Past two decades, numerous shreds of evidences have demonstrated that interruption of RiBi by extra- or endocellular stimuli would lead to severe IRBC. IRBC triggers the release of RPL11, RPL5, and 5S rRNA from ribosome subunits and perform a complex with E3 ubiquitin-protein ligase HDM2, thereby inhibiting ubiquitination and degradation of p53, by which induces cell cycle arrest, apoptosis or senescence [38–43]. In addition, several studies reported that IRBC can trigger p53-independent cell response via regulating critical proteins related to carcinogenesis including p73, c-Myc, E2F1, ATF4 [20, 44–48]. Zhao et al. had demonstrated that RRP15 KD triggers IRBC and cell senescence in p53 wild-type HepG2 cells, but induces metabolic reprogramming and apoptosis in p53 mutated and depleted cells [18]. Here, our results revealed that RRP15 KD caused cell cycle arrest at G2/M and apoptosis in HCT116 cells with wild-type p53, and p53 KD only partially reverse RRP15-induced proliferative inhibition and apoptosis, which indicates that the classical IRBC pathway plays a partial role in regulating cell proliferation and apoptosis in response to RRP15-mediated ribosome stress. However, considering that similar phenotypes were observed in RRP15 depleted SW620 cells, as well as p53 KD did not effectively rescue the migratory and Wnt//β-catenin signaling pathway inhibition mediated by RRP15 KD, we speculate, beyond canonical IRBC, there may be p53-independent checkpoint/signaling pathway that plays a vital role in response to RRP15-mediated ribosome stress. Here, we find a novel IRBC mechanism by which RRP15 deficiency induced ribosome stress regulates Wnt/β-catenin signaling pathway via reprogramming protein translation of TOP mRNA *LZTS2*.

The response to various extra- or endocellular requires the coordination of gene networks to achieve homeostasis in energy and resource. Therefore, the translation of protein is precisely controlled by different approaches. Among them, TOP mRNAs are the primary targets of translational control under stress. Under normal conditions, the synthesis of TOP mRNA is coordinate with cellular demands. However, under adverse conditions, such as impairment of 40S ribosome subunit biogenesis, hypoxia, or amino acid deprivation, the affinity of TOP mRNA with translational apparatus would enhance in parallel with protein synthesis increase [24, 49]. Consistent with our previous study [16], we showed that RRP15 KD induced severe ribosome stress and impairment in both 40S and 60S ribosome subunits biogenesis. Upon RRP15 KD induced ribosome stress, the expression of several Wnt/β-catenin negative regulators with TOP structure including LZTS2, DKK1, and GSK3β were increased. Especially, we further found that the affinity of *LZTS2* mRNA with polysome is significantly increased when RRP15 KD. Given the nuclear export activity of LZTS2, RRP15 KD could significantly increase nuclear export of β-catenin dependent on LZTS2 expression [10]. However, we cannot completely rule out the possibility that several other signaling pathways (eg. AMPK, NF-κB, or HIF-1α) involved in responding to ribosome stress may regulate LZTS2 expression indirectly [50].

In addition, we did not observe any significant alteration of LZTS2 expression in RRP15 OE cells (data not shown), suggesting that increased β-catenin expression and nuclear localization in RRP15 OE cells are not dependent on LZTS2. Several studies also found other mechanisms for Wnt/β-catenin activation by RiBi-related proteins. For example, Huang et al. found that overexpression of ribosomal protein RPL15 selectively enhanced the translation of β-catenin [51]. TC1 (thyroid cancer1) activated Wnt/β-catenin signaling pathway via interacting with Chibby and releasing the inhibitory function of Chibby on β-catenin [51].

## Conclusions

We demonstrate that RRP15 is upregulated in CRC cell lines along with tumor tissues, and can be used to predict the prognosis of CRC patients. Moreover, we find a novel IRBC response by which RRP15 deficiency induced ribosome stress inhibits cell proliferation and metastasis via LZTS2-mediated Wnt/β-catenin signaling suppression.

### Supplemental data

The supplementary material exists online.

## Methods

### Patients and CRC tissues samples

The tissue microarray (TMA) was obtained from Xuzhou Medical University, which contained 309 cases of CRC tumor tissues and paired adjacent non-cancerous tissues from the CRC patient cohort that was recruited from the Affiliated Hospital of Xuzhou Medical University (China) with informed consent, as approved by the Research Ethics Committee. In addition, 28 patients with stage I-IV CRC from The First Affiliated Hospital of Wenzhou Medical University were enrolled and their tumor tissues were used for qRT-PCR or IHC to analyze the expression of *RRP15* mRNA and the correlation between β-catenin and RRP15.

### Immunohistochemistry (IHC)

IHC assays were performed as previously described [52]. For primary antibody incubation, rabbit anti-RRP15 sera were applied at 1: 50 dilution, Rabbit anti-β-catenin antibodies at 1:1000 dilution and Rabbit anti-Ki67 antibody at 1:10,000 dilution. The staining of RRP15 was scored via combining the percentage of cells with the staining intensity and being dependent on the IRS (immunoreactive score). The intensity of RRP15 immunostaining was scored as 0–3 (0, negative; 1, weak; 2, moderate; 3, strong); the percentage of immunoreactivity cells was graded as 1 (0–25%), 2 (26–50%), 3 (51–75%), and 4 (76–100%). The level of RRP15 expression was categorized as low (IRS: 0–6) and high (IRS: 8–12). The intensity of β-catenin and Ki67 immunostaining were qualified by IOD (Integrated option density) using Image-Pro Plus 6.0 software.

### Plasmids, siRNAs, and antibodies

Mammalian expression plasmids of *RRP15* were generated as described previously [16]. All constructs were fully sequenced. Nonsense control siRNA (siNC: 5′-TTCTCCGAACGTGTCACGTTT-3′) and siRNAs specific targeting to 3′-UTR (siRRP15: 5′-AAATGGTAACTGGAGCCGTA-3′) or CDS (siRRP15-CDS: 5′-CCTGAAAGTAAACCTACTATT-3′) of RRP15 were synthesized by Genepharma (Shanghai, China).

Polyclonal rabbit anti-RRP15 antibodies were generated as previously described [16]. Rabbit anti-cyclin B1 antibodies (55004-1-AP), rabbit anti-PCNA antibodies (10205-2-AP), rabbit anti-E-cadherin antibodies (20874-1-AP), rabbi anti-Vimentin antibodies (10366-1-AP), rabbit anti-N-cadherin antibodies (22018-1-AP), rabbit anti-MMP9 antibodies (10375-2-AP), rabbit anti-β-catenin antibodies (51067-2-AP), rabbit anti-LZTS2 antibodies (15677-1-AP), rabbit anti-DKK1 antibodies (21112-1-AP), mouse anti-β-catenin antibody (66379-1-Ig), rabbit anti-GSK3β antibodies (22104-1-AP), rabbit anti-Ki67 antibodies (27309-1-AP) and mouse anti-GAPDH antibody (60004-1-Ig) were purchased from Proteintech (Rosemont, USA). Mouse anti-γH2AX antibody (05-636), mouse anti-Flag antibody (F1804), mouse anti-α-tubulin antibody (T5168), and mouse anti-GFP antibody (G6539) were purchased from Sigma Aldrich (Billerica, USA). Mouse anti-p53 antibody (sc-126) and mouse anti-p21 antibody (sc-6246) were purchased from Santa Cruz Biotechnology (Texas, USA). Rabbit anti-cleaved caspase 7 antibodies (8438), mouse anti-caspase 9 antibody (9508), rabbit anti-cyclin D1 antibody (55506), rabbit anti-CDK2 antibody (18048), mouse anti-cyclin E1 antibody (4129) and rabbit anti-H3 antibody (4499) were purchased from Cell Signaling Technology (Boston, USA). All secondary antibodies were obtained from Life Technologies Inc (Massachusetts, USA).

### Cell culture, transfection, and drug treatment

FHC cells were provided by Dr. Pingfu Hou (Xuzhou Medical University). All CRC cell lines were purchased from the American Type Culture Collection (ATCC, Virginia, USA), where these cell lines were characterized by DNA fingerprinting and isozyme detection. FHC, SW480, SW620 and LoVo cells are cultured in RPMI 1640 medium (Gibco, California, USA) supplemented with 10% fetal bovine serum (FBS, Gibco). HCT116 and HT-29 cells were cultured in DMEM medium (Gibco) supplemented with 10% FBS. Caco-2 cells were cultured in MEM medium (Gibco) supplemented with 20% FBS and nonessential amino acids (NEAA, Gibco). All cells were cultured at 37 °C in 5% CO_2_. MG132 (Sigma aldrich), cycloheximide (CHX, Shanghai, China), or actinomycin D (a gift from Dr. Linyong Du of Wenzhou Medical University) treatment consisted of addition of drugs at indicated concentration for indicated time. Plasmids and siRNAs transfection were conducted with Lipofectamine 3000 (Life Technologies Inc.) according to the manufacturer’s protocol.

### Establishment of stably transfected cell lines

*RRP15* cDNA was cloned into the pCDH1-CMV-MSC-EF1-Flag-Puro vector and RRP15 shRNA or scrambled sequences were cloned into the pLKO.1 vector. To obtain lentiviruses, RRP15 overexpression/knockdown or empty vector was transfected into 293T together with the packing plasmids (psPAX and pMD2G) for producing viral particles using calcium phosphate transfection. Then, stably transfected cell lines were generated by infecting with the lentivirus and selected with puromycin for 2 days. Each shRNA and corresponding siRNA share the same core sequence.

### Western blot and immunofluorescence analyses

Cells were harvested or fixed for Western blot or immunofluorescence analysis as previously described [35]. In brief, for Western blot, cells were harvested and lysed in RIPA buffer. Cell lysates with equal amount of total protein were subjected to SDS-PAGE, transferred to PVDF membrane and then immunoblotted with corresponding antibodies. For immunofluorescence analysis, cells grown on glass coverslips were fixed and F with indicated antibodies. Cells were photographed using a NIKON confocal microscope (Nikon A1, Tokyo, Japan).

### RNA isolation and quantitative real-time PCR (qRT-PCR)

Total RNA was isolated using the TRIzol reagent (Life Technologies Inc.) following manufacturer’s instructions. One microgram RNA was used for cDNA synthesis using a reverse transcriptase reaction kit (Promega, Wisconsin, USA). qPCR was performed on a Biorad CFX 96 Touch using SYBR Green (TIANGEN BIOTECH, Beijing, China) as a dsDNA-specific fluorescent dye. GAPDH was used for standardizing indicated mRNA level. Amplification primers were synthesized as follow: 5′-GGTAACTGGAGCCGTAG-3′ (forward) and 5′-GGACTTTAGCCATAGCAT-3′ (reverse) for *RRP15*, 5′-TCGAGCGTTCGCGTTCAG-3′ and 5′-GAGTGAGACGAGACGAGACGC-3′ for *47S rRNA*, 5′-CATTGAACACTTCCTCTCCA-3′ (forward) and 5′-AACTTCACATCTGTGGCAC-3′ (reverse) for *cyclin D1*, 5′-CATTCGTCCGGTTGCGCTTTCC-3′ (forward) and 5′-GCGCACTTTCTCCGCAGTTTCC-3′ (reverse) for *BIRC5*, 5′-TTATGCTTTGCACTACGTCCCTCCA-3′ (forward) and 5′-CGCAACATGGTCAACCCTCAGAC-3′ (reverse) for *AXIN2*, 5′-CCGAGGCTTGAGGAGACCA-3′ (forward) and 5′-TGTAGACCCCGCGCACG-3′ (reverse) for *LZTS2*, 5′-GCCTCTTCTTATTTATGG-3′ (forward) and 5′-AAGAACCATTACCAGATTT-3′ (reverse) for *Renilla luciferase*, 5′-CCTGGCACCCAGCACAAT-3′ (forward) and 5′-GCCGATCCACACGGAGTACT-3′ (reverse) for *β-actin*, and 5′-TTCATTGACCTCAACTACATGGTTTAC-3′ (forward) and 5′-TGACAAGCTTCCCGTCTCA-3′ (reverse) for *GAPDH*. Data were analyzed using the 2^-ΔΔCt^ method [53].

### Cell proliferation, cell cycle analysis, and apoptosis assay

3000 of RRP15 stably overexpressed/knockdown cells or control cells were seeded on 96-well plates, or 1000 cells of HCT116 or SW620 were seeded on 96-well plates for 24 h and transfected with indicated siRNA. The cells proliferation was determined using CCK8 kit at indicated time according to the manufacturer’s protocol (Dojindo, Kyushu, Japan). Cell viability was determined by measuring the absorbance at 450 nm. For colony formation assay was performed in 6-wells dish in which 300 cells were seeded and cultured for 10 days. Colonies were counted manually after staining with 0.1% crystal violet.

For cell cycle analysis, stably transfected cells or cells transfected with siRNA were fixed in 70% ethanol/30% PBS for overnight at −20 °C. The cells were subsequently washed once with PBS, and incubated in propidium iodide (PI) buffer (60 g/ml PI and 0.1 mg/ml RNase A) for 45 min at room temperature. Cells were analyzed by Fluorescent-activated cell sorting (FACS) (BD FACSVerse system, New Jersey, USA) and at least 10,000 cells per condition were collected. Cell cycle profiles were processed and analyzed using FlowJo version 6.4.7.

For apoptosis assay, stably transfected cells or cells transfected with siRNA were harvested, and then stained as described in the Annexin V FITC Apoptosis Detection Kit (KeyGen Biotech, Nanjing, China). Data were acquired using a BD FACSVerse system.

### Cell migration and invasion assay

Cell migration and invasion assays were performed as previously described [54]. In brief, for wound healing assay, RRP15 stably overexpressed/knockdown cells or control cells (1 × 10^6^ cells per well) were seeded on 6-well plates. 24 h later, cells were starved overnight in serum-free medium. Then the cells in layers were scratched using a sterile 200 μl pipette tip, then washed with PBS and cultured in serum-free medium with 1% bovine serum albumin. The progress of migration was photographed immediately and 3 days after wounding.

For transwell migration and invasion assays, 1 × 10^5^ starved cells in serum-free medium were added to the top chambers of migration device (353097, Corning, NY, USA) and invasion device (354480, Corning), media containing 20% FBS were added to the bottom chambers. After 24 h incubation, top (nonmigrated) cells were removed, and bottom (migrated) cells were fixed and stained with crystal violet. The number of migrating cells in 5 fields was counted under ×10 magnification, and the means for each chamber were determined.

### Tumor growth and metastasis assay in vivo

All animal experiments were approved by the Animal Ethics Committee of the Animal Laboratory of Wenzhou Medical University, and performed in the SPF environment of the Animal Center of Wenzhou Medical University. For tumor growth assay, 5 × 10^6^ RRP15 stably overexpressed/knockdown cells or control cells were transplanted s.c. into the flank of each 3–5 weeks’ old female athymic mouse (each group *n* = 6). Tumors were measured per three days using a Vernier caliper, and tumor volume (*V*) was calculated using the equation *V* = 1/2*ab*^2^, where *a* and *b* represent the length and width, respectively (in millimeters). For metastasis assay, 1.5 × 10^6^ RRP15 stable knockdown cells or control cells were injected into each mouse via the tail vein (each group *n* = 5). The nude mice were sacrificed by euthanasia, and the lungs were isolated for counting the metastatic nodules. The investigator was blinded to the group allocation of the animals during the experiment.

### RNA sequencing (RNA-seq) analysis

RNA-seq was performed by Anoroad. siRRP15 and siNC transfected cells were used for sequencing. Total RNA was extracted with TRIzol reagent and RNA quality was assessed via agrose gel electrophoresis. cDNA libraries for Illumina sequencing were conducted, and data from three biological replicates were further analyzed.

### Analysis of protein stability

1 × 10^5^ RRP15 knockdown cells or control cells were plated on 12-well plate and cultured for 24 h. The cells were pretreated with 10 μM MG132 for 6 h, and subsequently were treated with CHX at 20 μM for 0, 4, 8, 12 h. The cells were collected and lysed with RIPA buffer, and equal amount of total protein were used for western blot with indicated antibodies.

### Separation of the cytoplasmic and nuclear fractions

Subcellular fraction separation was performed as described previously [35]. In brief, RRP15 stably overexpressed/knockdown cells or control cells were swollen in ice-cold hypotonic buffer, and then were lysed with hypotonic lysis buffer (10 mM Hepes-NaOH [pH 7.5], 10 mM NaCl, 2 mM MgCl_2_, 1 mM EDTA, 1 μg of leupeptin/ml, 1 μg of aproptinin/ml, 50 μg of PMSF/ml, 1 mM Na_3_VO_4_, 1 mM NaF, 0.3% NP-40, and 0.2% sodium deoxycholate). The nuclei pellet was collected by centrifugation at 2800 × *g* for 5 min, supernatant reserved for cytoplasmic fraction.

### Polysome purification and protein translation assay

Polysome purification and RNA extraction were performed as described earlier [16, 25]. In brief, 3 × 10^6^ RRP15 depleted HCT116 cells and control cells were treated with CHX at 37 °C for 5 min at a concentration of 90 μg/ml. Cells were washed twice with cold PBS supplemented with CHX, scraped on ice, and centrifuged at 4000 rpm for 5 min. Cell pellets were resuspended in 500 μl of hypotonic lysis buffer (130 mM KCl, 10 mM MgCl_2_, 20 mM Tris HCl pH 7.2, 1 mM DTT, 0.5% sodium deoxycholate, 0.5% NP-40, 0.2 mg/ml heparin) supplemented with protease inhibitors (Roche, Basel, Switzerland) and RNase inhibitor (Thermo fisher, Massachusetts, USA) at a concentration of 100 U/ml and left in ice for 15 min. Cell lysates were cleared of debris and nuclei by centrifugation for 10 min at 8000 × *g*. Protein concentrations were determined by BCA assay and 500 mg of lysate were loaded on 10%–50% sucrose linear gradients containing 130 mM KCl, 10 mM MgCl_2_, 20 mM Tris HCl pH 7.2, 1 mM DTT, 0.2 mg/ml of heparin. Gradients were centrifuged on a SW41 rotor for 2.5 h at 36,000 rpm. The gradients were collected downward and the absorbance at 260 nm was measured. Polysome fractions were supplemented with SDS at a final concentration of 1% and placed for 10 min at 65 °C. One nanogram of *Renilla luciferase* mRNA was added to each polysome fraction for normalization. Samples were treated with protein K for 45 min at 50 °C, followed by phenol-chloroform extraction and precipitation with isopropanol. Purified RNAs from each fraction were reverse transcribed and subjected to qRT-PCR. mRNA quantification was normalized to *Renilla luciferase* mRNA.

### Statistical analysis

All the statistical analyses were performed by Graphpad Prism version 8.0 (GraphPad Software, California, USA) and SPSS 20.0 statistical software (SPSS Inc., Chicago, USA). The paired Wilcoxon test was used to assess the significance of RRP15 staining in cancers and their coupled adjacent non-cancerous tissues. Fisher’s exact test was performed to evaluate the association between RRP15 expression and clinicopathological parameters. Pearson correlation analyses were conducted to investigate the correlation between RRP15 and β-catenin expression. Probability of differences in OS, DFS, and PFS as a function of time was verified by Kaplan–Meier method and log-rank test. Student *t*-test was used to analyze the difference between two groups. *P* value < 0.05 was considered as statistically significant.

## Supplementary information

supplemental data

Original Data File

Author checklist

## Data Availability

The datasets generated in the current study are available from the corresponding authors on reasonable request.

## References

[1] Sung H, Ferlay J, Siegel RL, Laversanne M, Soerjomataram I, Jemal A (2021). Global cancer statistics 2020: GLOBOCAN estimates of incidence and mortality worldwide for 36 cancers in 185 countries. CA Cancer J Clin.

[2] Allemani C, Matsuda T, Di Carlo V, Harewood R, Matz M, Niksic M (2018). Global surveillance of trends in cancer survival 2000-14 (CONCORD-3): analysis of individual records for 37 513 025 patients diagnosed with one of 18 cancers from 322 population-based registries in 71 countries. Lancet.

[3] Nguyen LH, Goel A, Chung DC (2020). Pathways of colorectal carcinogenesis. Gastroenterology.

[4] Cancer Genome Atlas Network. (2012). Comprehensive molecular characterization of human colon and rectal cancer. Nature.

[5] Zhang N, Wei P, Gong A, Chiu WT, Lee HT, Colman H (2011). FoxM1 promotes beta-catenin nuclear localization and controls Wnt target-gene expression and glioma tumorigenesis. Cancer Cell.

[6] Townsley FM, Thompson B, Bienz M (2004). Pygopus residues required for its binding to Legless are critical for transcription and development. J Biol Chem.

[7] Henderson BR (2000). Nuclear-cytoplasmic shuttling of APC regulates beta-catenin subcellular localization and turnover. Nat Cell Biol.

[8] Cong F, Varmus H (2004). Nuclear-cytoplasmic shuttling of Axin regulates subcellular localization of beta-catenin. Proc Natl Acad Sci USA.

[9] Hendriksen J, Fagotto F, van der Velde H, van Schie M, Noordermeer J, Fornerod M (2005). RanBP3 enhances nuclear export of active (beta)-catenin independently of CRM1. J Cell Biol.

[10] Thyssen G, Li TH, Lehmann L, Zhuo M, Sharma M, Sun Z (2006). LZTS2 is a novel beta-catenin-interacting protein and regulates the nuclear export of beta-catenin. Mol Cell Biol.

[11] Montanaro L, Trere D, Derenzini M (2008). Nucleolus, ribosomes, and cancer. Am J Pathol.

[12] Khot A, Brajanovski N, Cameron DP, Hein N, Maclachlan KH, Sanij E (2019). First-in-human RNA polymerase I transcription inhibitor CX-5461 in patients with advanced hematologic cancers: results of a phase I dose-escalation study. Cancer Disco.

[13] Xu H, Di Antonio M, McKinney S, Mathew V, Ho B, O’Neil NJ (2017). CX-5461 is a DNA G-quadruplex stabilizer with selective lethality in BRCA1/2 deficient tumours. Nat Commun.

[14] De Marchis ML, Giorgi A, Schinina ME, Bozzoni I (2005). Fatica A. Rrp15p, a novel component of pre-ribosomal particles required for 60S ribosome subunit maturation. RNA.

[15] Tafforeau L, Zorbas C, Langhendries JL, Mullineux ST, Stamatopoulou V, Mullier R (2013). The complexity of human ribosome biogenesis revealed by systematic nucleolar screening of Pre-rRNA processing factors. Mol Cell.

[16] Dong Z, Zhu C, Zhan Q, Jiang W (2017). The roles of RRP15 in nucleolar formation, ribosome biogenesis and checkpoint control in human cells. Oncotarget.

[17] Wu T, Ren MX, Chen GP, Jin ZM, Wang G (2016). Rrp15 affects cell cycle, proliferation, and apoptosis in NIH3T3 cells. FEBS Open Bio.

[18] Zhao D, Qian L, Zhuang D, Wang L, Cao Y, Zhou F (2021). Inhibition of ribosomal RNA processing 15 Homolog (RRP15), which is overexpressed in hepatocellular carcinoma, suppresses tumour growth via induction of senescence and apoptosis. Cancer Lett.

[19] Yu W, Qiu Z, Gao N, Wang L, Cui H, Qian Y (2011). PAK1IP1, a ribosomal stress-induced nucleolar protein, regulates cell proliferation via the p53-MDM2 loop. Nucleic Acids Res.

[20] Iadevaia V, Caldarola S, Biondini L, Gismondi A, Karlsson S, Dianzani I (2010). PIM1 kinase is destabilized by ribosomal stress causing inhibition of cell cycle progression. Oncogene.

[21] Fumagalli S, Ivanenkov VV, Teng T, Thomas G (2012). Suprainduction of p53 by disruption of 40S and 60S ribosome biogenesis leads to the activation of a novel G2/M checkpoint. Genes Dev.

[22] Perry RP, Kelley DE (1970). Inhibition of RNA synthesis by actinomycin D: characteristic dose-response of different RNA species. J Cell Physiol.

[23] Loreni F, Thomas G, Amaldi F (2000). Transcription inhibitors stimulate translation of 5’ TOP mRNAs through activation of S6 kinase and the mTOR/FRAP signalling pathway. Eur J Biochem.

[24] Fumagalli S, Di Cara A, Neb-Gulati A, Natt F, Schwemberger S, Hall J (2009). Absence of nucleolar disruption after impairment of 40S ribosome biogenesis reveals an rpL11-translation-dependent mechanism of p53 induction. Nat Cell Biol.

[25] Gentilella A, Moron-Duran FD, Fuentes P, Zweig-Rocha G, Riano-Canalias F, Pelletier J (2017). Autogenous control of 5’TOP mRNA Stability by 40S ribosomes. Mol Cell.

[26] Li Y, Li Q, Long Y, Cui Z (2011). Lzts2 regulates embryonic cell movements and dorsoventral patterning through interaction with and export of nuclear beta-catenin in zebrafish. J Biol Chem.

[27] van Riggelen J, Yetil A, Felsher DW (2010). MYC as a regulator of ribosome biogenesis and protein synthesis. Nat Rev Cancer.

[28] Destefanis F, Manara V, Bellosta P (2020). Myc as a regulator of ribosome biogenesis and cell competition: a link to cancer. Int J Mol Sci.

[29] Gabay M, Li Y, Felsher DW (2014). MYC activation is a hallmark of cancer initiation and maintenance. Cold Spring Harb Perspect Med.

[30] Hon KW, Zainal Abidin SA, Othman I, Naidu R (2021). The crosstalk between signaling pathways and cancer metabolism in colorectal cancer. Front Pharm.

[31] Tsoi H, Lam KC, Dong Y, Zhang X, Lee CK, Zhang J (2017). Pre-45s rRNA promotes colon cancer and is associated with poor survival of CRC patients. Oncogene.

[32] Wang Y, Sui J, Li X, Cao F, He J, Yang B (2015). RPS24 knockdown inhibits colorectal cancer cell migration and proliferation in vitro. Gene.

[33] Wang M, Niu W, Hu R, Wang Y, Liu Y, Liu L (2019). POLR1D promotes colorectal cancer progression and predicts poor prognosis of patients. Mol Carcinog.

[34] Tang X, Zha L, Li H, Liao G, Huang Z, Peng X (2017). Upregulation of GNL3 expression promotes colon cancer cell proliferation, migration, invasion and epithelial-mesenchymal transition via the Wnt/beta-catenin signaling pathway. Oncol Rep.

[35] Dong Z, Jiang H, Liang S, Wang Y, Jiang W, Zhu C (2019). Ribosomal protein L15 is involved in colon carcinogenesis. Int J Med Sci.

[36] Pogue-Geile K, Geiser JR, Shu M, Miller C, Wool IG, Meisler AI (1991). Ribosomal protein genes are overexpressed in colorectal cancer: isolation of a cDNA clone encoding the human S3 ribosomal protein. Mol Cell Biol.

[37] Bruno PM, Liu Y, Park GY, Murai J, Koch CE, Eisen TJ (2017). A subset of platinum-containing chemotherapeutic agents kills cells by inducing ribosome biogenesis stress. Nat Med.

[38] Donati G, Peddigari S, Mercer CA, Thomas G (2013). 5S ribosomal RNA is an essential component of a nascent ribosomal precursor complex that regulates the Hdm2-p53 checkpoint. Cell Rep.

[39] Bursac S, Brdovcak MC, Pfannkuchen M, Orsolic I, Golomb L, Zhu Y (2012). Mutual protection of ribosomal proteins L5 and L11 from degradation is essential for p53 activation upon ribosomal biogenesis stress. Proc Natl Acad Sci USA.

[40] Dai MS, Lu H (2004). Inhibition of MDM2-mediated p53 ubiquitination and degradation by ribosomal protein L5. J Biol Chem.

[41] Zhang Y, Wolf GW, Bhat K, Jin A, Allio T, Burkhart WA (2003). Ribosomal protein L11 negatively regulates oncoprotein MDM2 and mediates a p53-dependent ribosomal-stress checkpoint pathway. Mol Cell Biol.

[42] Lohrum MA, Ludwig RL, Kubbutat MH, Hanlon M, Vousden KH (2003). Regulation of HDM2 activity by the ribosomal protein L11. Cancer Cell.

[43] Sloan KE, Bohnsack MT, Watkins NJ (2013). The 5S RNP couples p53 homeostasis to ribosome biogenesis and nucleolar stress. Cell Rep.

[44] Donati G, Brighenti E, Vici M, Mazzini G, Trere D, Montanaro L (2011). Selective inhibition of rRNA transcription downregulates E2F-1: a new p53-independent mechanism linking cell growth to cell proliferation. J Cell Sci.

[45] Zhou X, Hao Q, Zhang Q, Liao JM, Ke JW, Liao P (2015). Ribosomal proteins L11 and L5 activate TAp73 by overcoming MDM2 inhibition. Cell Death Differ.

[46] Dai MS, Arnold H, Sun XX, Sears R, Lu H (2007). Inhibition of c-Myc activity by ribosomal protein L11. EMBO J.

[47] Liao JM, Zhou X, Gatignol A, Lu H (2014). Ribosomal proteins L5 and L11 co-operatively inactivate c-Myc via RNA-induced silencing complex. Oncogene.

[48] Zhou X, Hao Q, Liao JM, Liao P, Lu H (2013). Ribosomal protein S14 negatively regulates c-Myc activity. J Biol Chem.

[49] Cockman E, Anderson P, Ivanov P (2020). TOP mRNPs: molecular mechanisms and principles of regulation. Biomolecules.

[50] Hua L, Yan D, Wan C, Hu B (2022). Nucleolus and nucleolar stress: from cell fate decision to disease development. Cells.

[51] Huang Y, Li Z, Lin E, He P, Ru G (2021). Oxidative damage-induced hyperactive ribosome biogenesis participates in tumorigenesis of offspring by cross-interacting with the Wnt and TGF-beta1 pathways in IVF embryos. Exp Mol Med.

[52] Zhang R, Liu S, Gong B, Xie W, Zhao Y, Xu L (2022). Kif4A mediates resistance to neoadjuvant chemoradiotherapy in patients with advanced colorectal cancer via regulating DNA damage response. Acta Biochim Biophys Sin.

[53] Livak KJ, Schmittgen TD (2001). Analysis of relative gene expression data using real-time quantitative PCR and the 2(-Delta Delta C(T)) method. Methods.

[54] Kong C, Wang C, Wang L, Ma M, Niu C, Sun X (2011). NEDD9 is a positive regulator of epithelial-mesenchymal transition and promotes invasion in aggressive breast cancer. PLoS ONE.

