# Supplementary material for: RRP15 deficiency induces ribosome stress to inhibit colorectal cancer proliferation and metastasis via LZTS2-mediated β-catenin suppression
Source: Cell Death Dis. 2023 Feb 7;14(2):89. doi: 10.1038/s41419-023-05578-6 (PMC9905588; doi:10.1038/s41419-023-05578-6)
Supplement: Supplementary file 1 — supplemental data [file 41419_2023_5578_MOESM1_ESM.docx]

**Supplemental figures:**


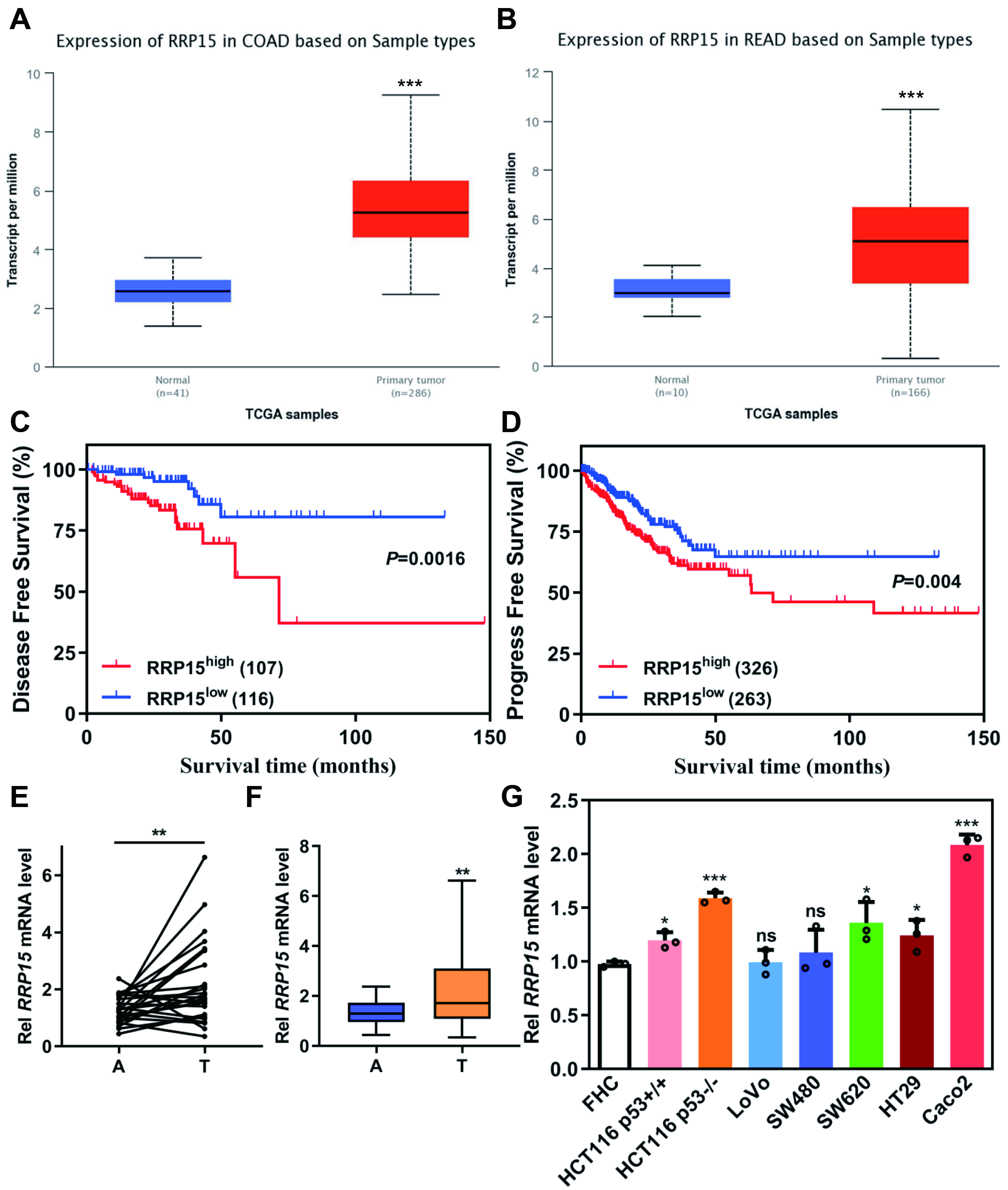


**Figure S1 RRP15 is upregulated in CRC and correlates with prognosis of CRC patients.** (A-B) TCGA data analysis of *RRP15* mRNA expression in tumor samples of COAD (A) and READ (B) when compared with normal samples. TCGA data analysis of the relationship between RRP15 expression with DFS (C) and PFS (D) of CRC patients. (E-F) qRT-PCR analysis of *RRP15* mRNA expression in tumor tissues as compared to surrounding normal tissues (n=20). (G) qRT-PCR analysis of RRP15 mRNA expression in CRC cells and a normal colon epithelial cell line (FHC). The experiments were repeated three times independently. Data are shown as mean ± standard deviations. **P*<0.05, ***P*<0.01 and ****P*<0.001.


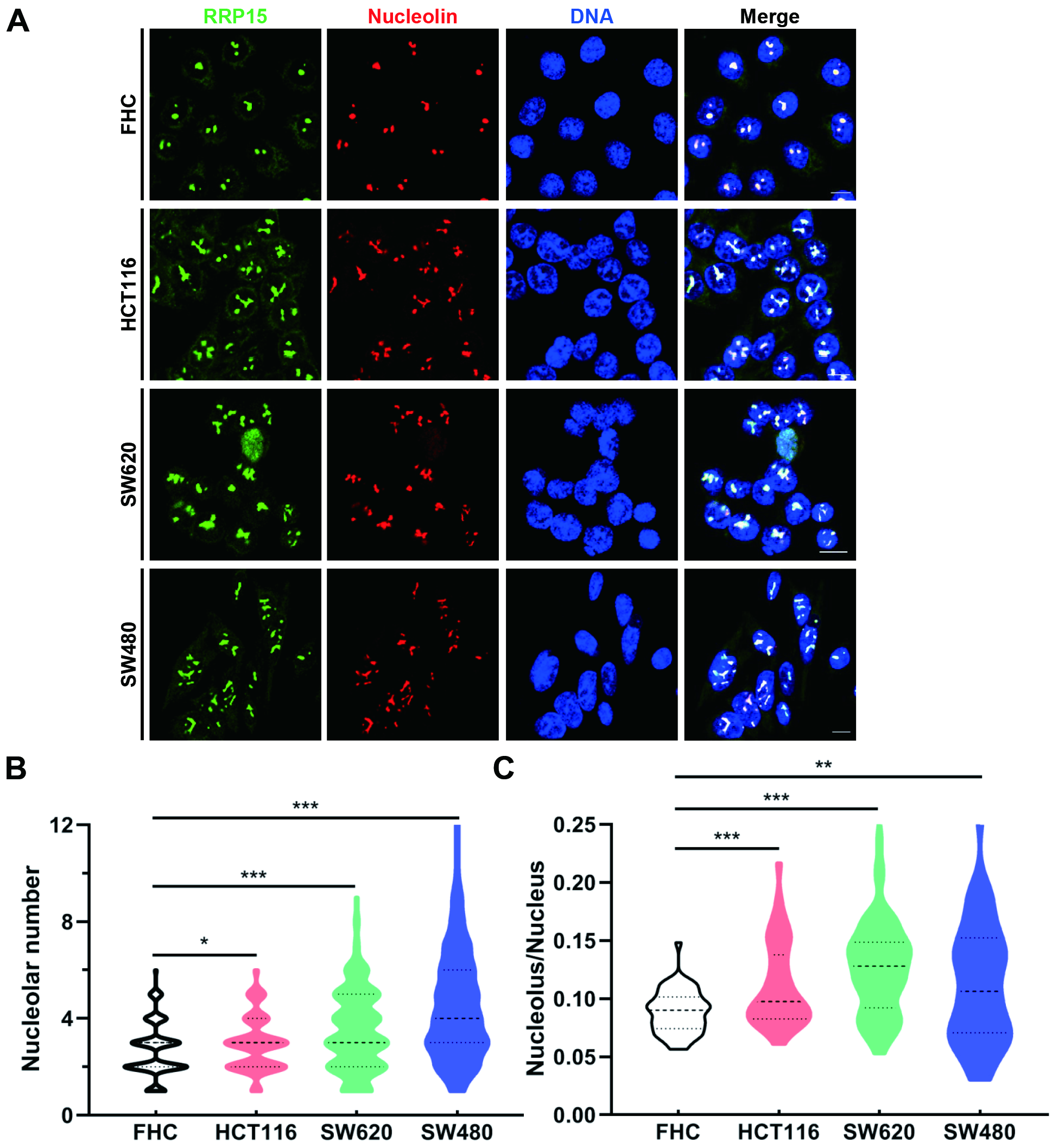


**Figure S2 CRC cells display larger size and increased number of nucleoli.** (A) Normal colon epithelial cells and indicated CEC cells were fixed and immunostained with RRP15 and nucleolin antibodies. DNA was labeled with DAPI. Scale bar: 10 μm. Quantification of the number (B) and size relative to nucleus (C) of nucleoli in each cell (n>50). **P*<0.05, ***P*<0.01 and ****P*<0.001.


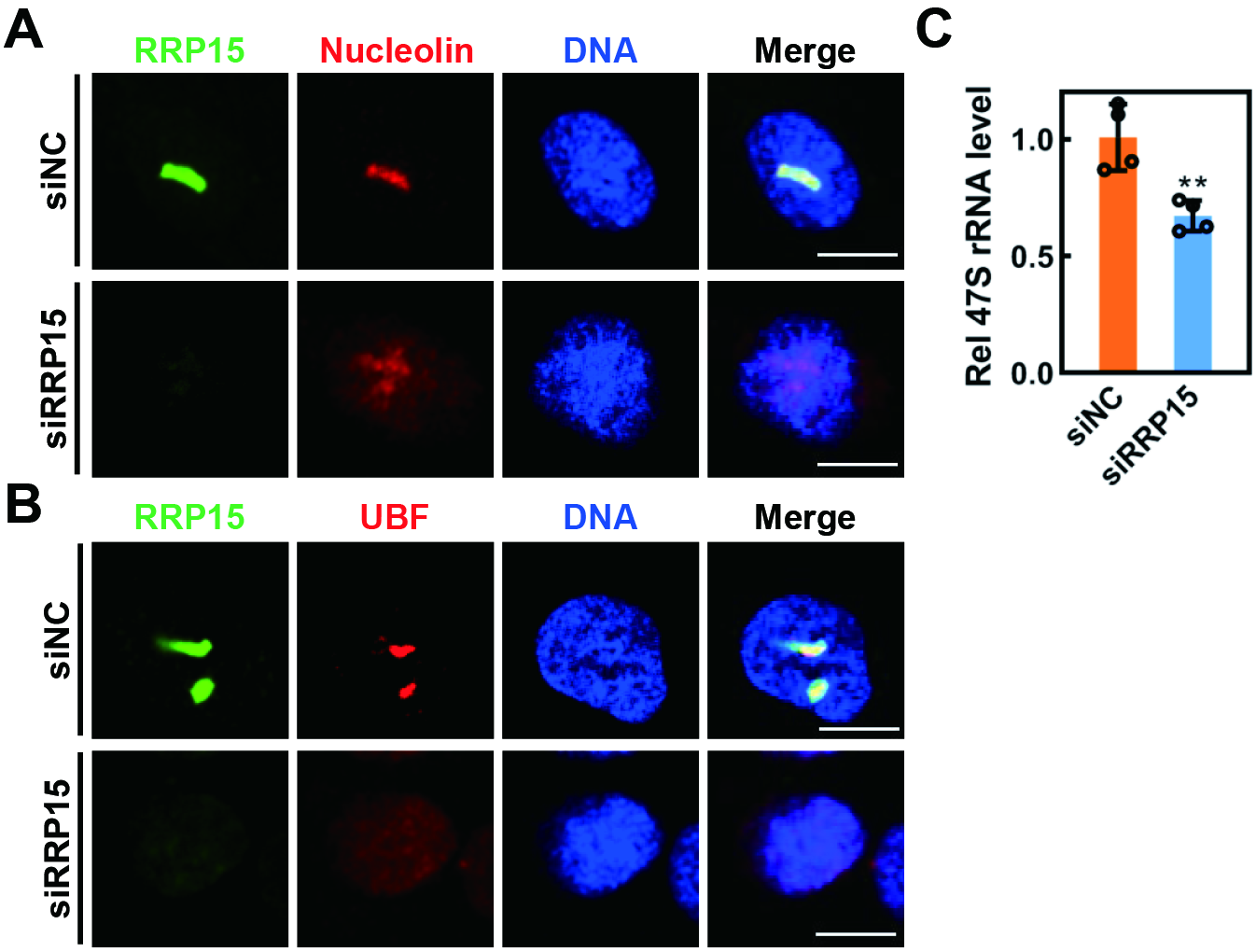


**Figure S3 RRP15 KD causes a dispersed localization of nucleolar proteins and decease of 47S rRNA.** (A-B) RRP15 KD and control cells were fixed and stained with indicated antibodies. DNA was labeled with DAPI. Scale bar, 10 μm. (C) qRT-PCR analysis of 47S rRNA expression in HCT116 cells transfected with siNC and siRRP15. The experiments were repeated three times independently. Data are shown as mean ± standard deviations. ***P*<0.01.


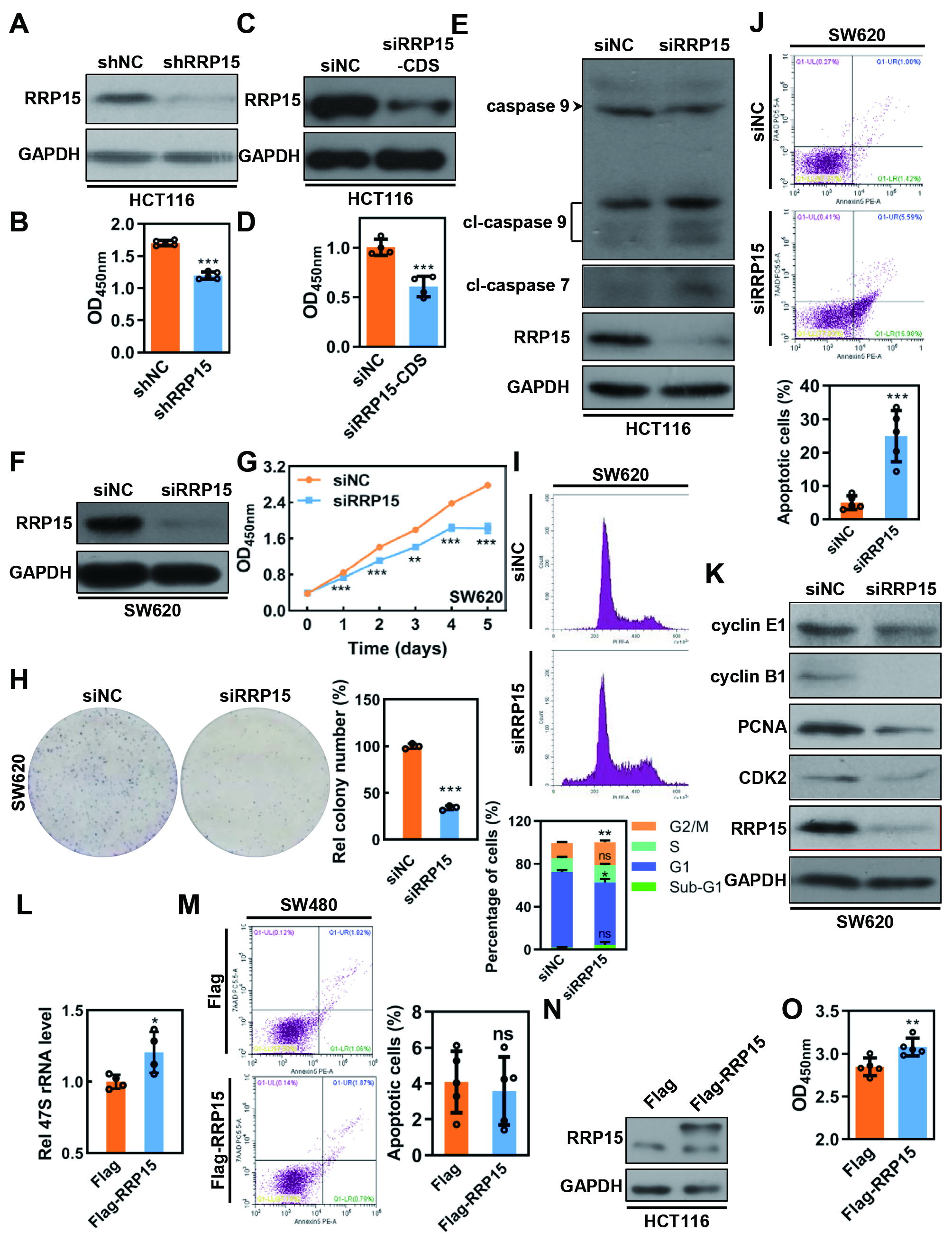


**Figure S4 RRP15 regulates cells proliferation, cell cycle progression and apoptosis in CRC cells.** (A) Western blot analysis for RRP15 expression in HCT116 cells with stably RRP15 KD and controls. (B) Cell proliferation of HCT116 cells with stably RRP15 KD and controls was examined by CCK8 assay. (C) Western blot analysis for RRP15 expression in HCT116 cells transfected with siNC or siRRP15-CDS. (D) Cell proliferation of HCT116 cells transfected with siNC or siRRP15-CDS was examined by CCK8 assay. (E) Western blot analysis for apoptotic related proteins in HCT116 cells after RRP15 KD. (F) Western blot analysis for RRP15 expression in SW620 cells transfected with siNC or siRRP15. (G) Proliferation curve of SW620 cells transfected with siNC or siRRP15 for indicated time. (H) Colony formation assay to detect the cell proliferation ability of SW620 cells after RRP15 KD. The colony numbers were counted using Image J software. Cell cycle profile (I) and apoptosis (J) of SW620 cells after transfected with siNC or siRRP15. (K) Western blot analysis of the cell proliferative and cell cycle related proteins of SW620 cells after indicated siRNA transfection. (L) qRT-PCR analysis of 47S rRNA expression in SW480 cells with stably RRP15 OE and controls. (M) Apoptosis assay of SW480 cells with stably RRP15 OE and controls. (N) Western blot for RRP15 expression in HCT116 cells transfected with pCDH-RRP15 or vector. (O) Cell proliferation of HCT116 cells transfected with pCDH-RRP15 or vector was examined by CCK8 assay. The experiments were repeated three times independently. Data are shown as mean ± standard deviations. ns: no significant; **P*<0.05, ***P*<0.01 and ****P*<0.001.


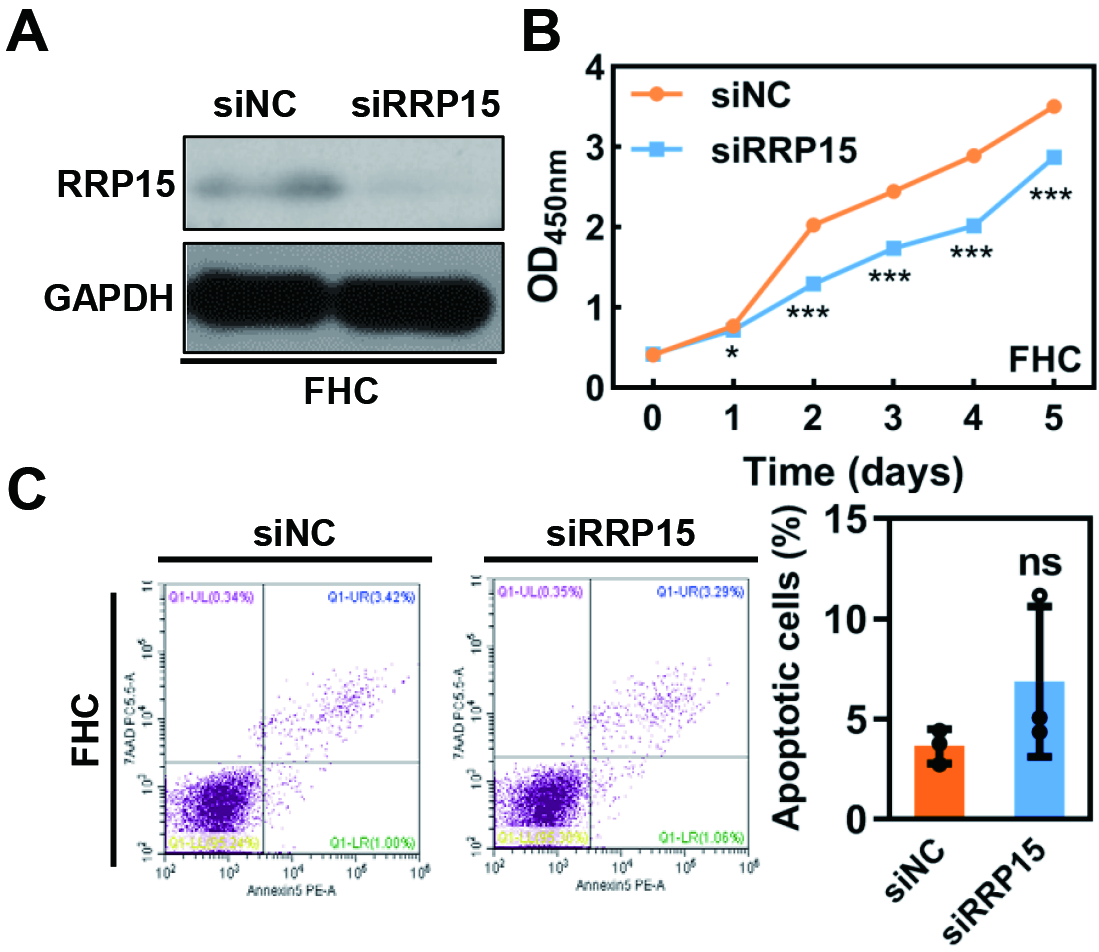


**Figure S5 RRP15 KD induces a decrease of proliferation but not apoptosis in FHC cells.** (A) Western blot analysis for RRP15 expression in FHC cells transfected with siNC or siRRP15. (B) Proliferation curve of FHC cells transfected with siNC or siRRP15 for indicated time. (C) Apoptosis assay of FHC cells transfected with siNC or siRRP15. The experiments were repeated three times independently. Data are shown as mean ± standard deviations. ns: no significant; **P*<0.05 and ****P*<0.001.

**
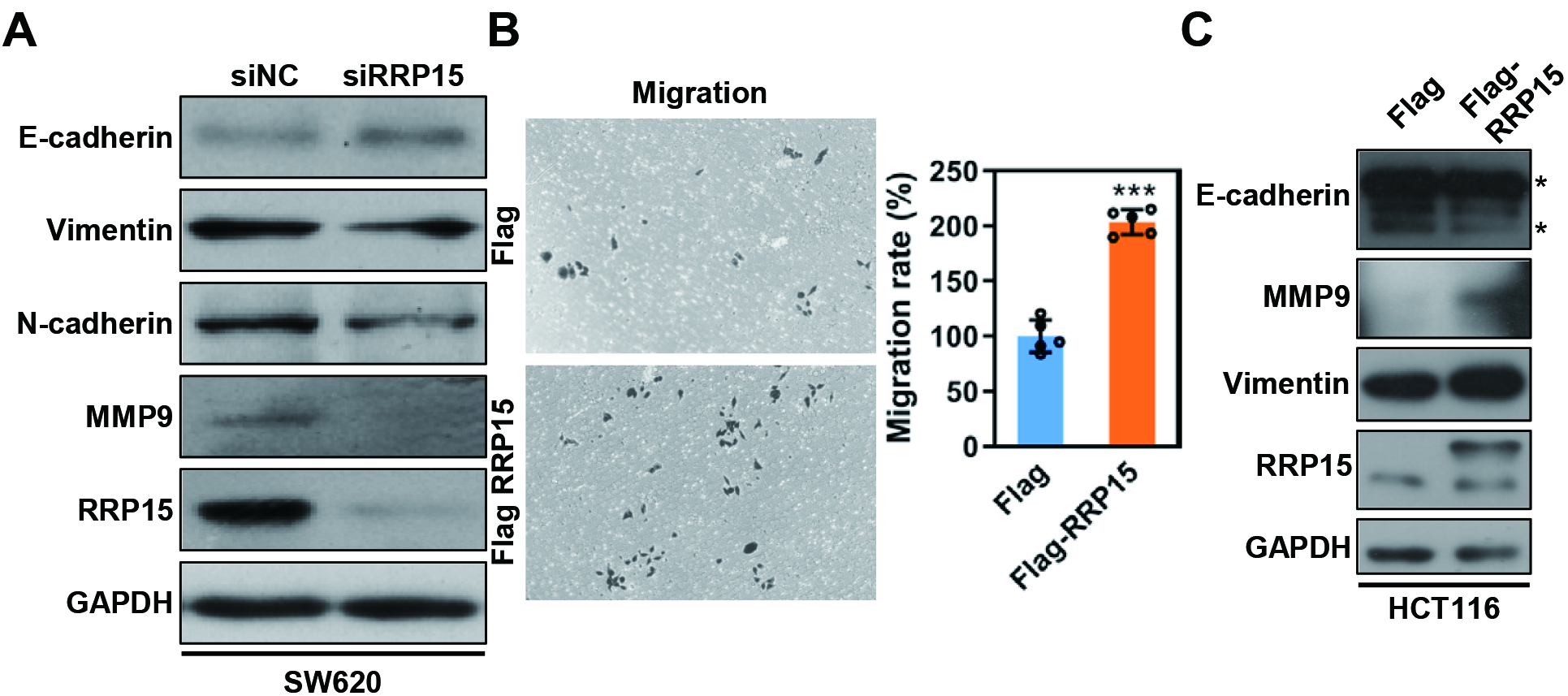
**

**Figure S6 RRP15 expression is positively correlated with metastatic capacity of CRC cells.** (A) Western blot for EMT related proteins levels in SW620 cells transfected with siNC or siRRP15. (B) Transwell migration assay of HCT116 cells transfected with pCDH-RRP15 or vector. All experiments were performed at least three times. Data are shown as mean ± standard deviations. ***P< 0.001. (C) Western blot for EMT related proteins levels in HCT116 cells transfected with pCDH-RRP15 or vector. *: non-specific band.

**
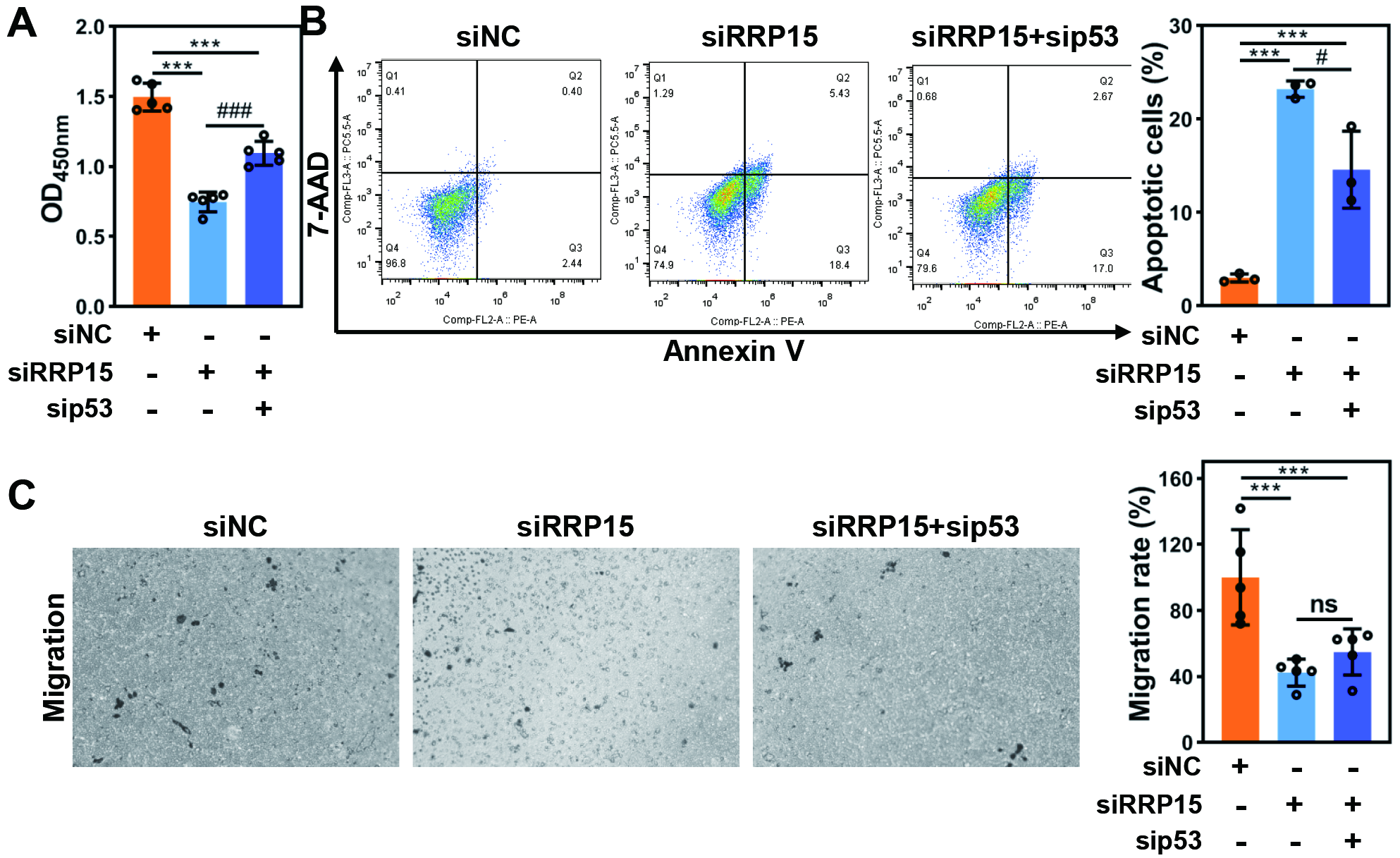
**

**Figure S7 The effects of p53 on RRP15-mediated proliferative and migratory inhibition.** (A) Cell proliferation of HCT116 cells transfected with indicated siRNA was examined by CCK8 assay. (B) Apoptosis assay of HCT116 cells transfected with indicated siRNA. (C) Transwell migration assay of HCT116 cells transfected with indicated siRNA. All experiments were performed at least three times. Data are shown as mean ± standard deviations. ns: no significant; ****P*< 0.001; # *P*<0.05 and ###*P*<0.001.

**
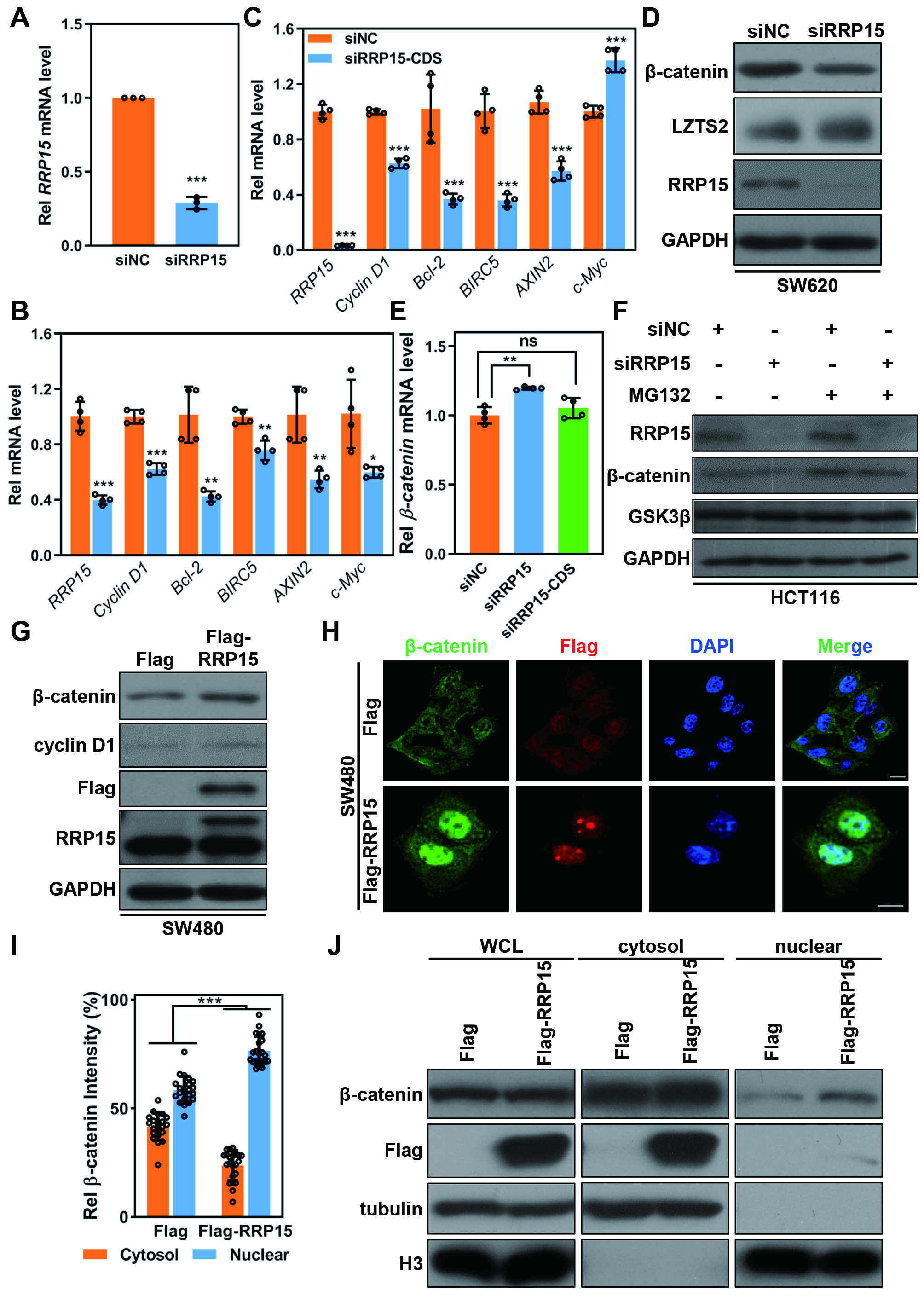
**

**Figure S8 RRP15 regulates Wnt/β-catenin signal pathway in CRC cells.** (A) RNAseq data analysis of RRP15 expression in HCT116 cells transfected with siNC or siRRP15. (B) qRT-PCR analysis of indicated Wnt/β-catenin targets in siRRP15 or siNC transfected SW620 cells. (C) qRT-PCR analysis of indicated Wnt/β-catenin targets in siRRP15-CDS or siNC transfected HCT116 cells. (D) Western blot analysis for determining β-catenin and LZTS2 expression after RRP15 KD in SW620 cells. (E) qRT-PCR analysis of *β-catenin* mRNA after RRP15 KD in HCT116 cells. (F) Western blot analysis for β-catenin in siRRP15 or siNC transfected HCT116 cells after indicated treatment. (G) Western blot for β-catenin and cyclin D1 in SW480 cells with stably RRP15 OE. (H-I) Representative immunostaining images of RRP15 and β-catenin in SW480 cells with stably RRP15 OE. DNA was labeled with DAPI. Scale bar, 10 μm. Relative β-catenin staining intensity in nucleus was counted and data were obtained from at least 20 cells. (J) Nuclear or cytoplasmic lysates from HCT116 cells with stably RRP15 KD or controls were extracted and subjected to western blot analysis with indicated antibodies. The experiments were performed at least three times, and data are shown as mean ± standard deviations. ****P*<0.001; ns: not significant.


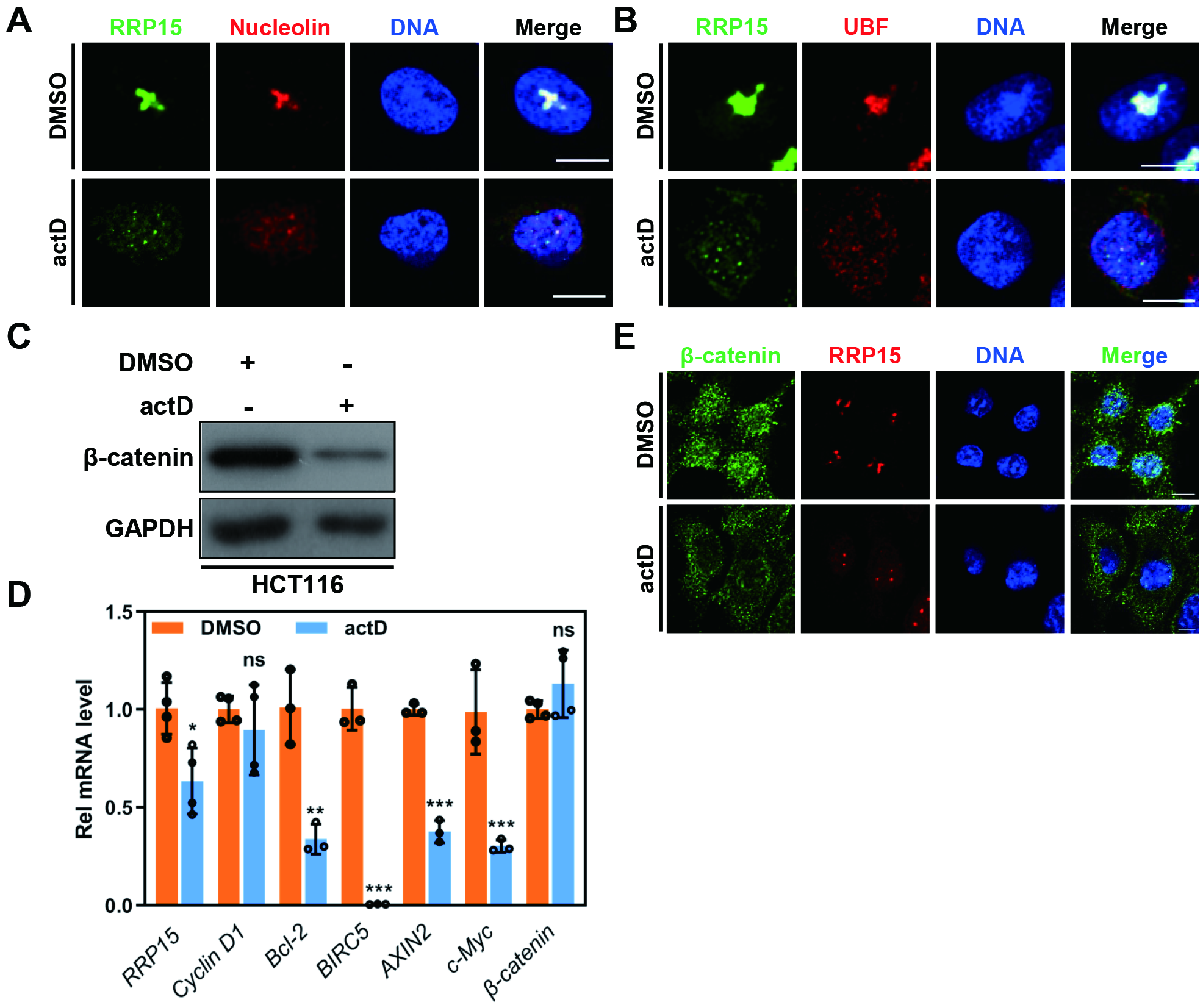


**Figure S9 IRBC induced by actinomycin D (actD) treatment inhibits Wnt/β-catenin signaling.** Representative immunostaining images of nucleolin (A) or UBF (B) in HCT116 cells treated with actD. DNA was labeled with DAPI. Scale bar, 10 μm. (C) Western blot for β-catenin expression in HCT116 cells treated with actD. (D) qRT-PCR analysis of indicated Wnt/β-catenin targets in HCT116 cells treated with actD. (E) Representative immunostaining images of β-catenin and RRP15 in HCT116 cells treated with actD. DNA was labeled with DAPI. Scale bar, 10 μm. The experiments were performed at least three times, and data are shown as mean ± standard deviations. ns: no significant, **P*< 0.05, ***P*< 0.01 and ****P*< 0.001.


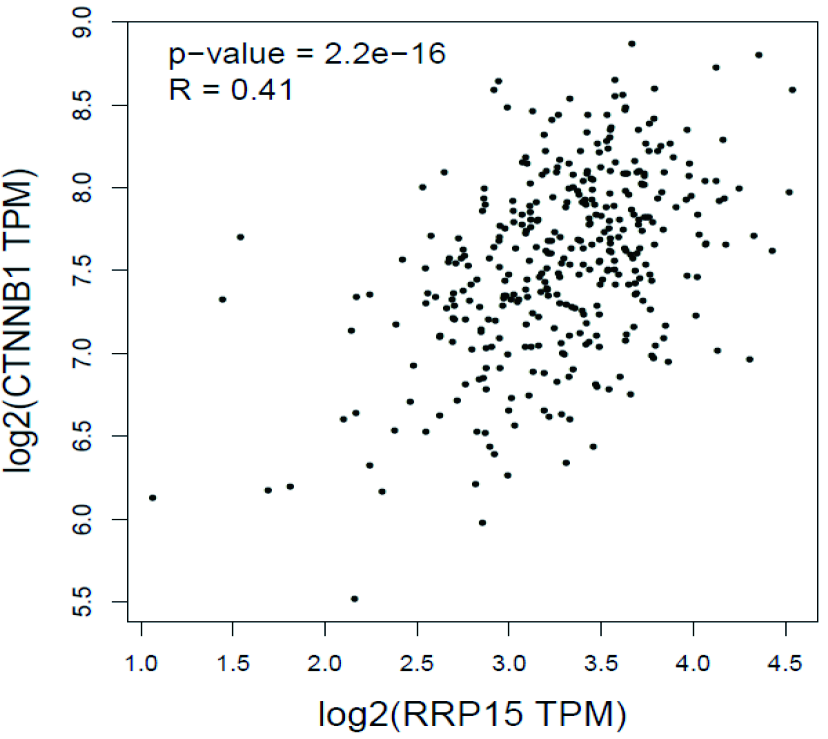


**Figure S10 The expression of *RRP15* and *CTNNB1* genes in CRC tumor tissues are positively correlated** (R=0.41, *P*=2.2e-16).


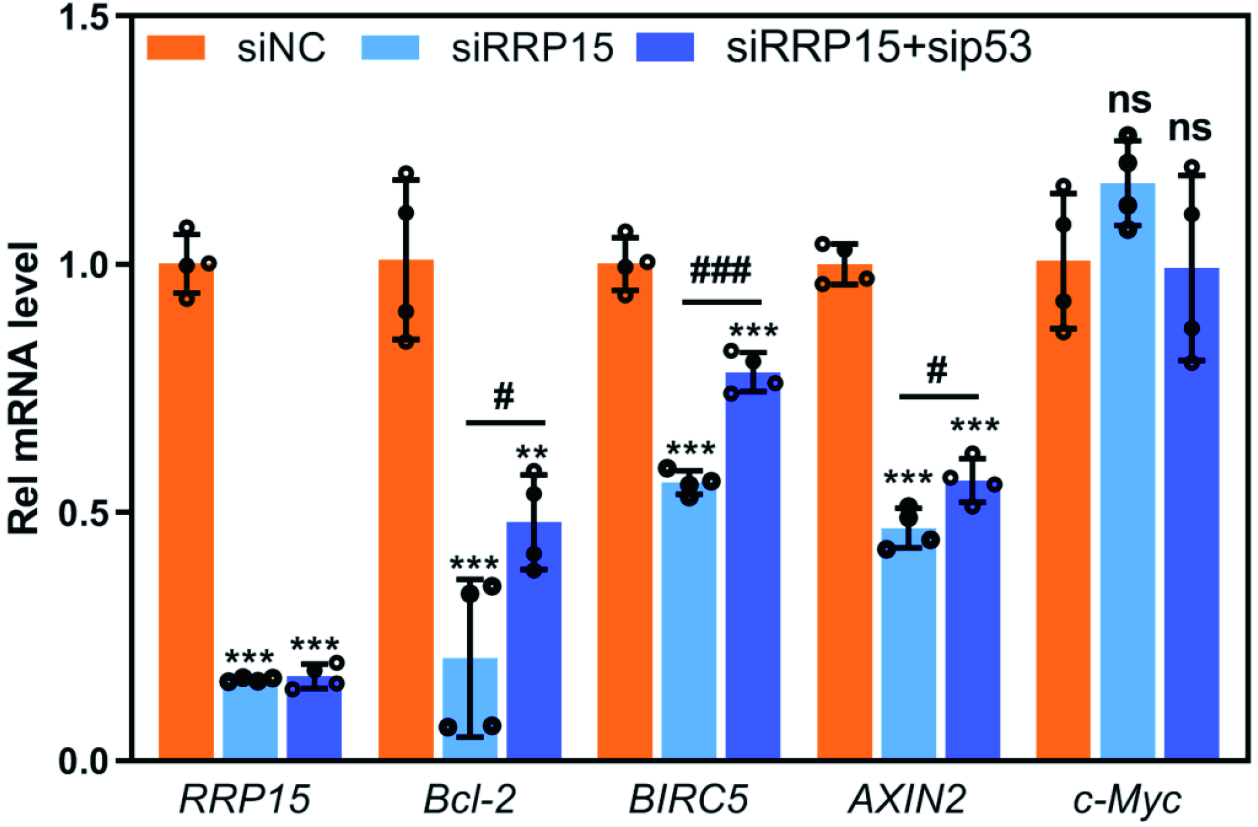


**Figure S11 Co-knockdown of p53 partially rescues the inhibition of RRP15 KD on Wnt/β-catenin signaling downstream targets.** qRT-PCR analysis of indicated Wnt/β-catenin targets in HCT116 cells transfected with indicated siRNA. The experiments were performed at least three times, and data are shown as mean ± standard deviations. ns: no significant, **P*< 0.05, ***P*< 0.01 and ****P*< 0.001.


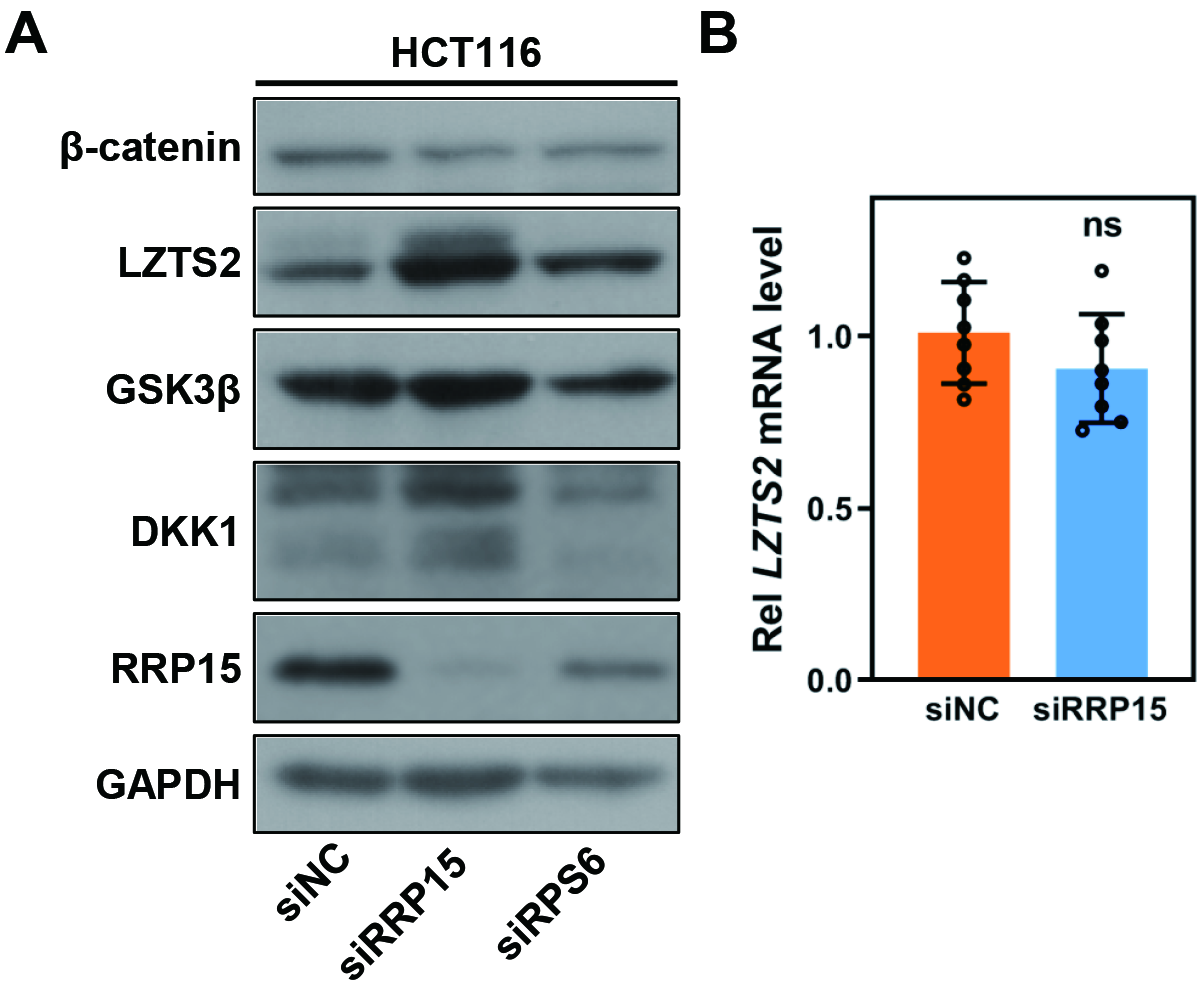


**Figure S12 The effect of IRBC on expression of TOP mRNA in HCT116 cells.** (A) Western blot analysis for several Wnt related genes with TOP structure in HCT116 cells transfected with indicated siRNA. (B) qRT-PCR analysis of *LZTS2* mRNA in HCT116 cells transfected with siNC or siRRP15. The experiments were performed at least three times, and data are shown as mean ± standard deviations. ns: no significant.

**
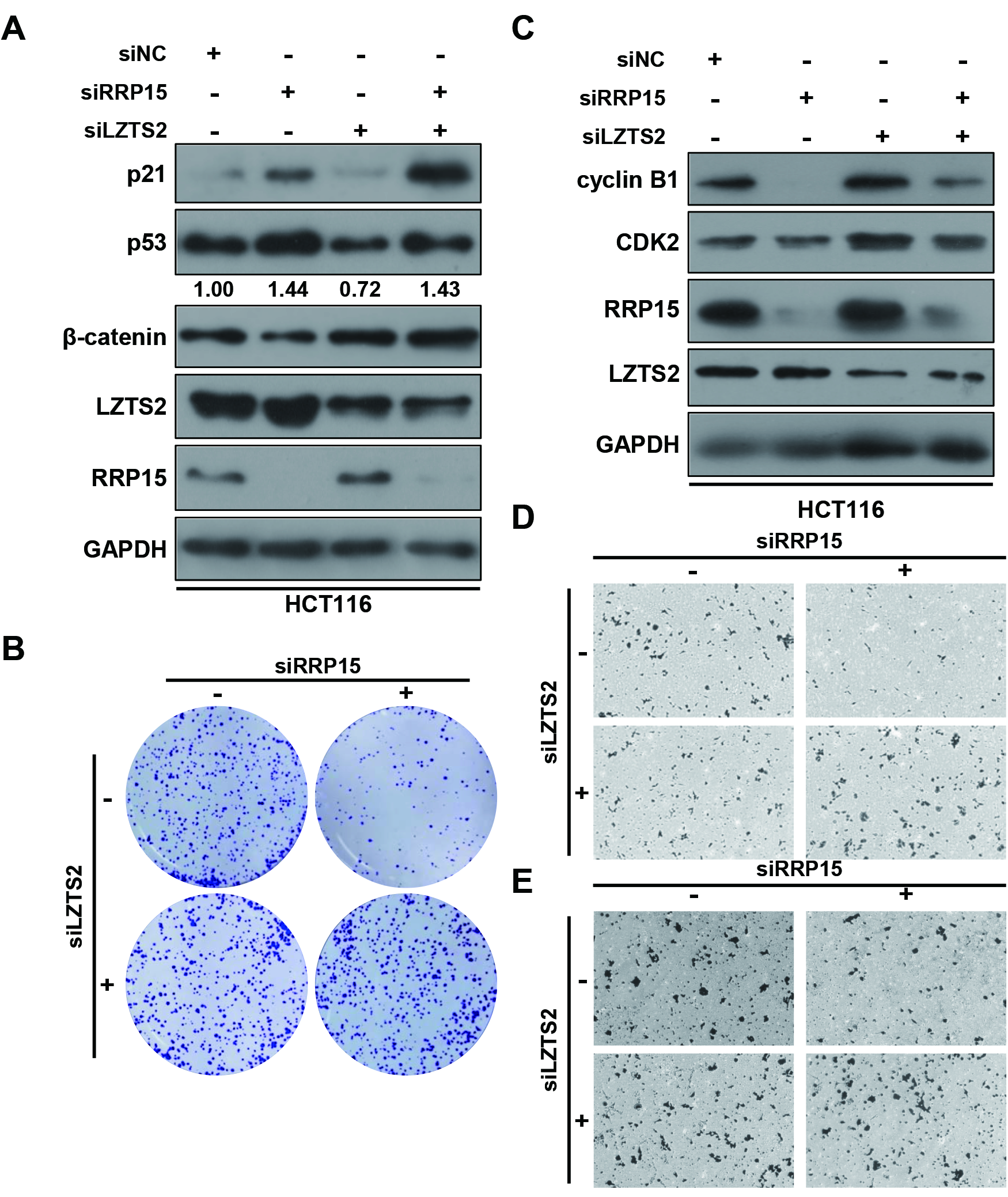
Figure S13 LZTS2 KD reverses the effects induced by RRP15 KD in HCT116 cells.** (A) Western blot analysis for indicated proteins in HCT116 cells after transfected with indicated siRNA. (B) Colony formation assay to detect the cell proliferation ability of HCT116 cells transfected with indicated siRNA. (C) Western blot analysis for cell cycle related proteins in HCT116 cells after transfected with indicated siRNA. Transwell migration (D)and invasion assay (E) of HCT116 cells transfected with indicated siRNA.
