# Supplementary material for: RRP15 deficiency induces ribosome stress to inhibit colorectal cancer proliferation and metastasis via LZTS2-mediated β-catenin suppression
Source: Cell Death Dis. 2023 Feb 7;14(2):89. doi: 10.1038/s41419-023-05578-6 (PMC9905588; doi:10.1038/s41419-023-05578-6)
Supplement: Supplementary file 2 — Original Data File [file 41419_2023_5578_MOESM2_ESM.docx]

**Figure 1E:**

GAPDH RRP15







**Figure 2A:**





**Figure 2F:**

p53: p21







cyclin E: cyclin B1:


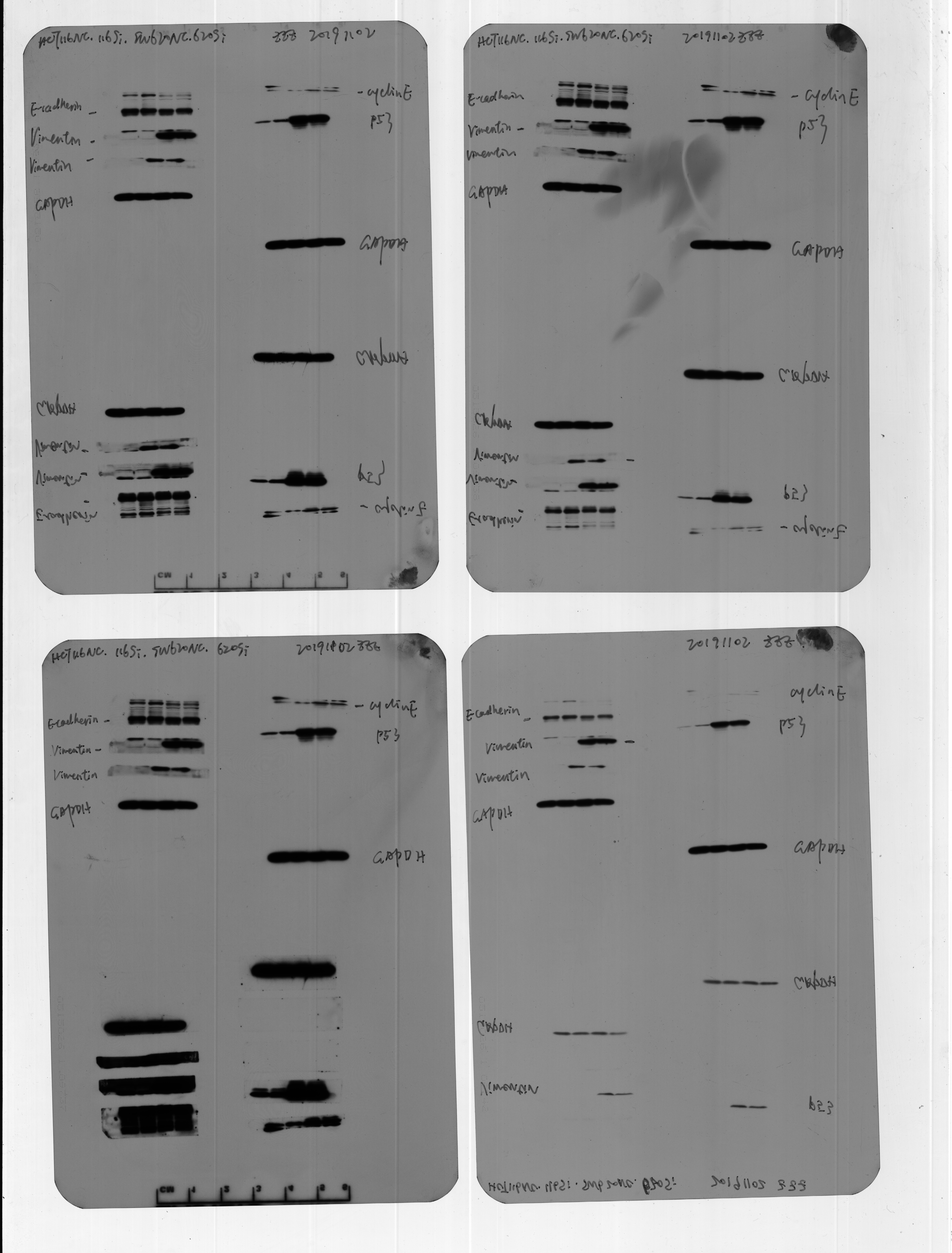

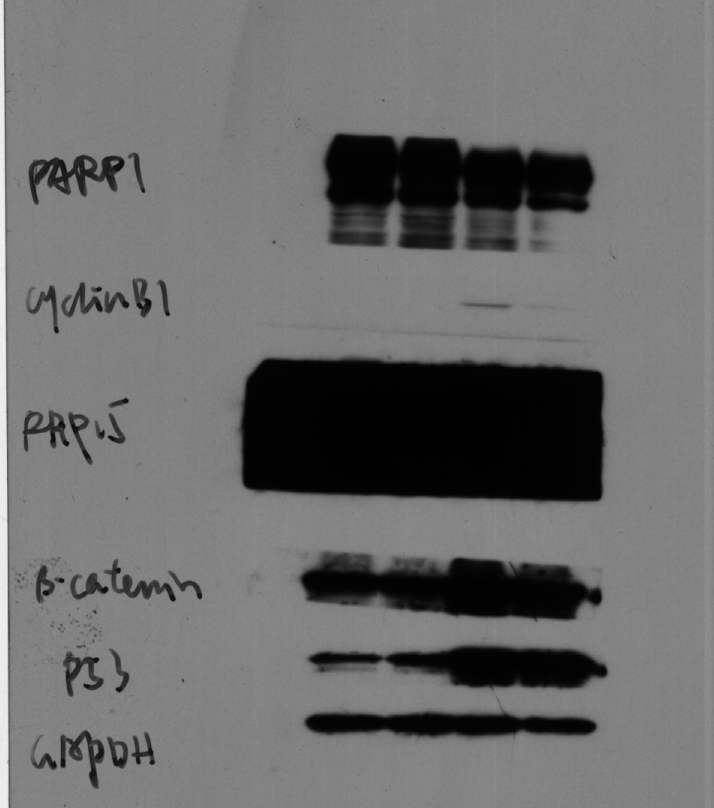


CDK2 and PCNA:





RRP15: GAPDH:


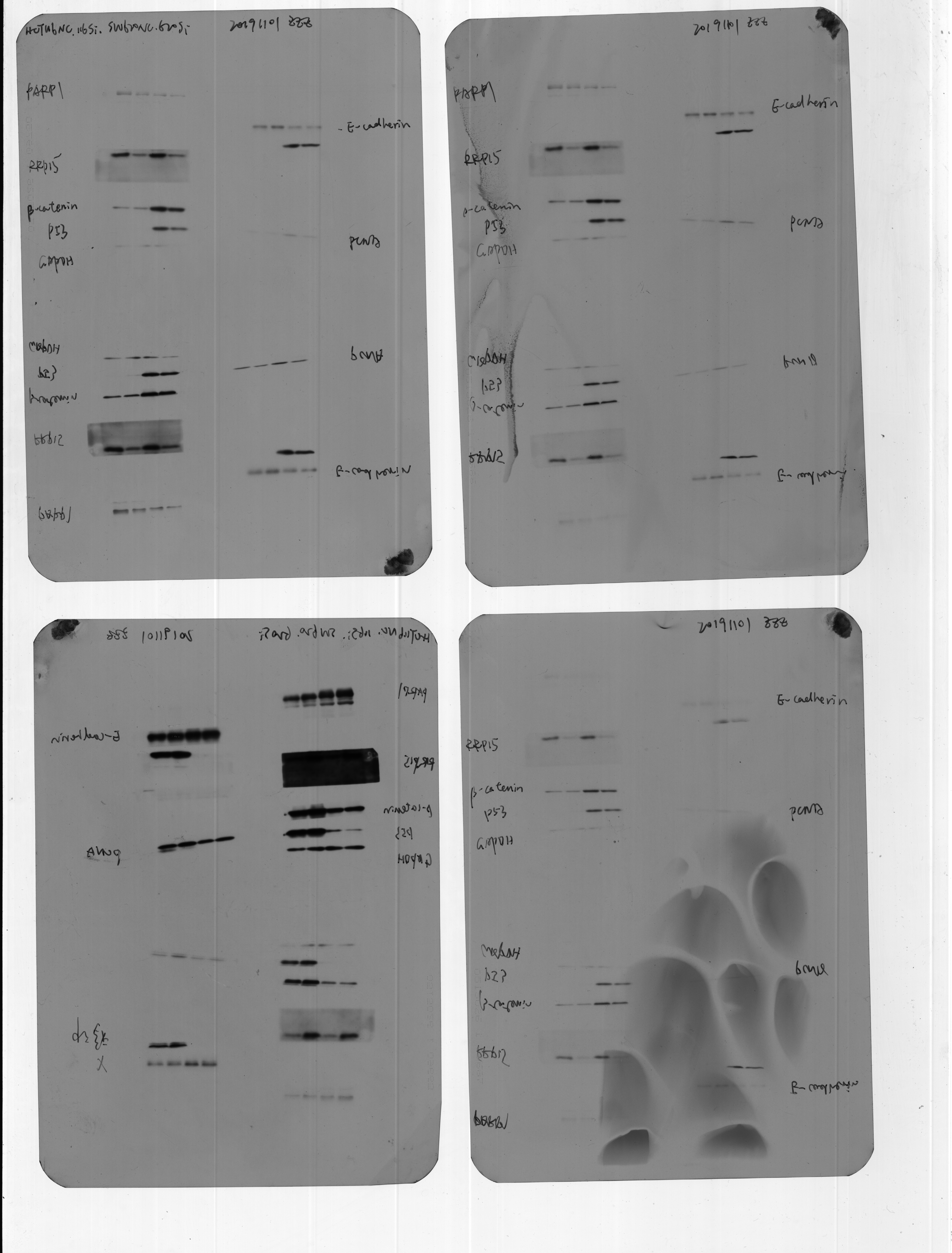


**Figure 2G:**

Flag: RRP15:


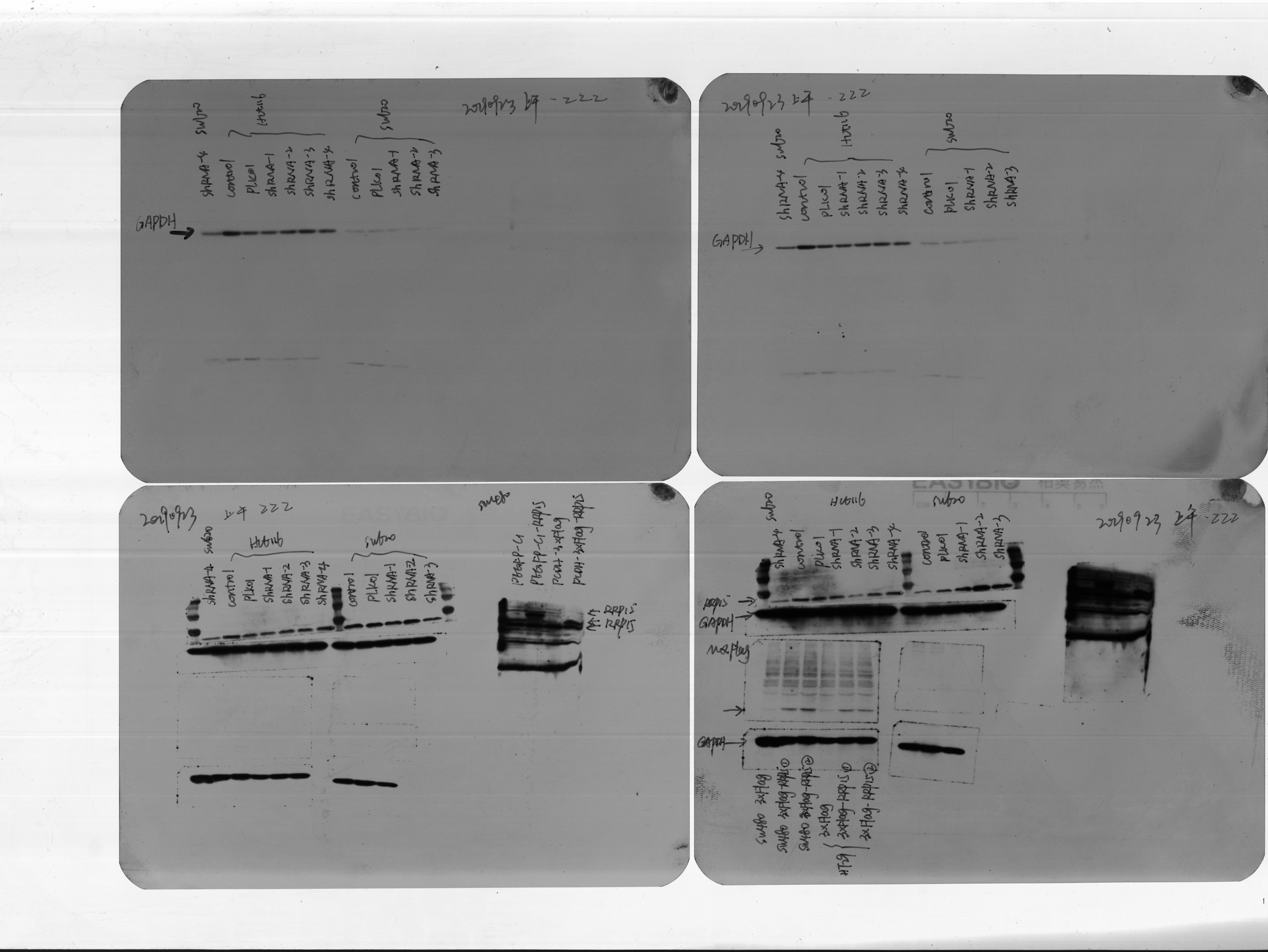




GAPDH:





**Figure 2K:**

p53: p21:




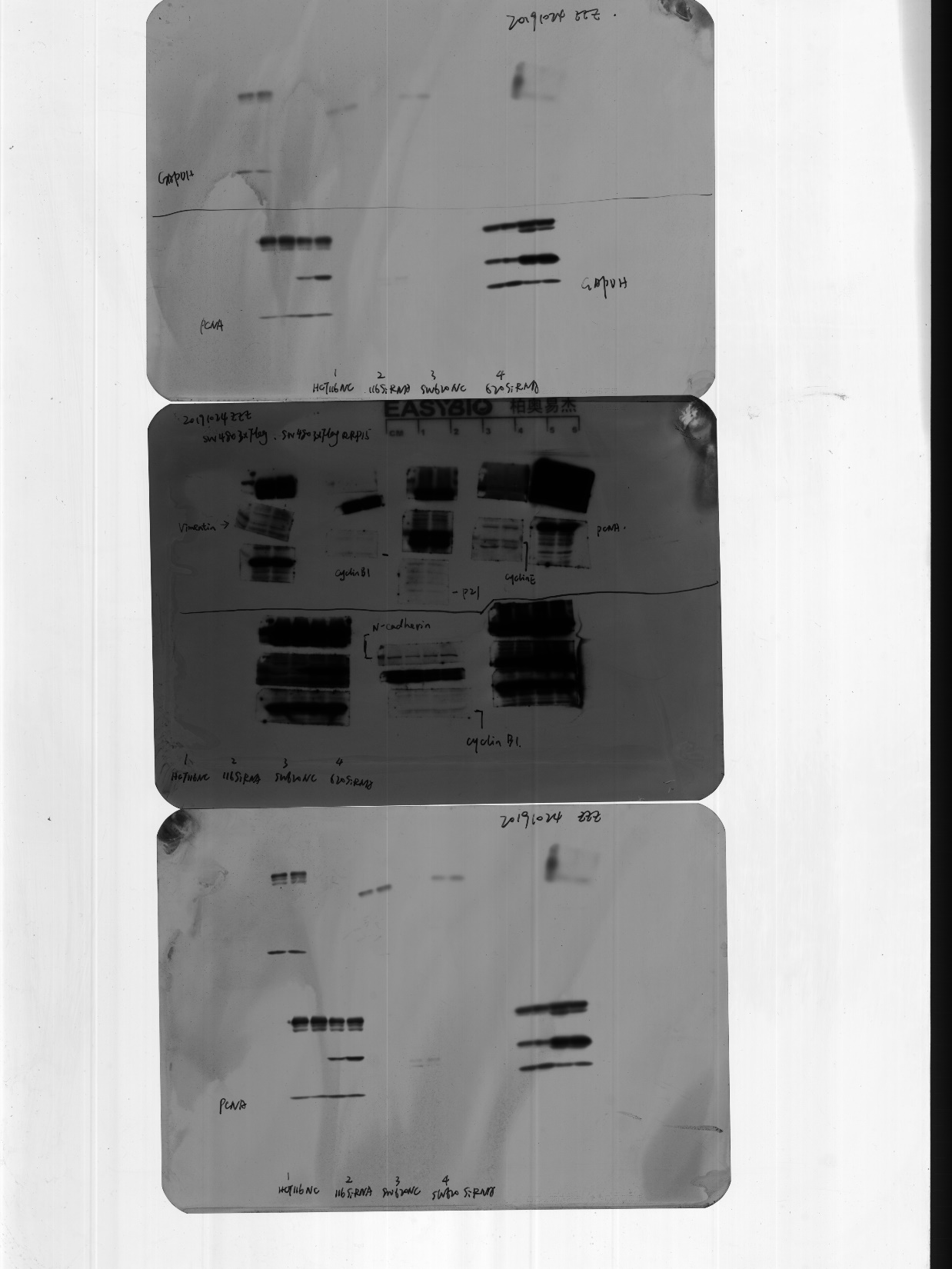


cyclin E1: cyclin B1:


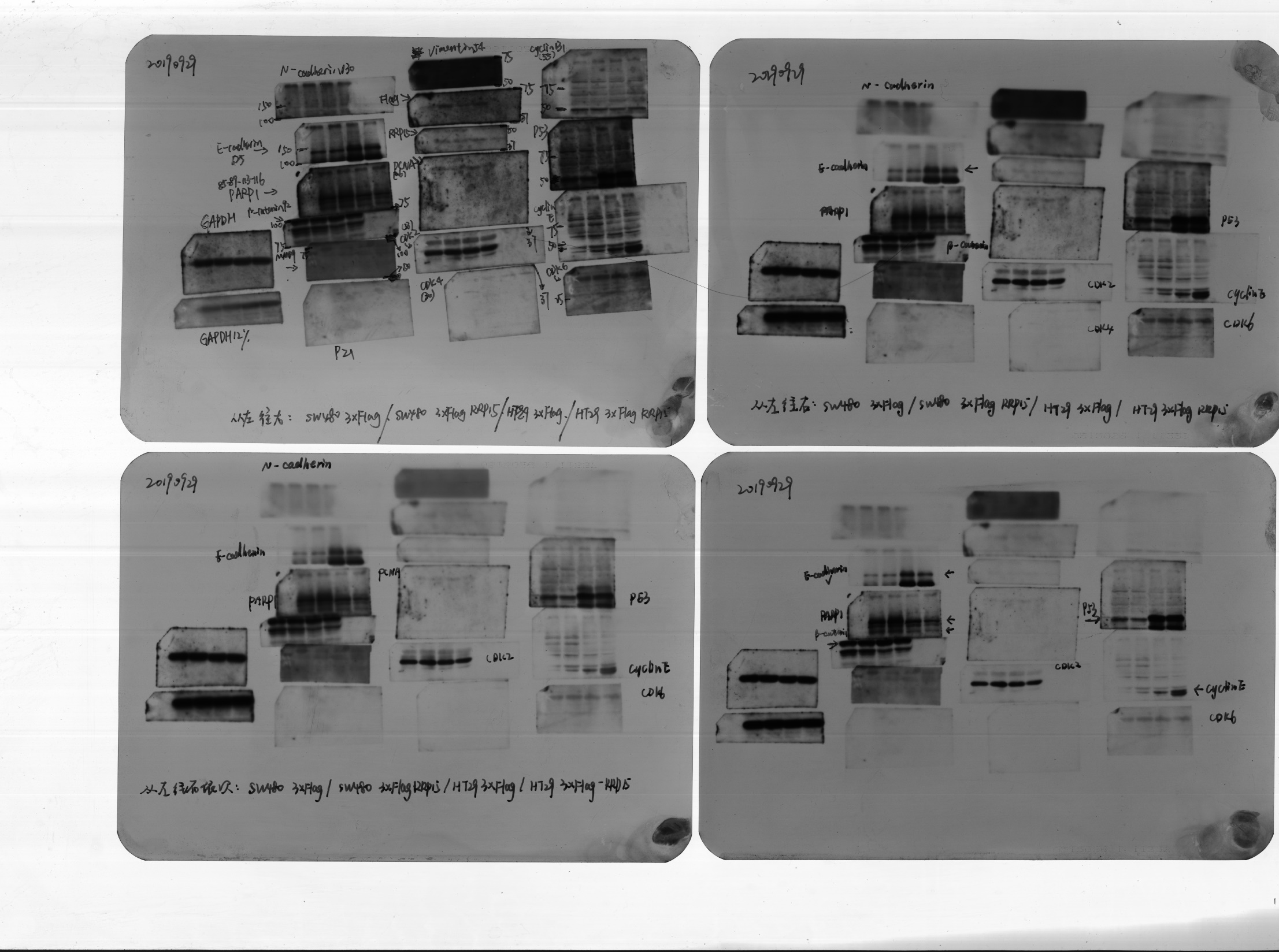




PCNA: CDK2:







Flag, RRP15 and GAPDH:







**Figure 3C:**

E-cadherin: Vimentin:


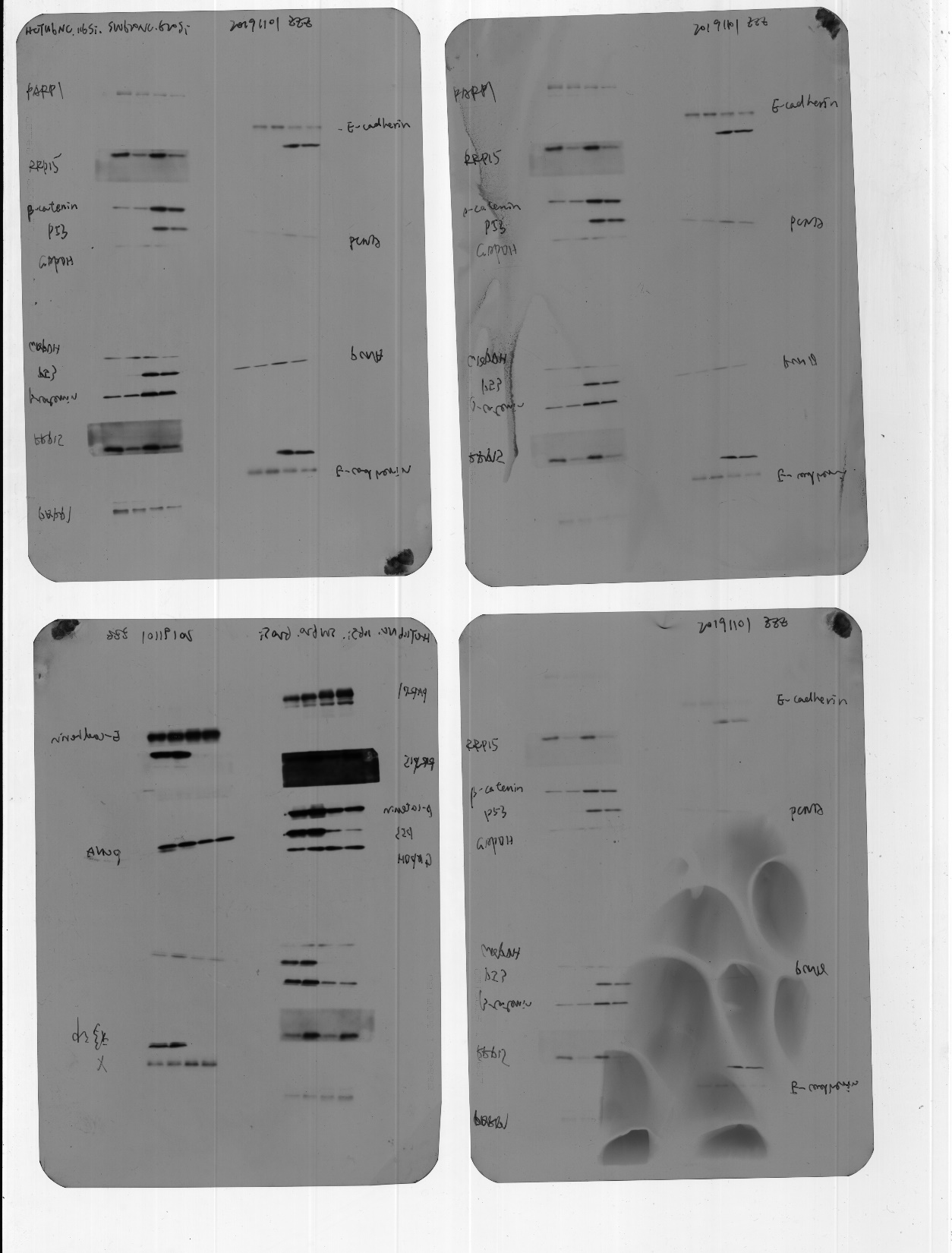




N-cadherin: MMP9:


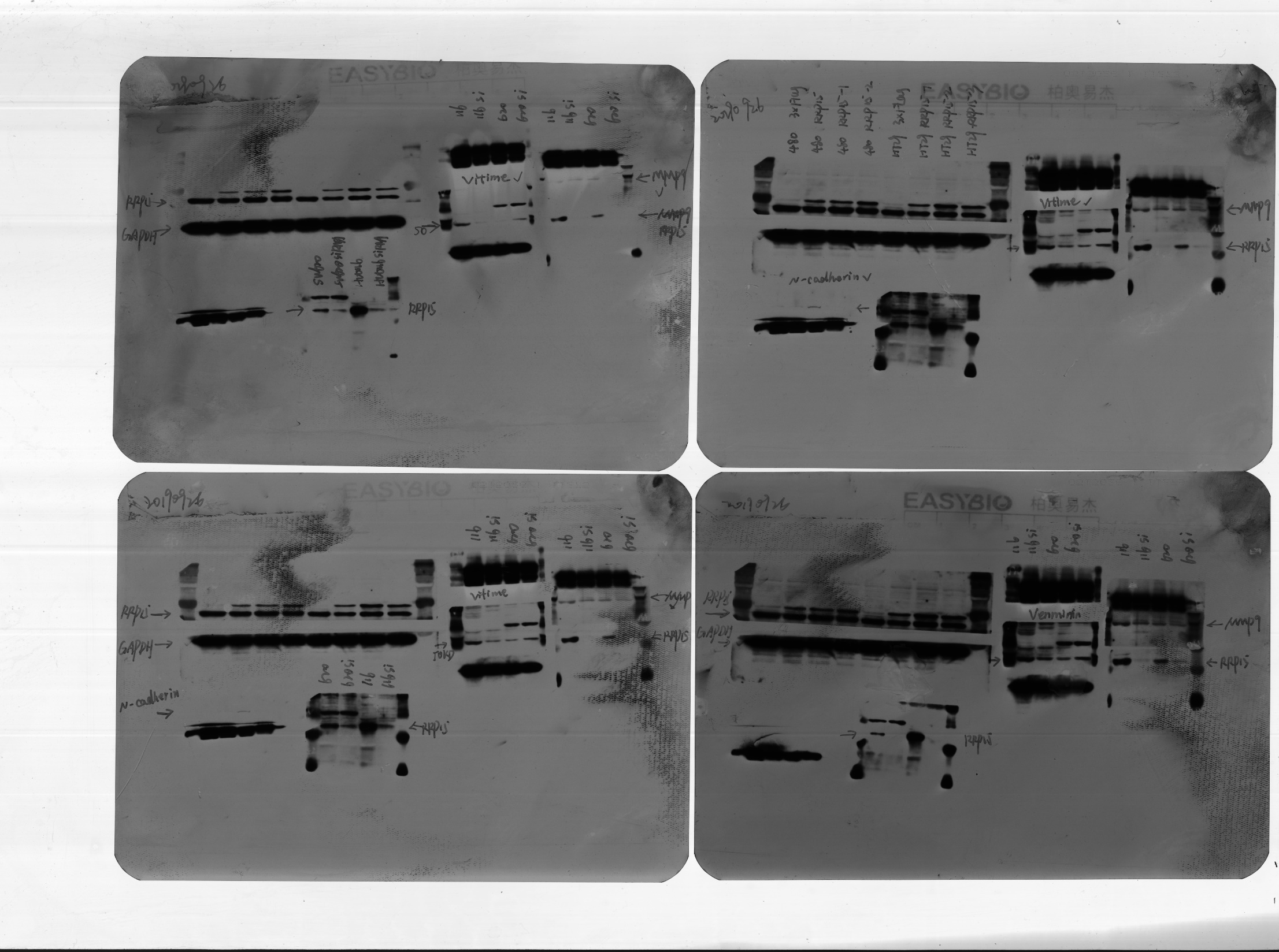




RRP15: GAPDH:




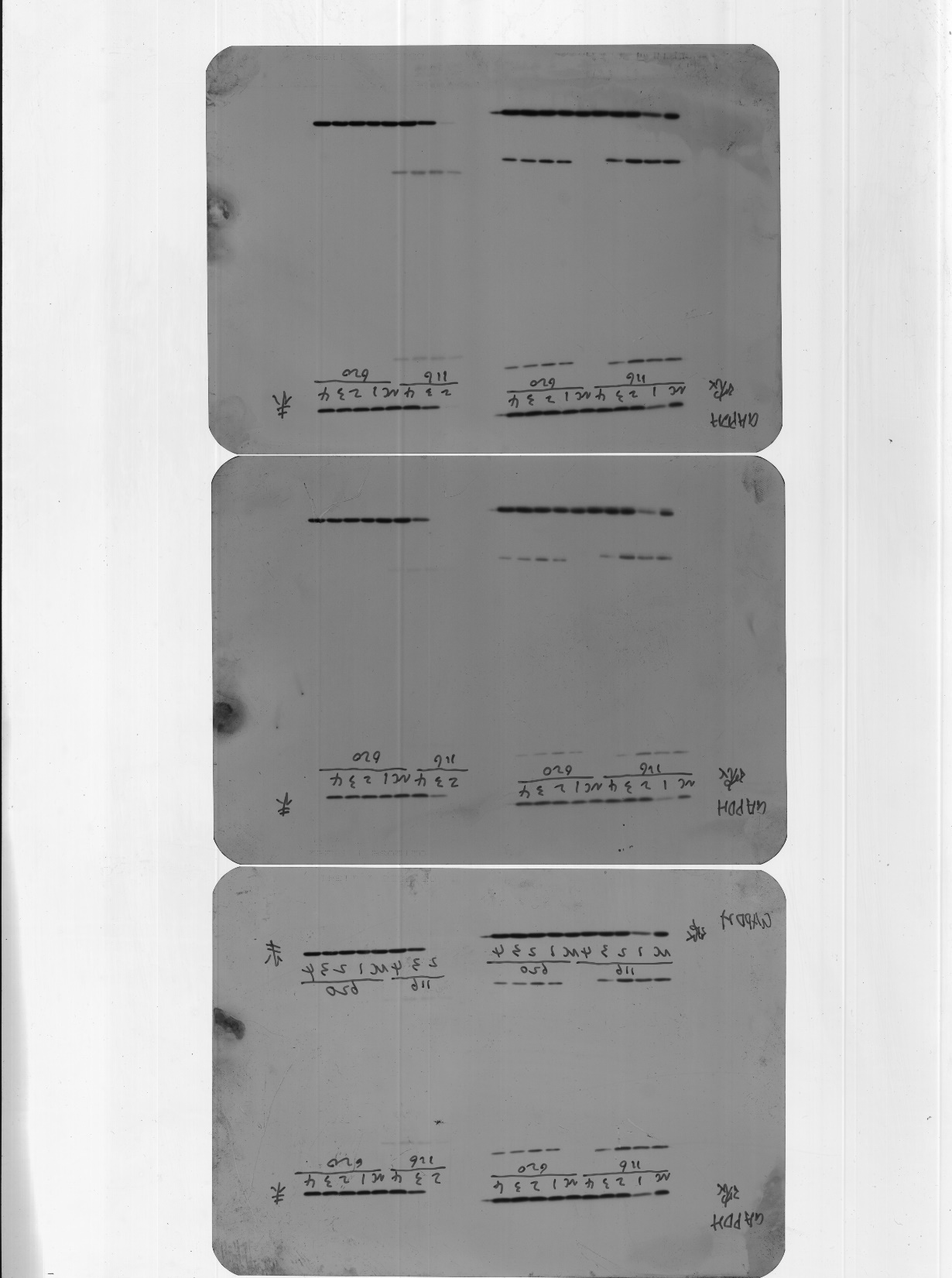


**Figure 3F:**

E-cadherin: Vimentin:







N-cadherin: MMP9:


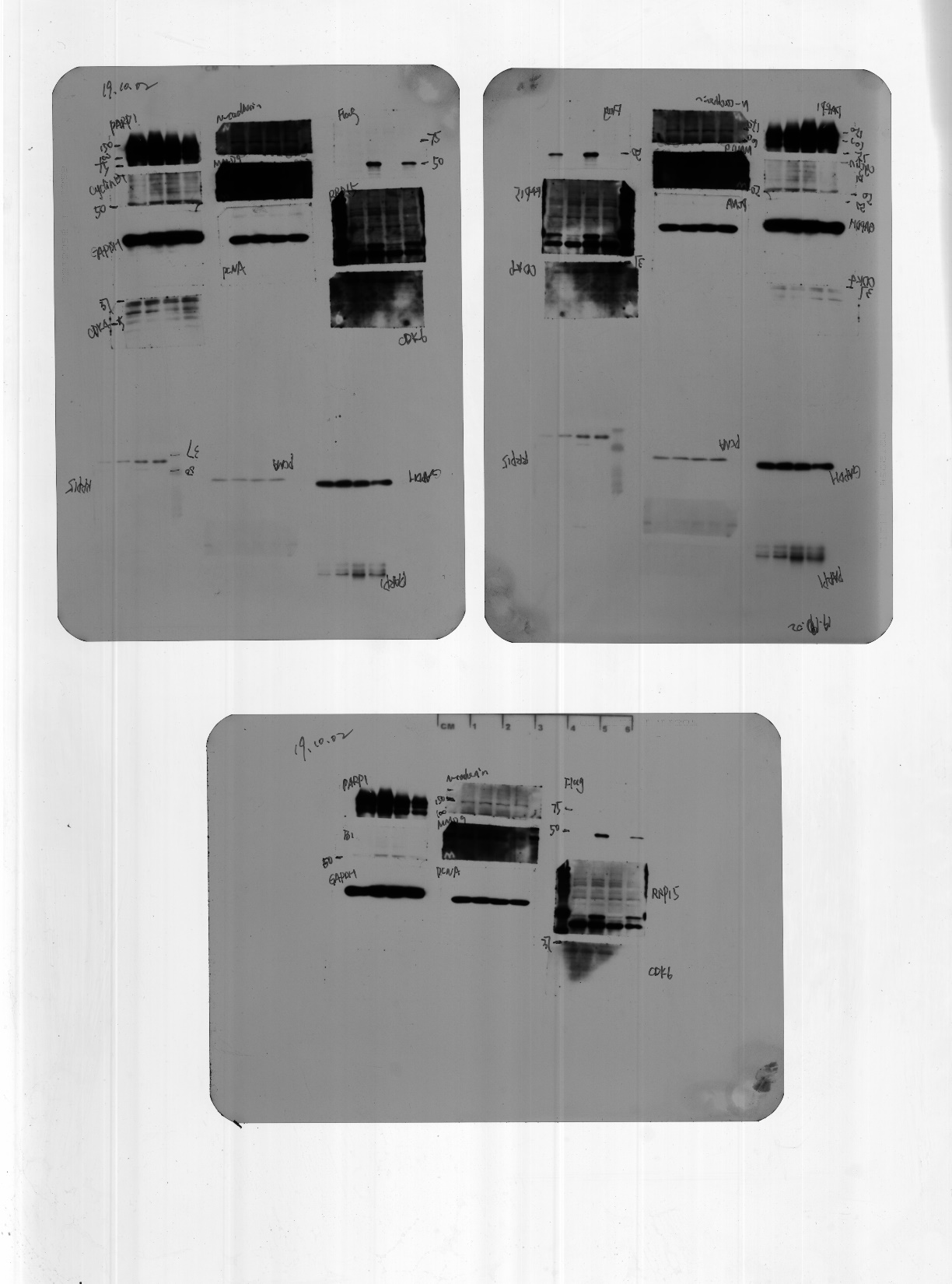

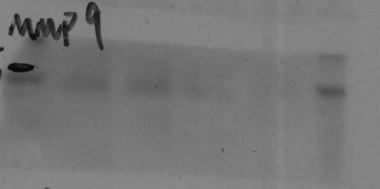


RRP15 and Flag: GAPDH:







**Figure 5C:**

β-catenin:





cyclin D1:





RRP15: GAPDH:


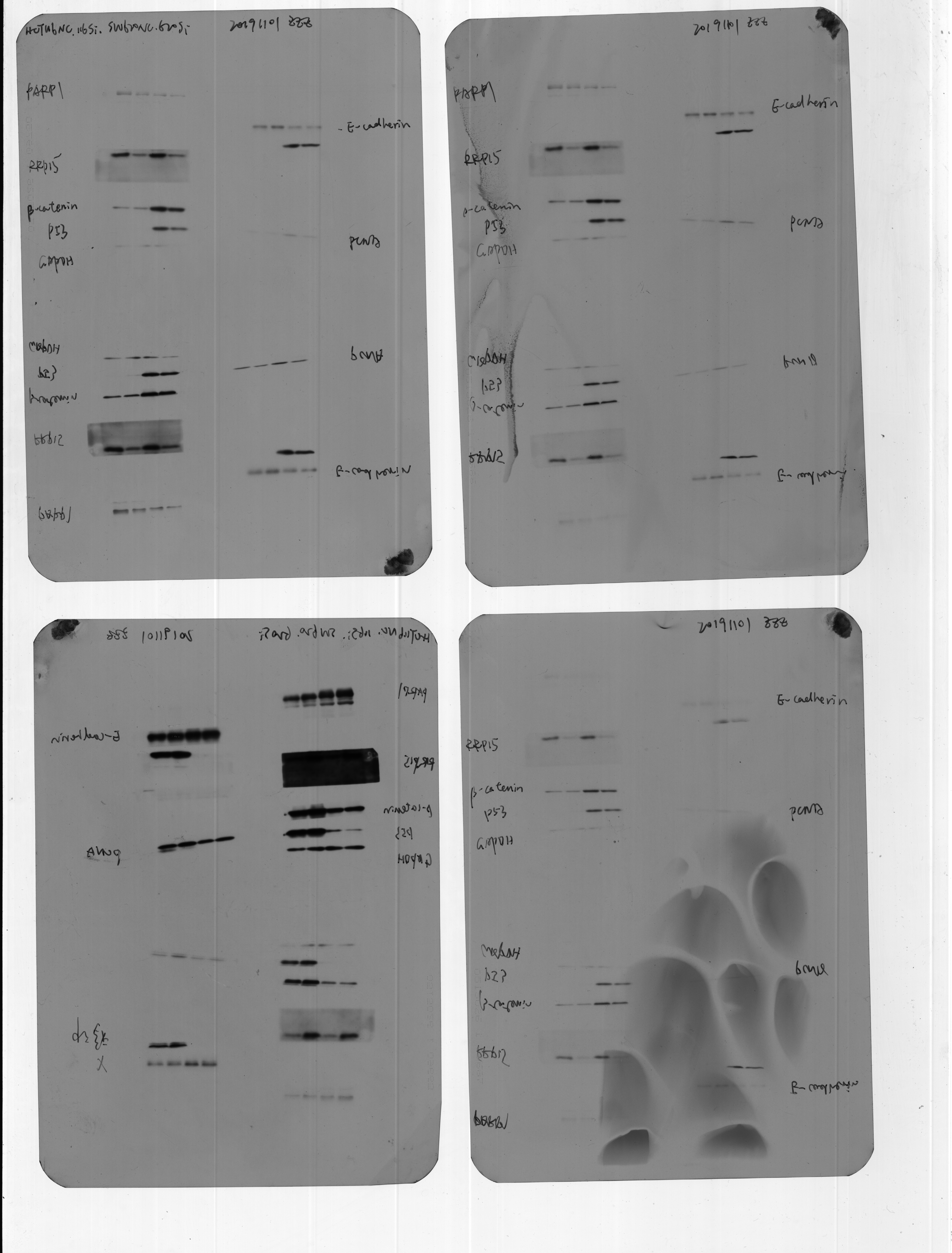


**Figure 5E:**

β-catenin, RRP15 and GAPDH:


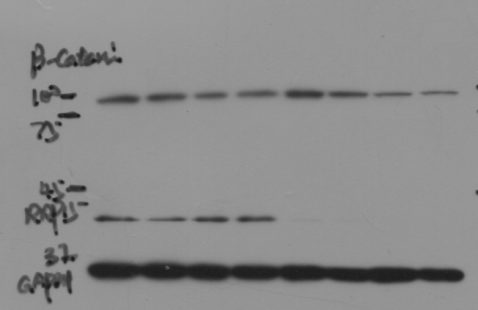


**Figure 5G:**

β-catenin:


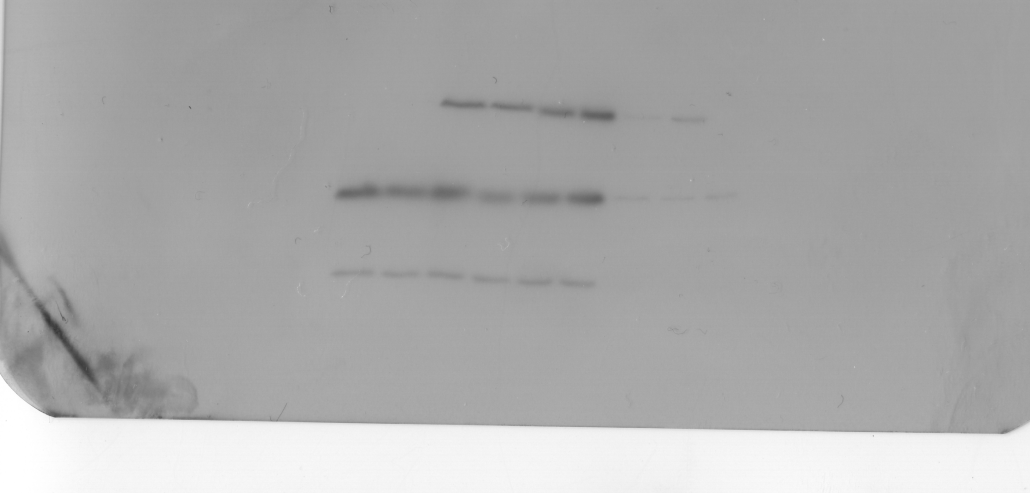

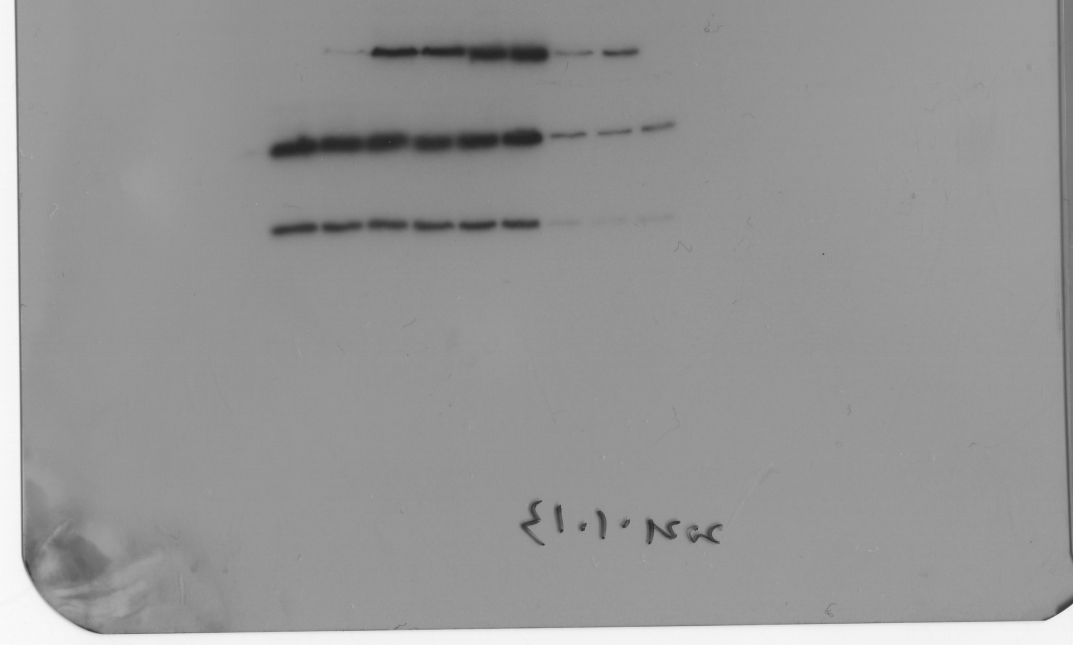


RRP15:


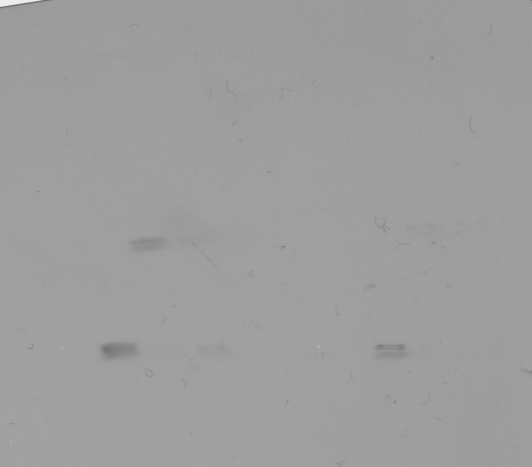

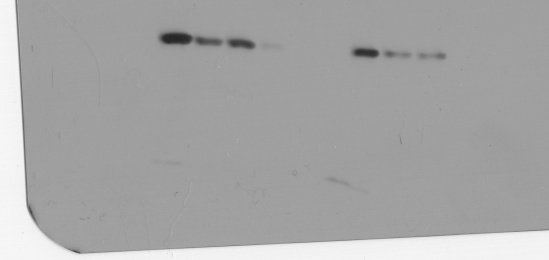


Tubulin:


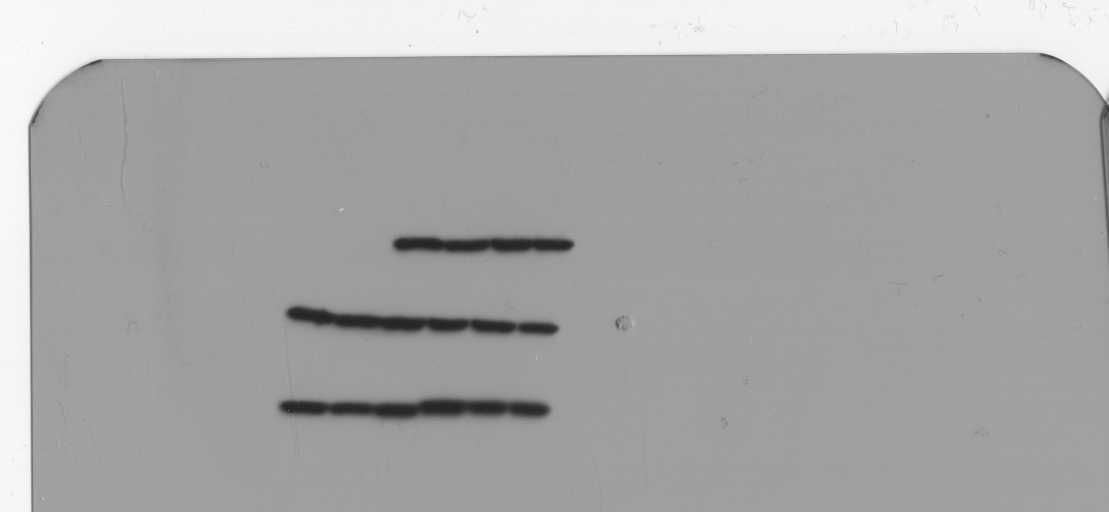


H3:


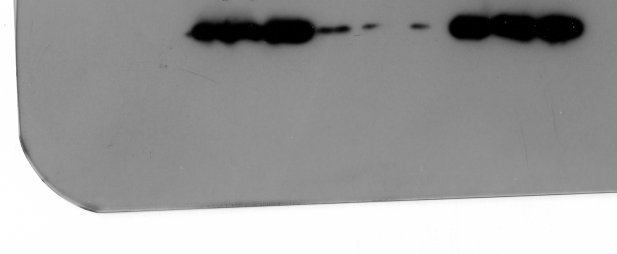


**Figure 7A:**

β-catenin: LZTS2:




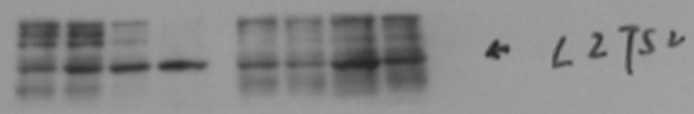


RRP15: GAPDH:


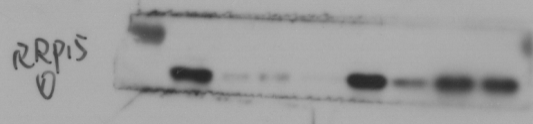

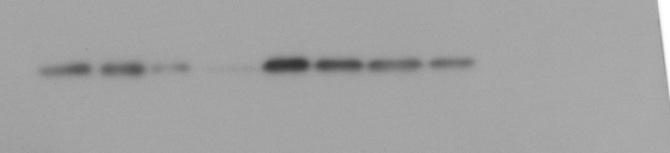


Figure 7D:

β-catenin:


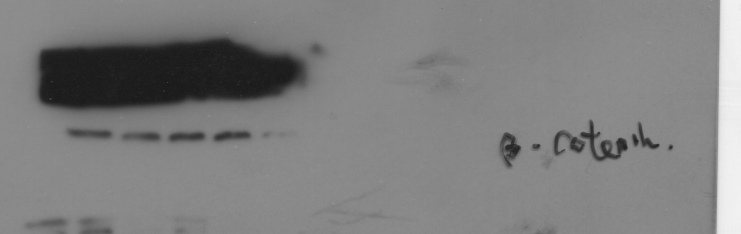


LZTS2:


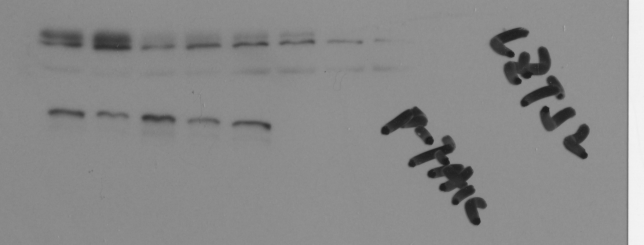


RRP15 and GAPDH:


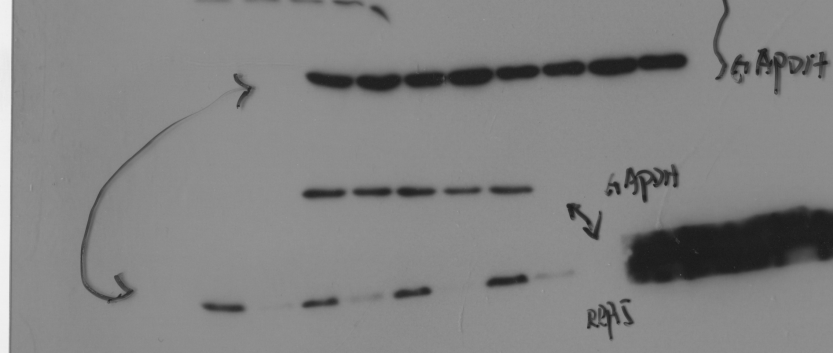


**Figure S4A:**

GAPDH: RRP15:







**Figure S4C:**


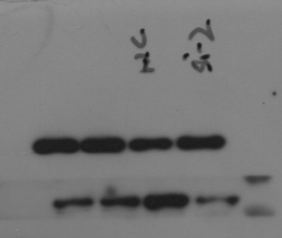


**Figure S4E:**

caspase 9:


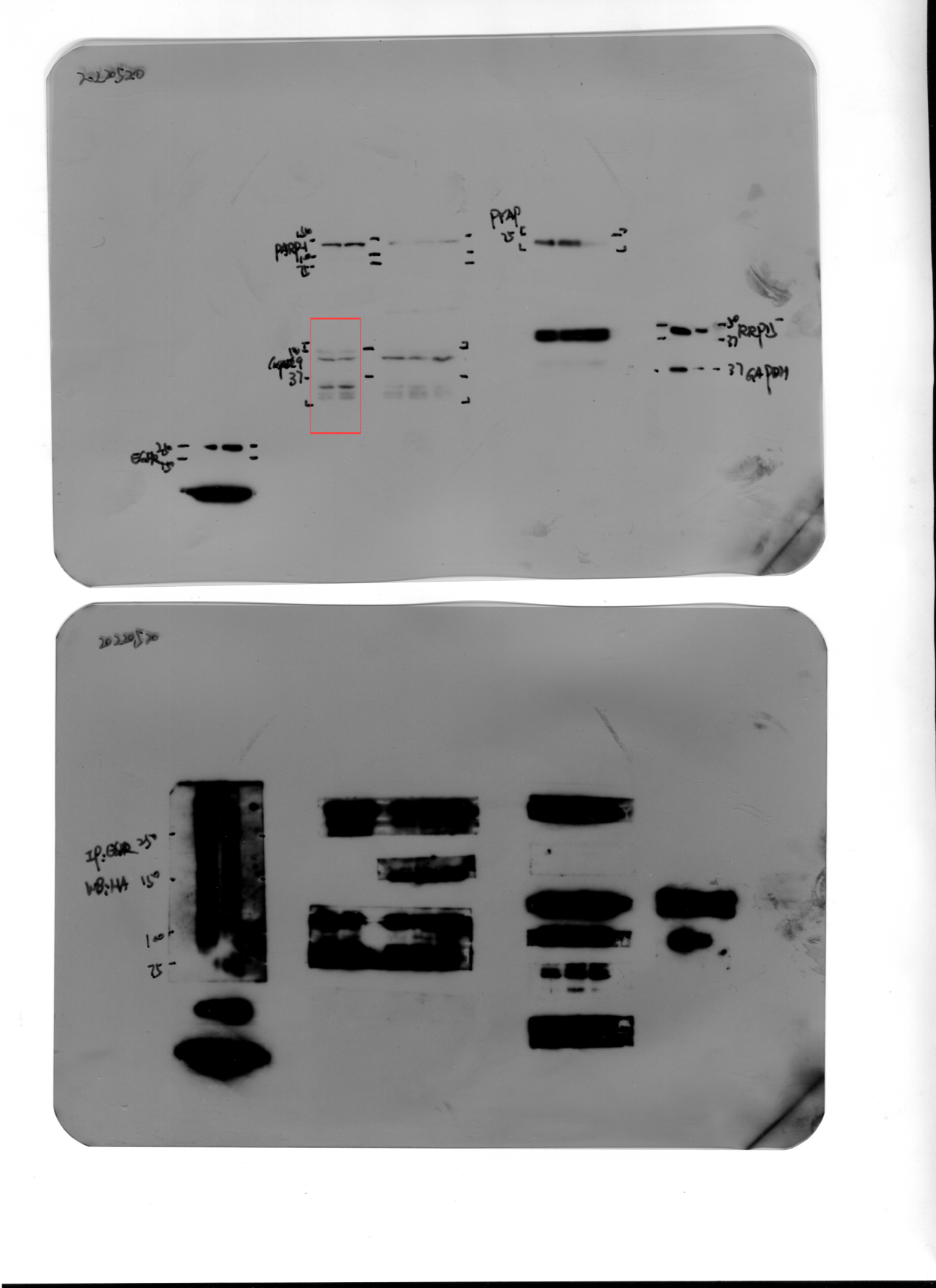


caspase 7: RRP15:


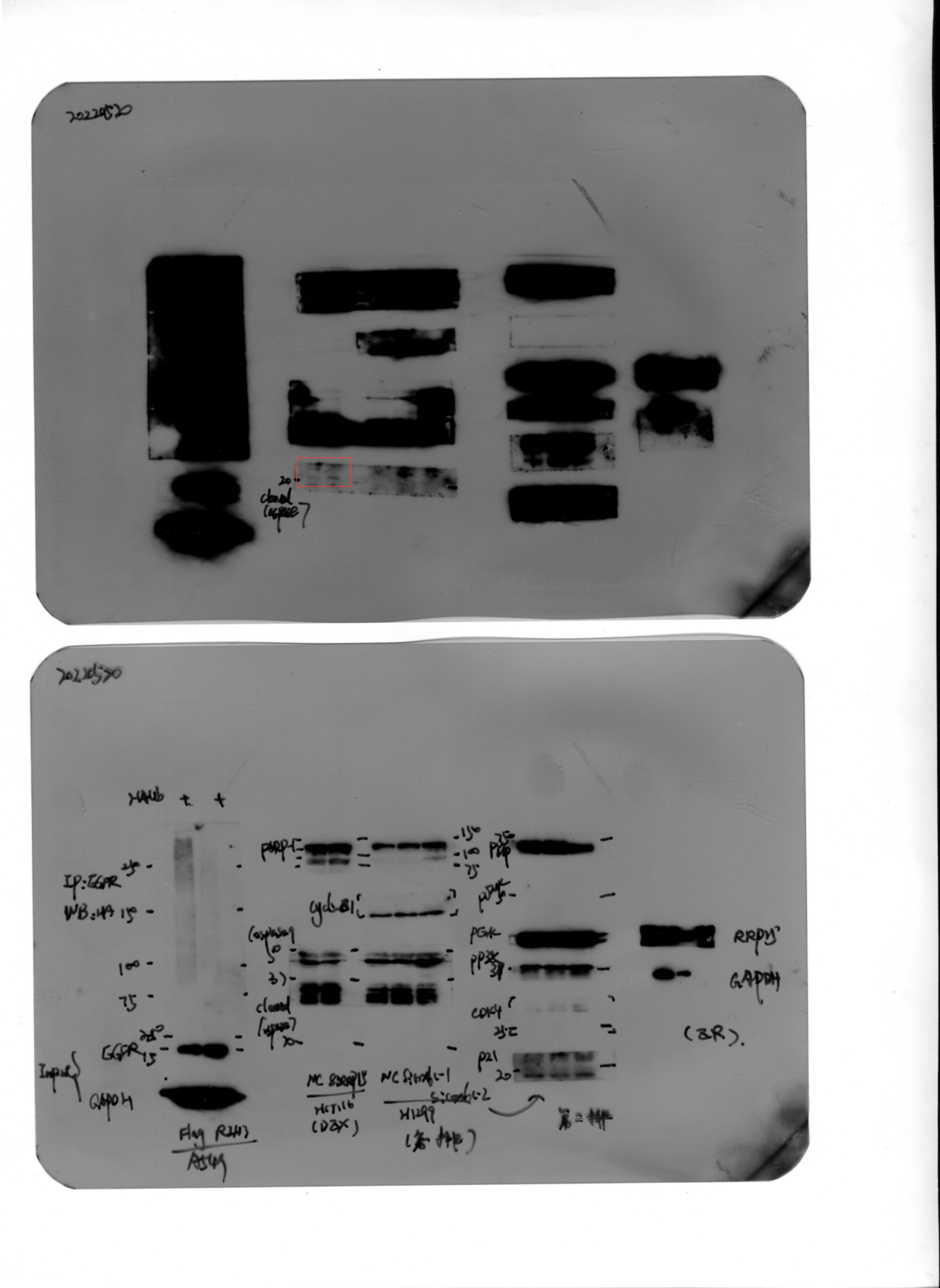

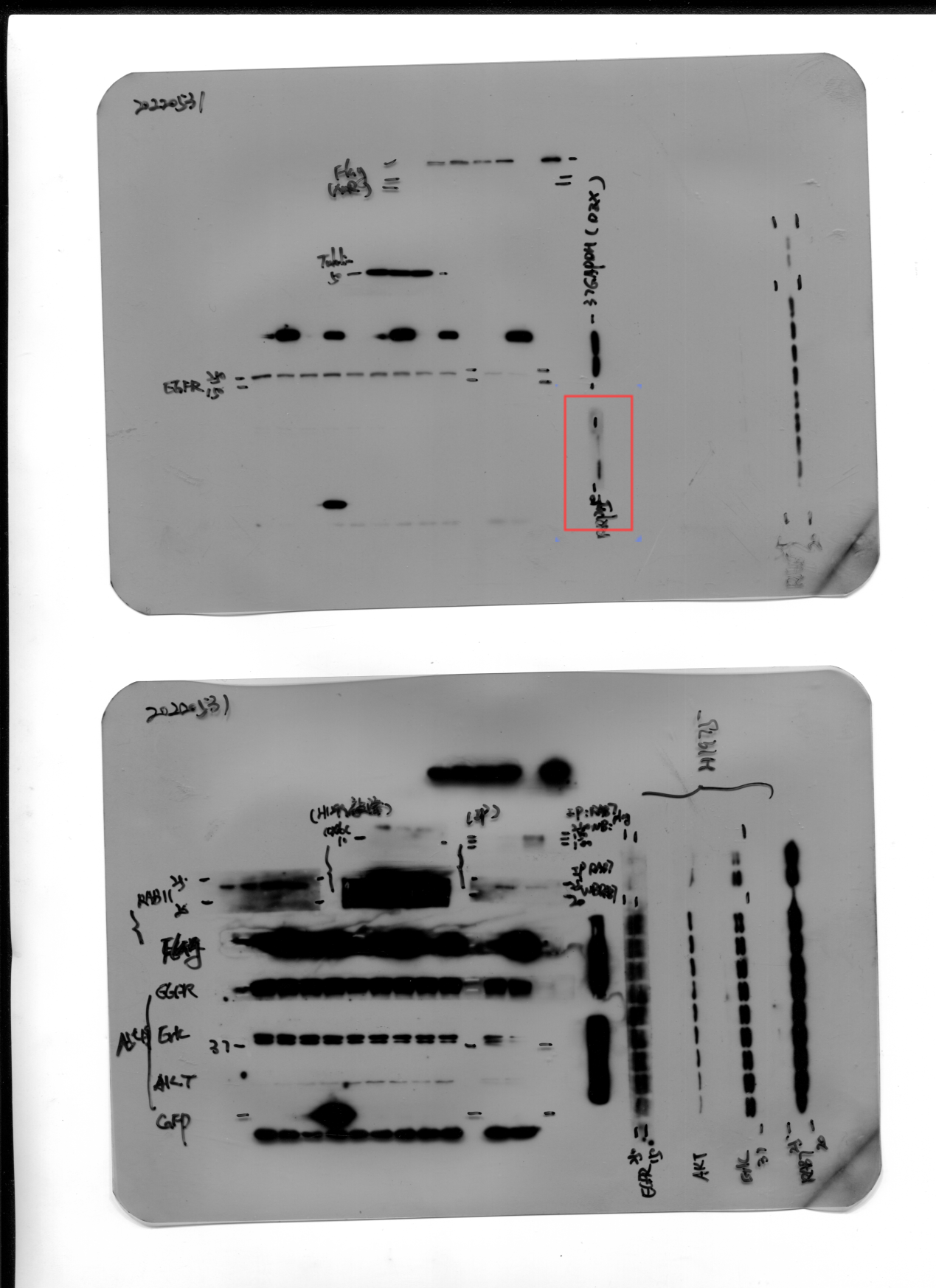


GAPDH:


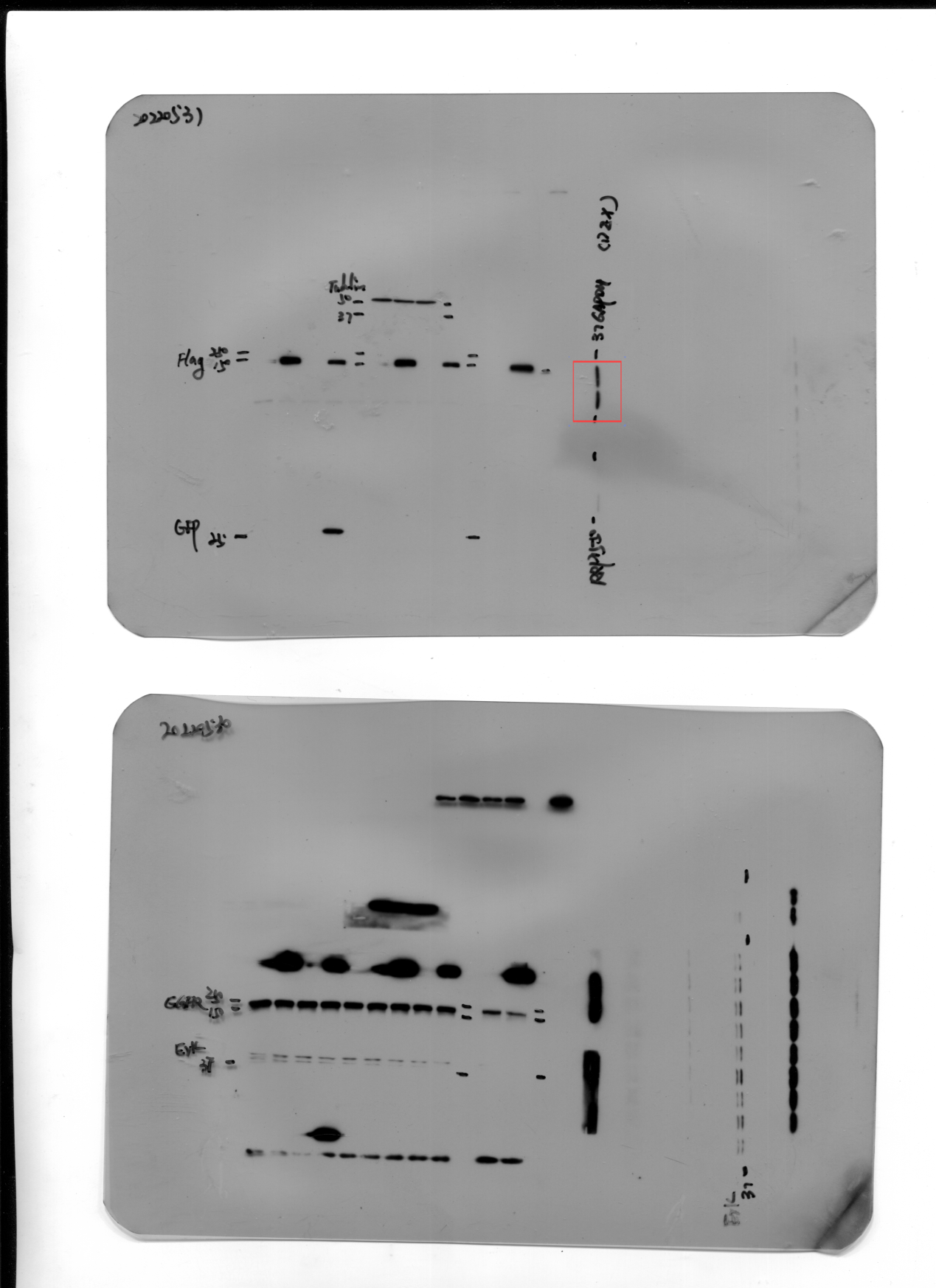


**Figure S4F:**

RRP15: GAPDH:


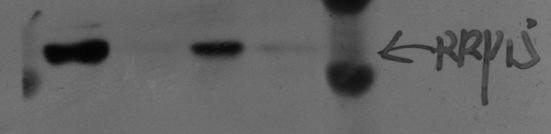

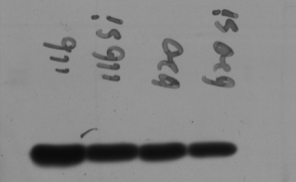


**Figure S4K:**

cyclin E1: cyclin B1:







PCNA: CDK2: RRP15: GAPDH:






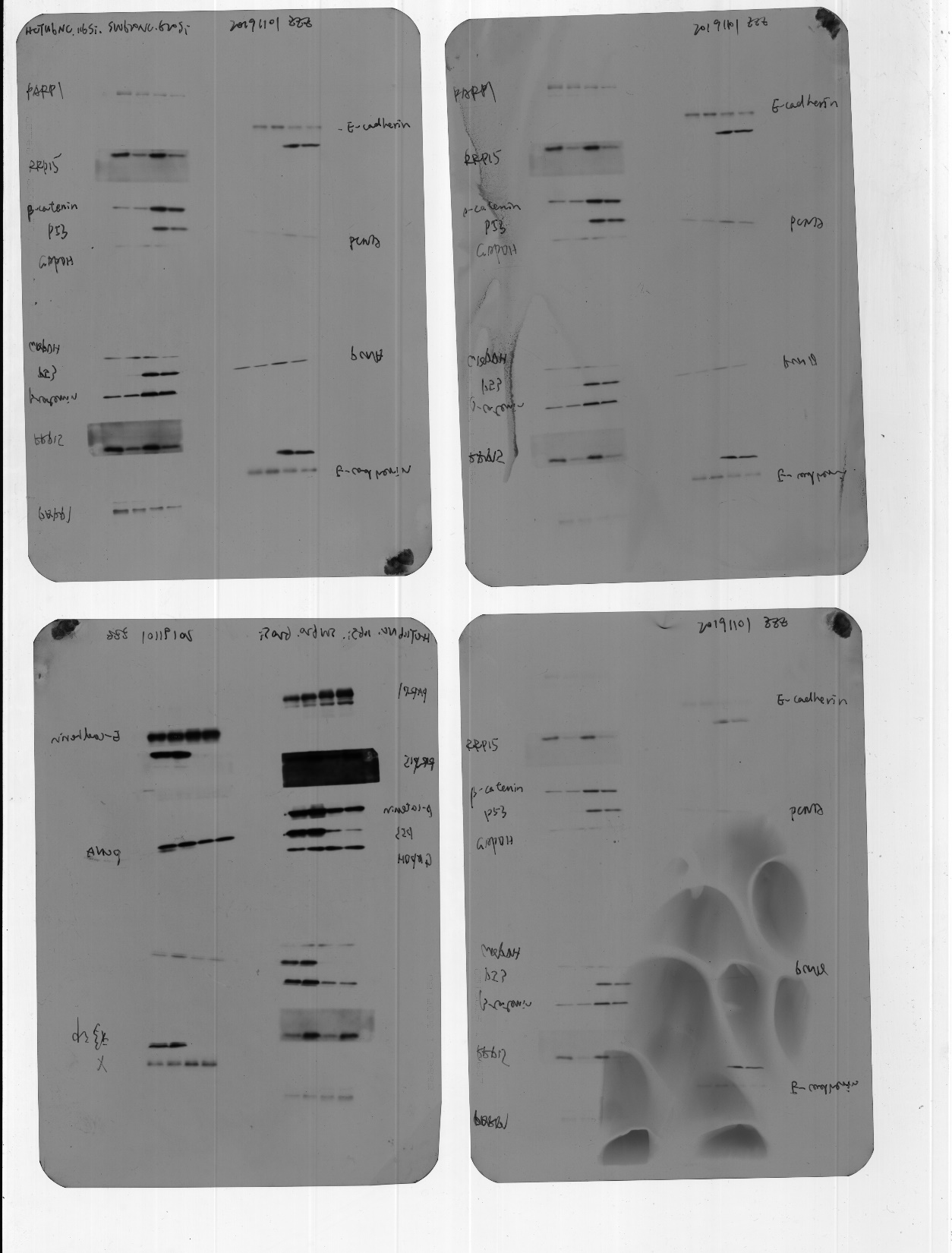

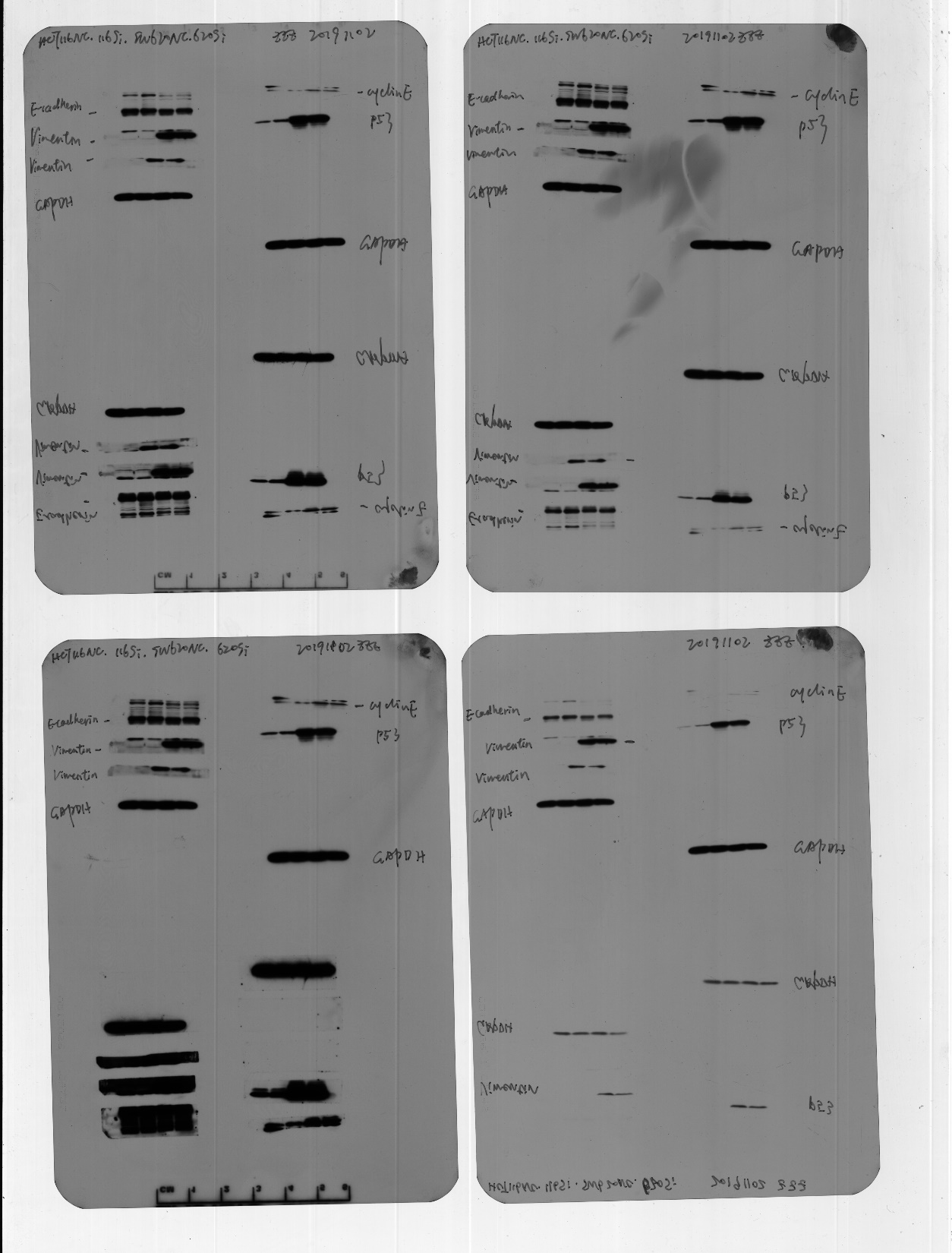


**Figure S4N:**

RRP15 and GAPDH:


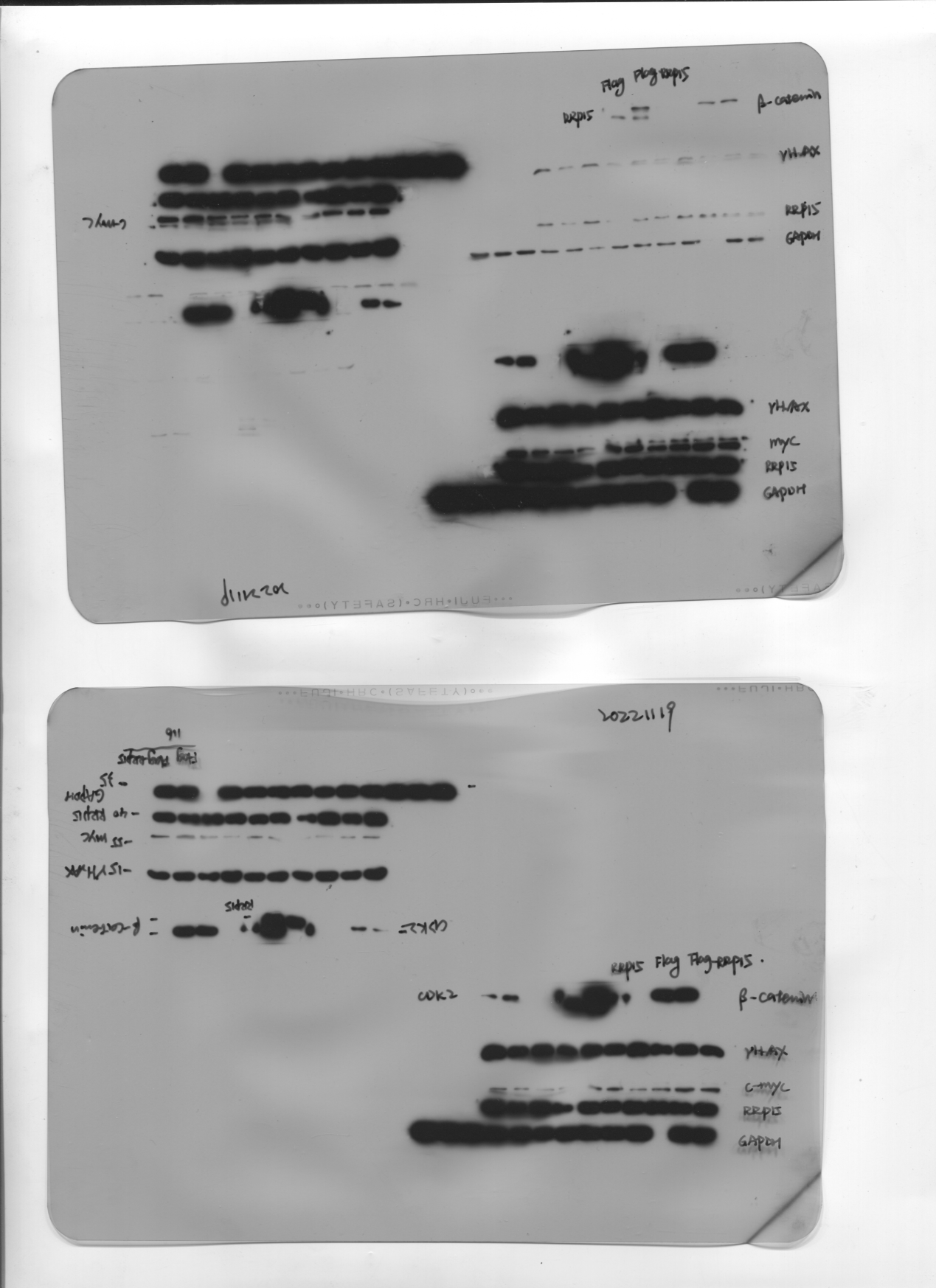


**Figure S5A:**

RRP15: GAPDH:


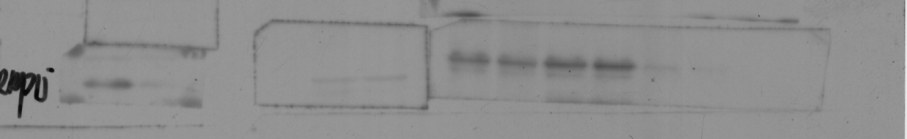

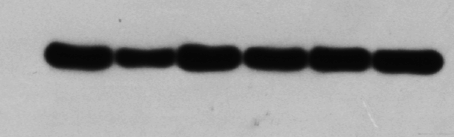


**Figure S6A:**

E-cadherin: Vimentin: N-cadherin:


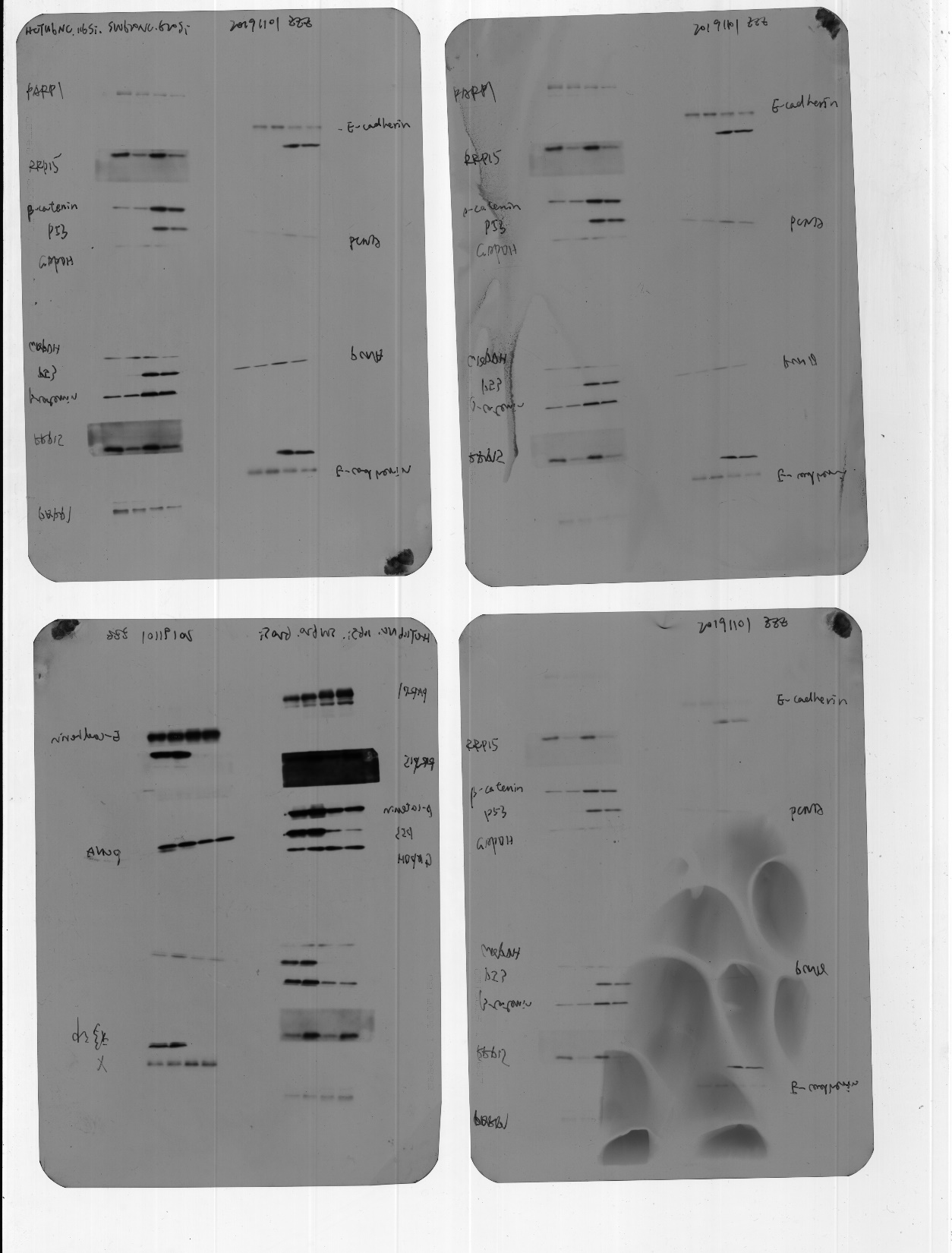



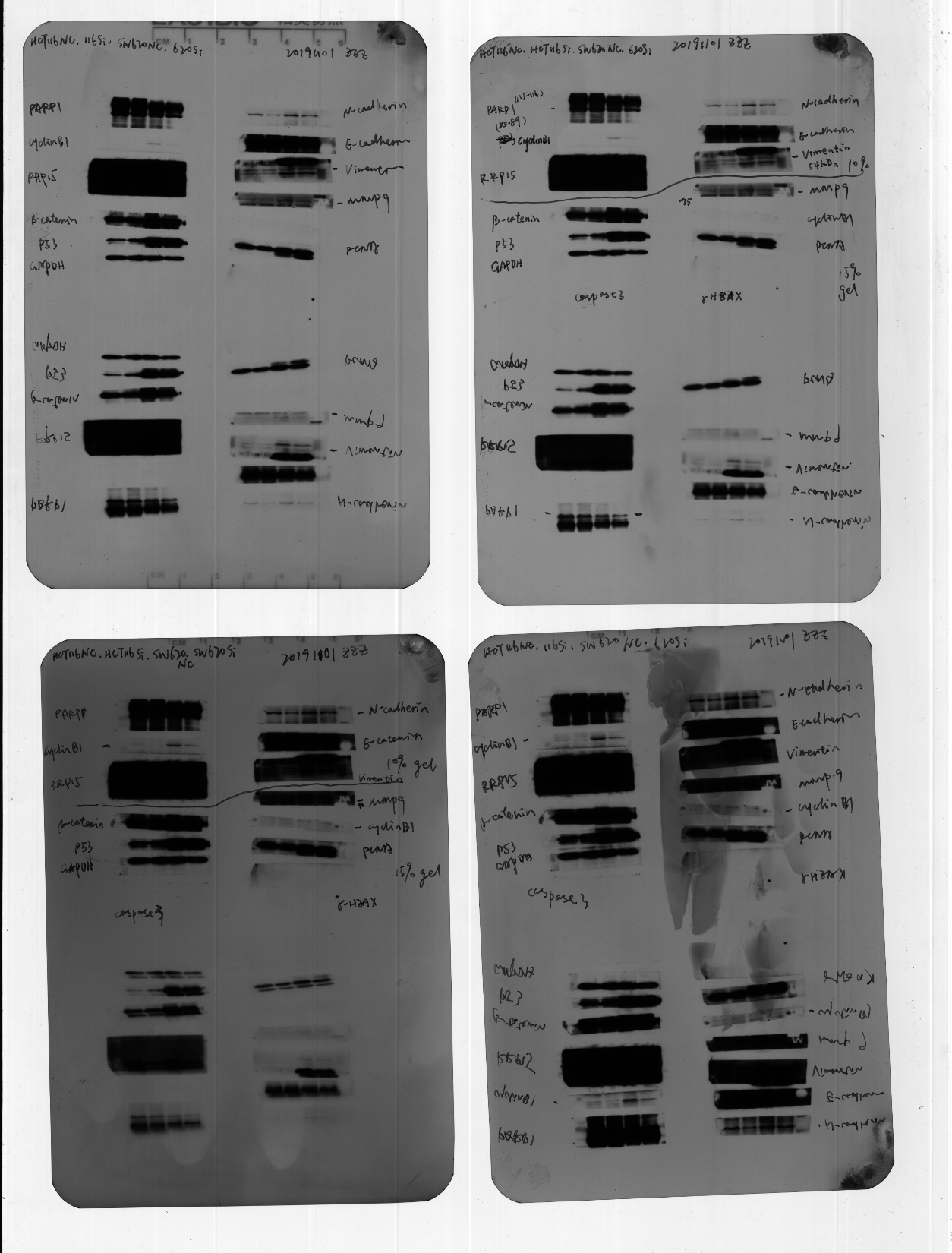


MMP9: RRP15: GAPDH:

**

**


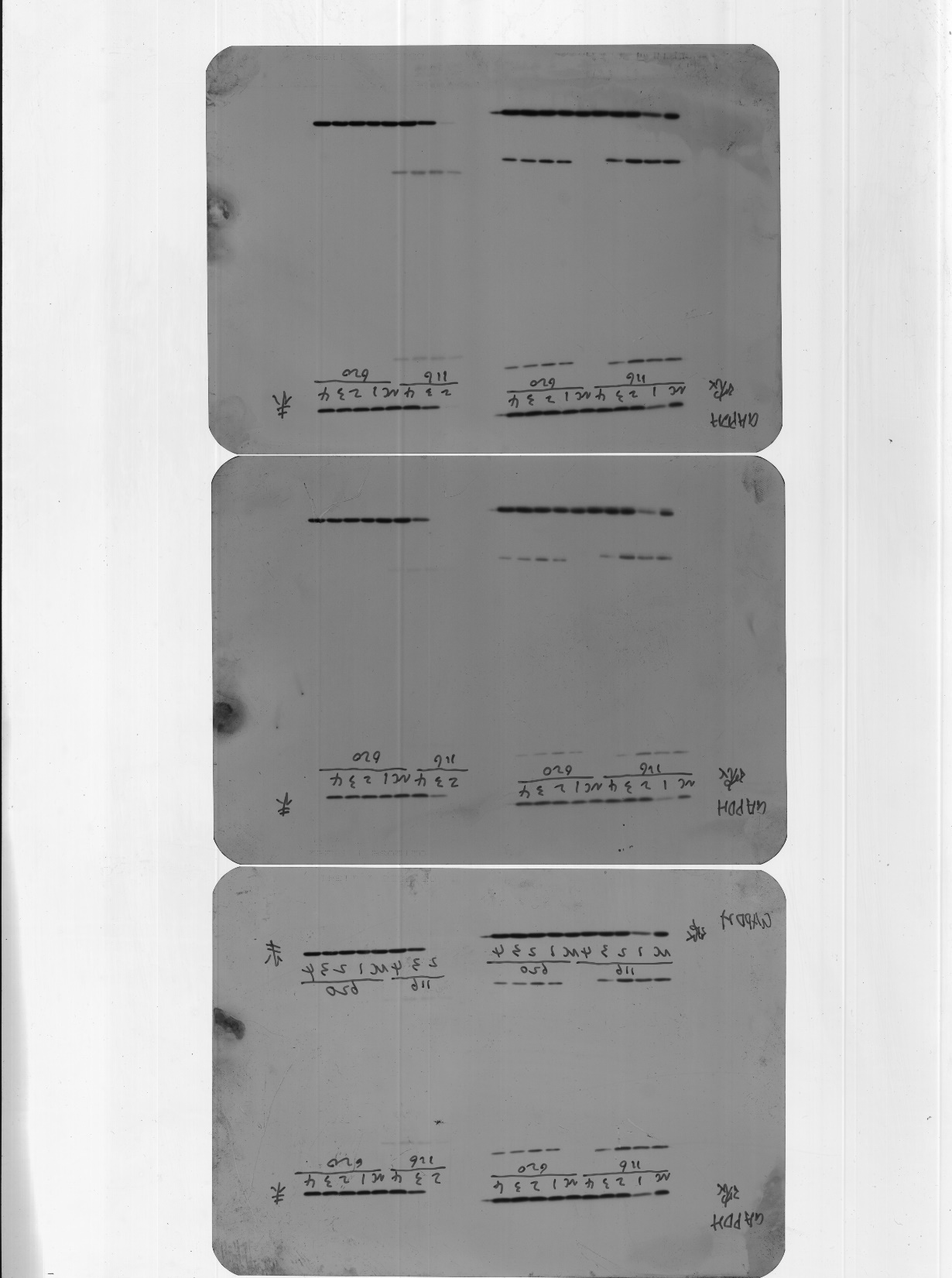


**Figure S6C:**

E-cadherin: MMP9: Vimentin:


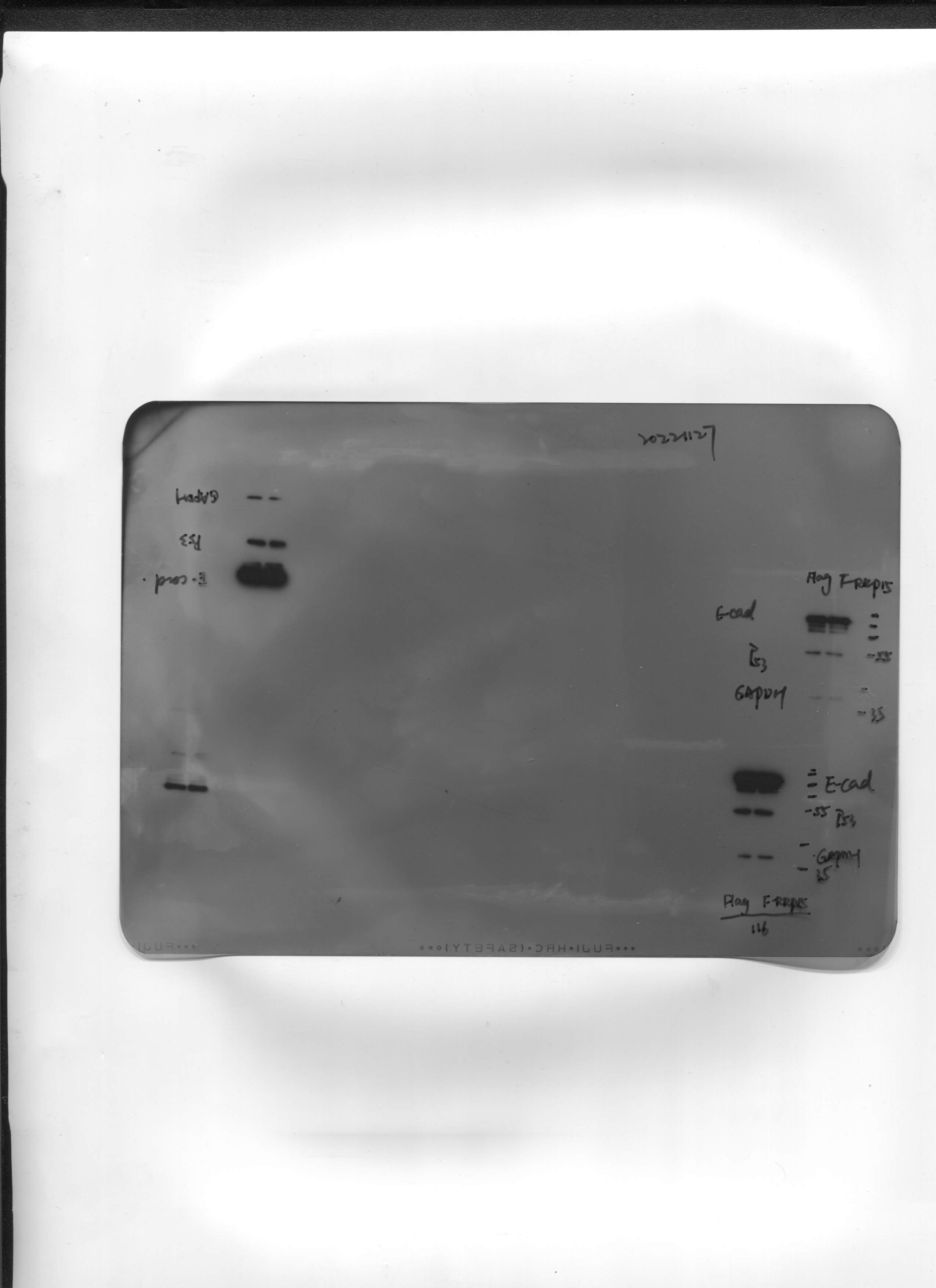

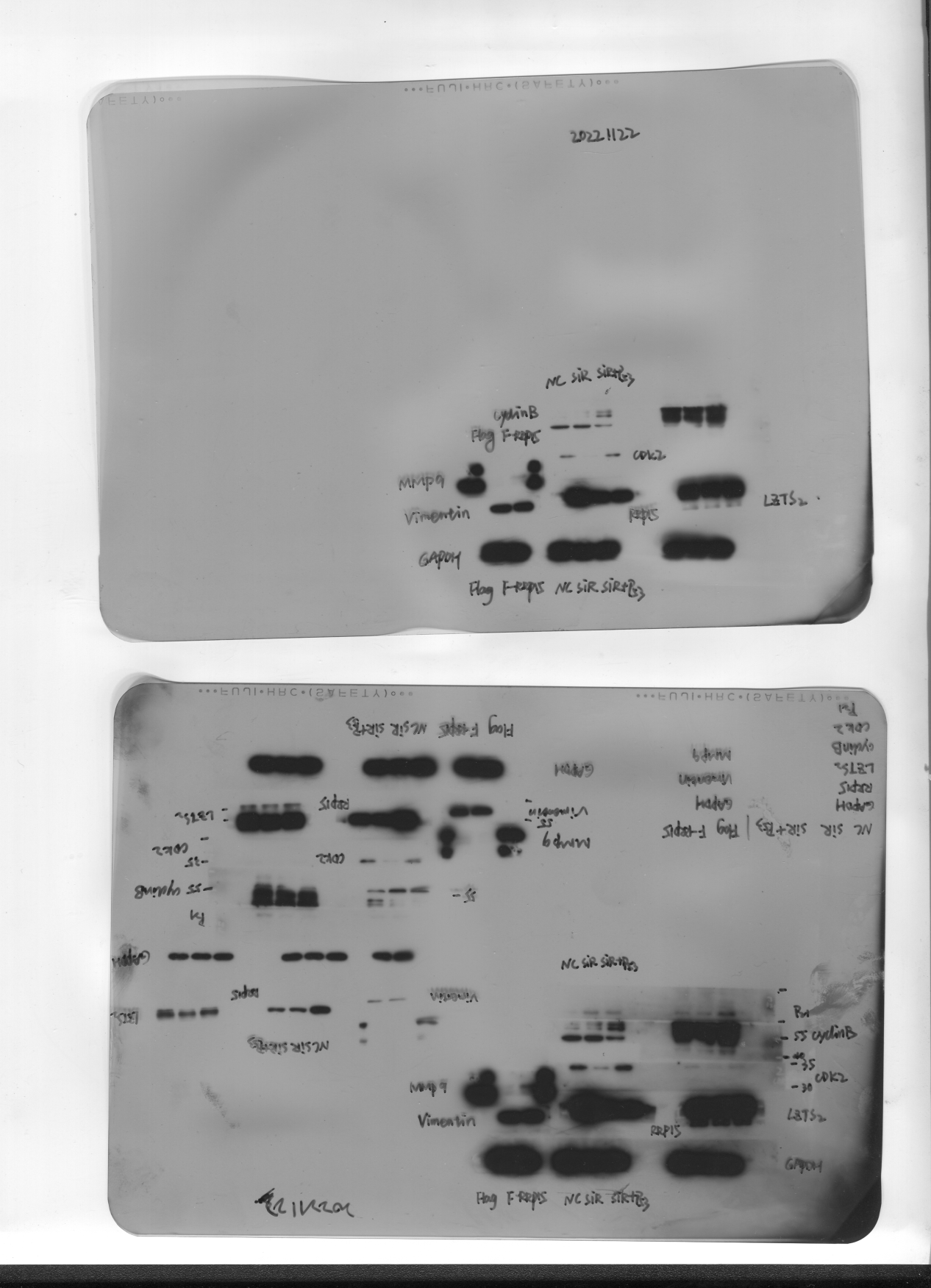

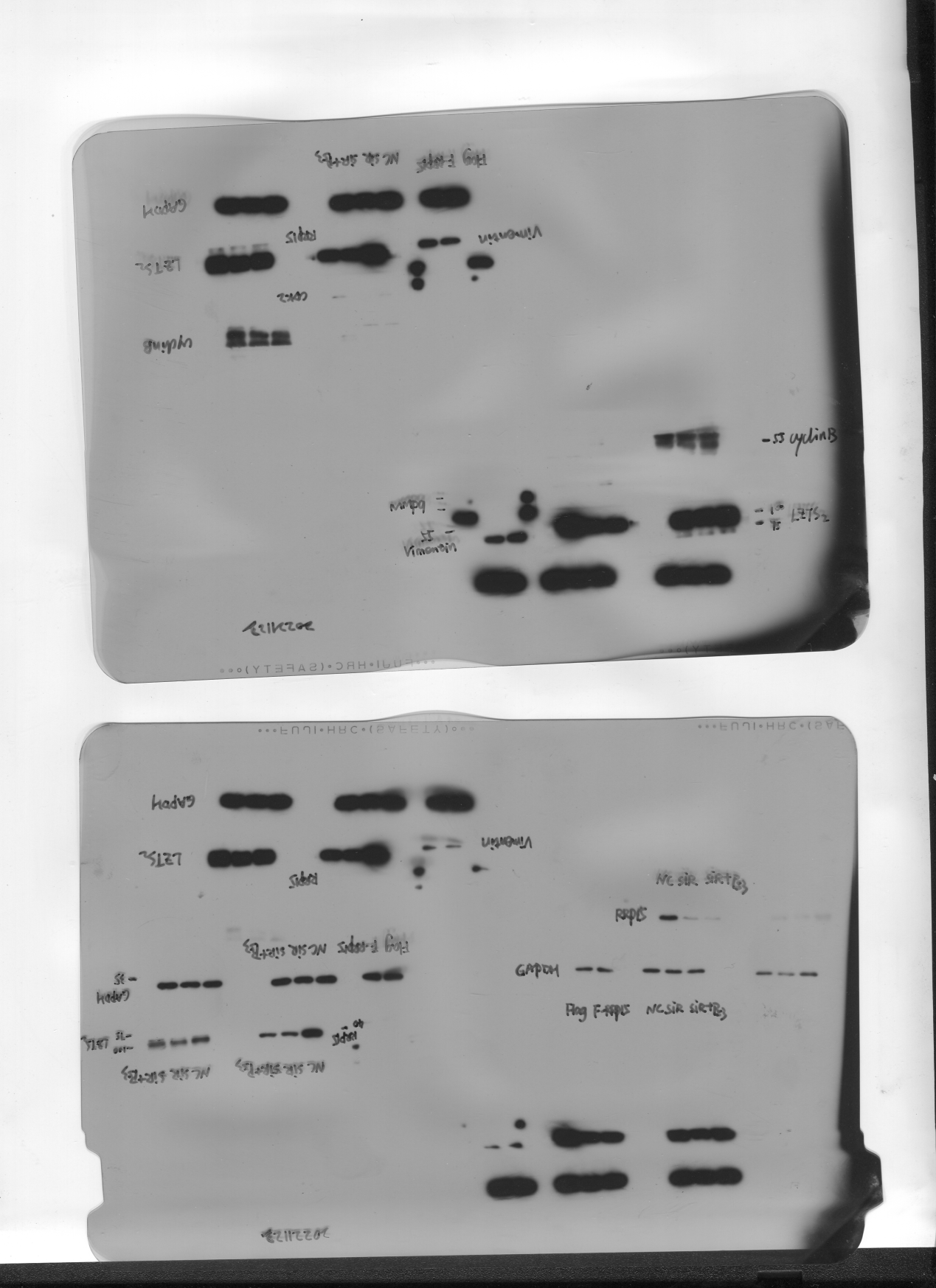


RRP15 and GAPDH:


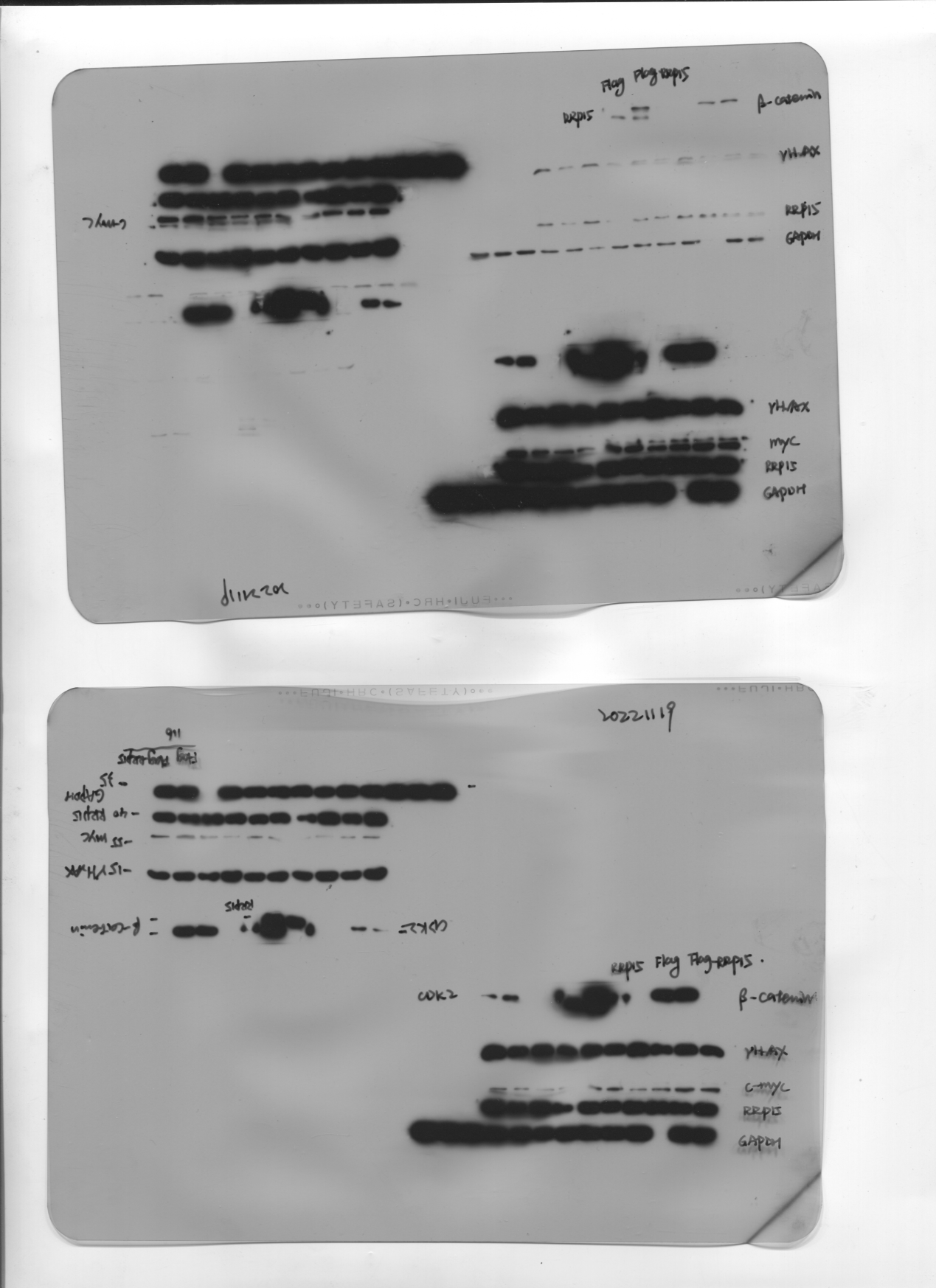


**Figure S8D:**

β-catenin: LZTS2: RRP15 and GAPDH:




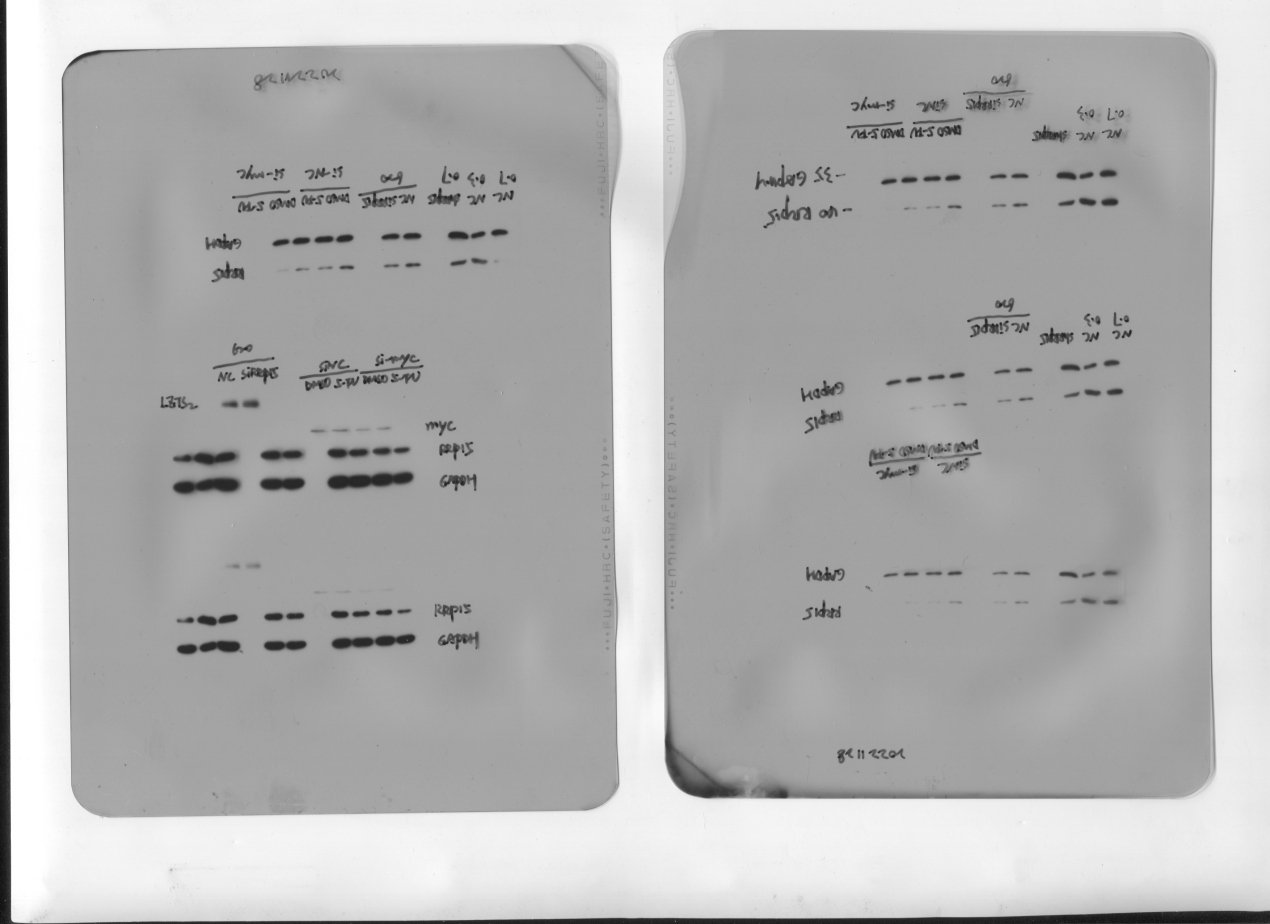




**Figure S8F:**

β-catenin:


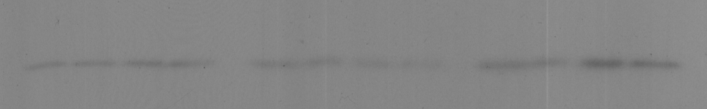


RRP15:


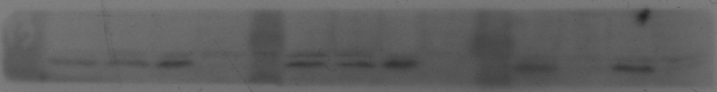


GSK-3β:


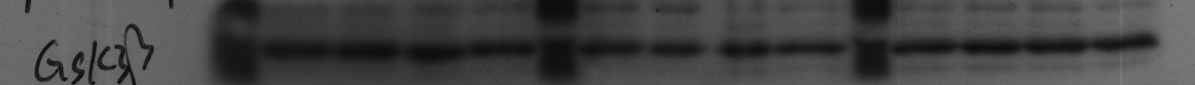


GAPDH:


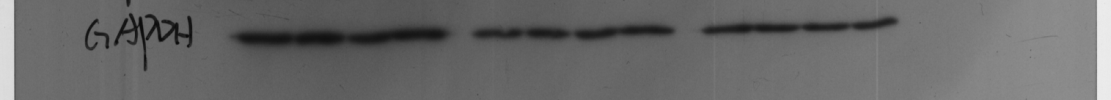


**Figure S8G:**

β-catenin and GAPDH: cyclin D1:







Flag and RRP15:





**Figure S8H:**

β-catenin: Flag:


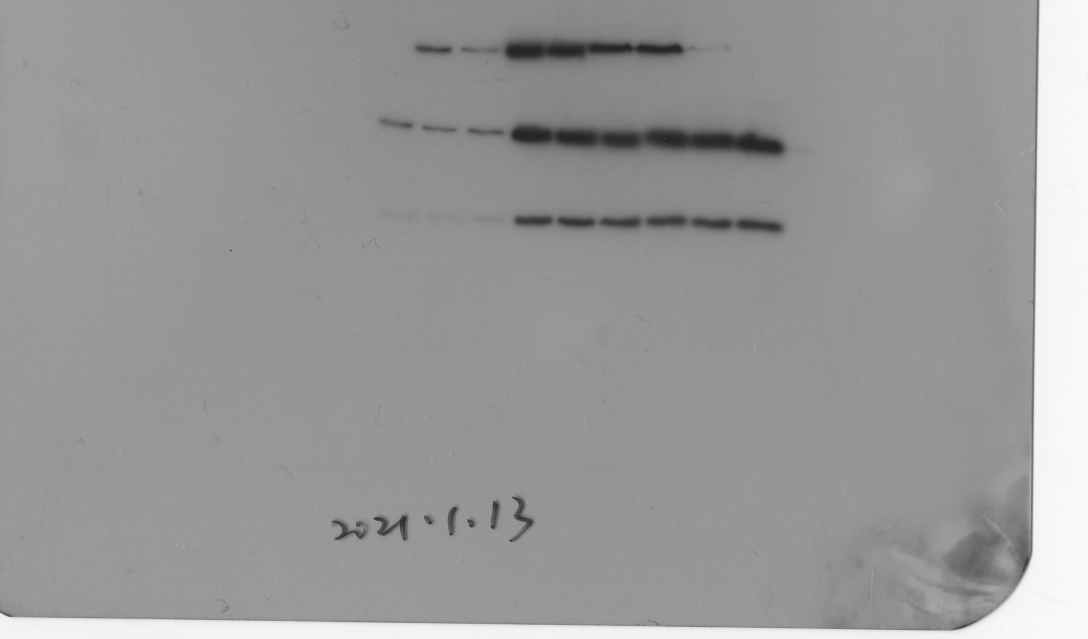

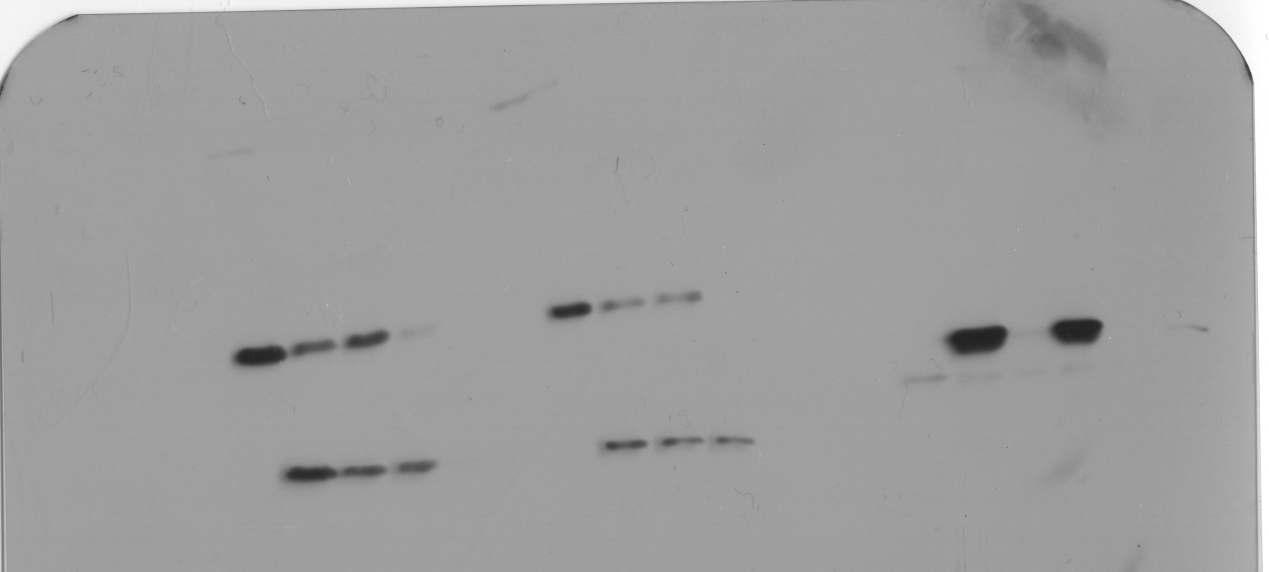


Tubulin: H3:


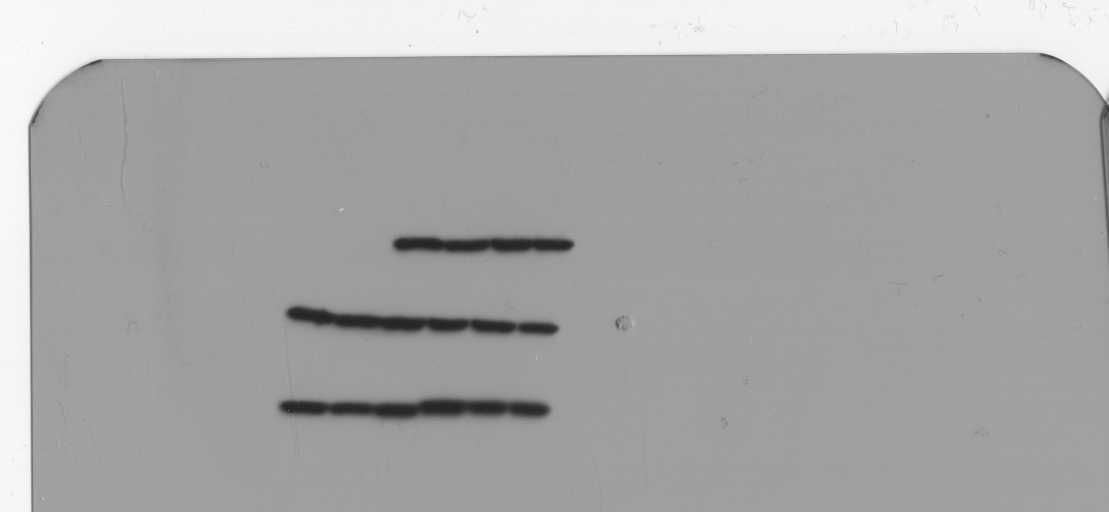

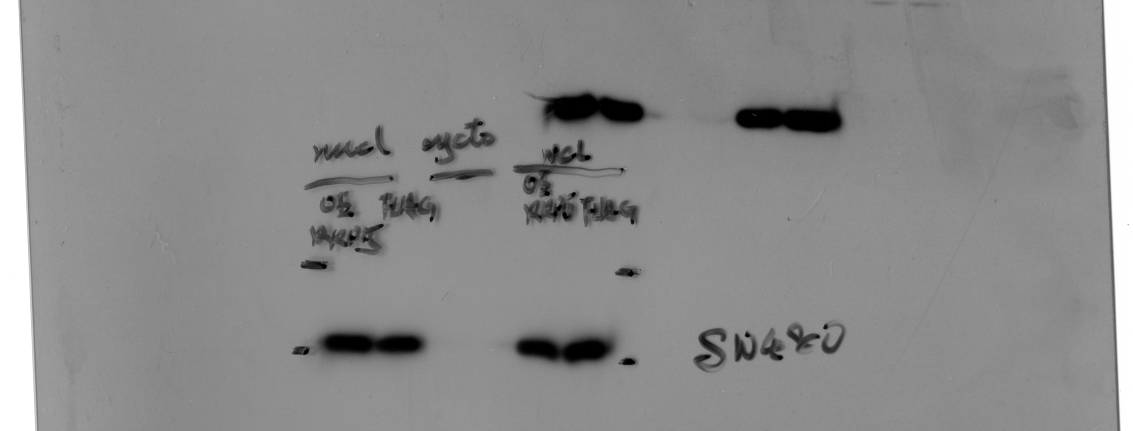


**Figure S9C:**

β-catenin:


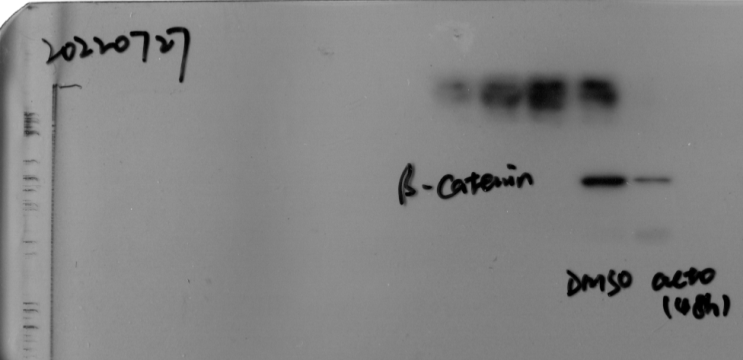


GAPDH:


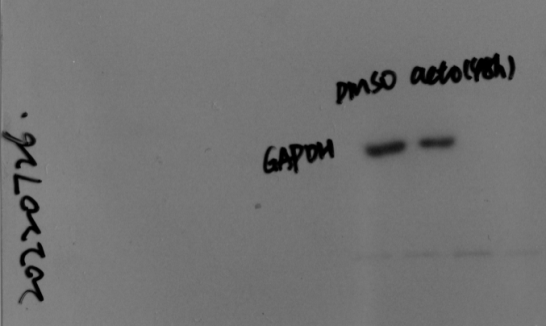


**Figure S12A:**

β-catenin:


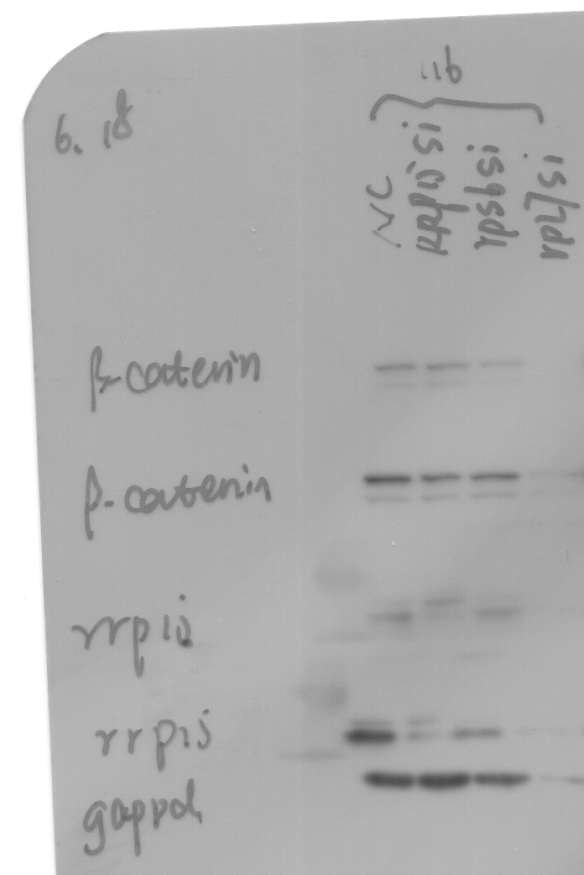


LZTS2 and GSK3β:


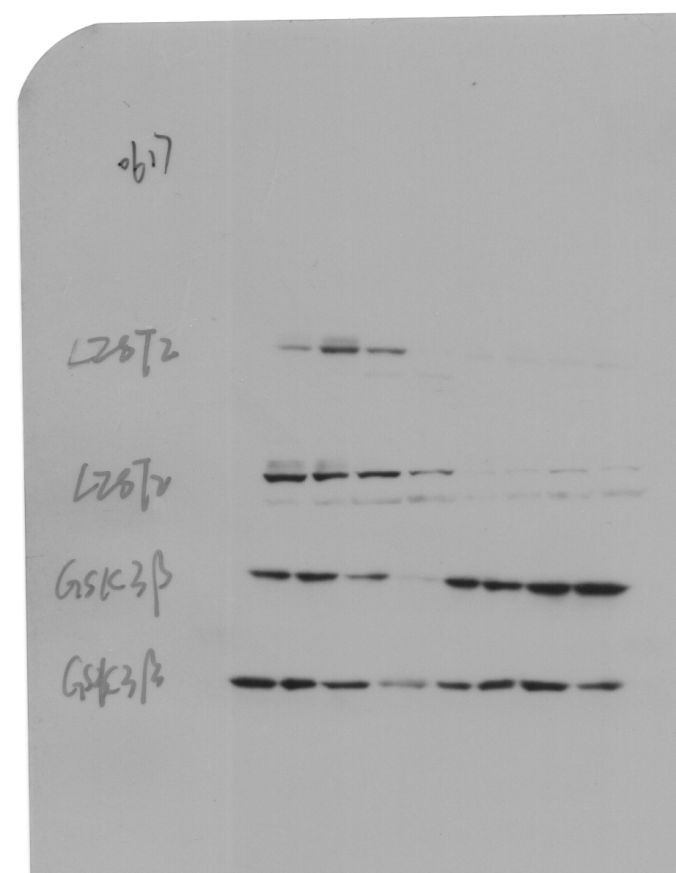


DKK1:


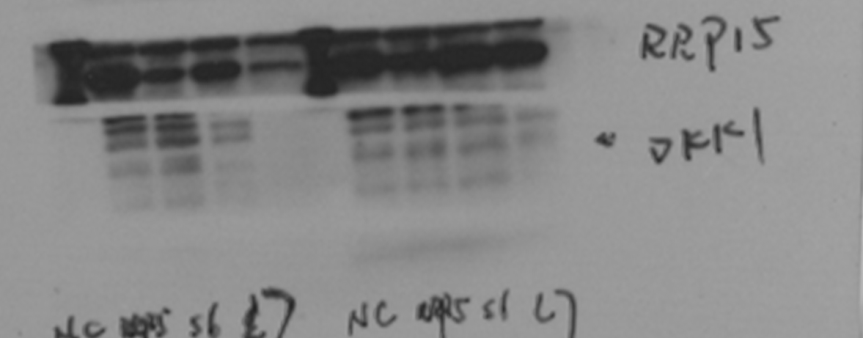


RRP15 and GAPDH:


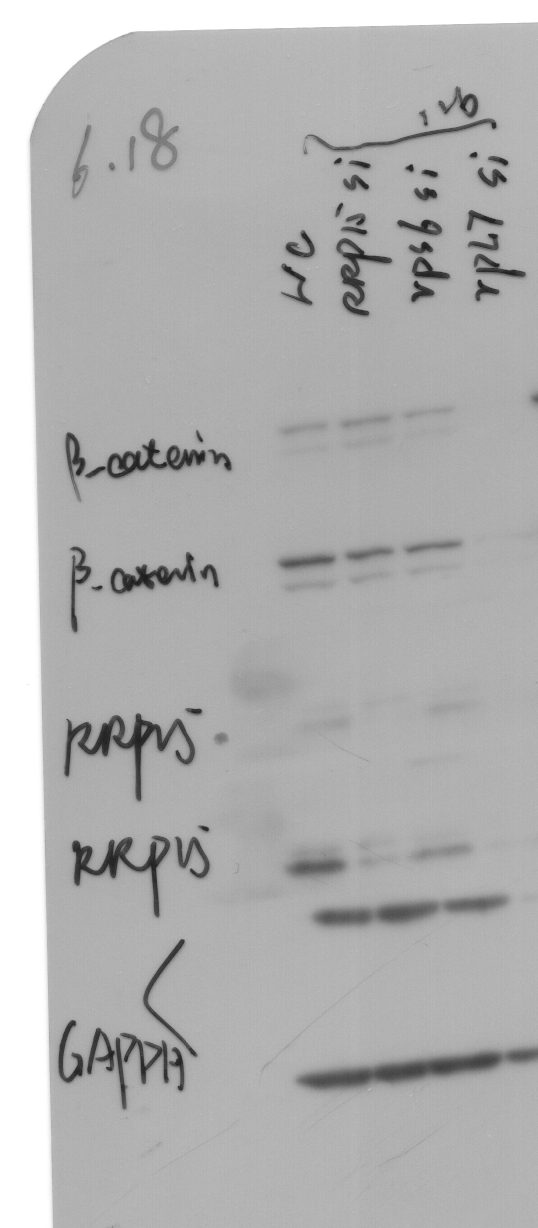


**Figure S13A:**

p21: p53: β-catenin:

**
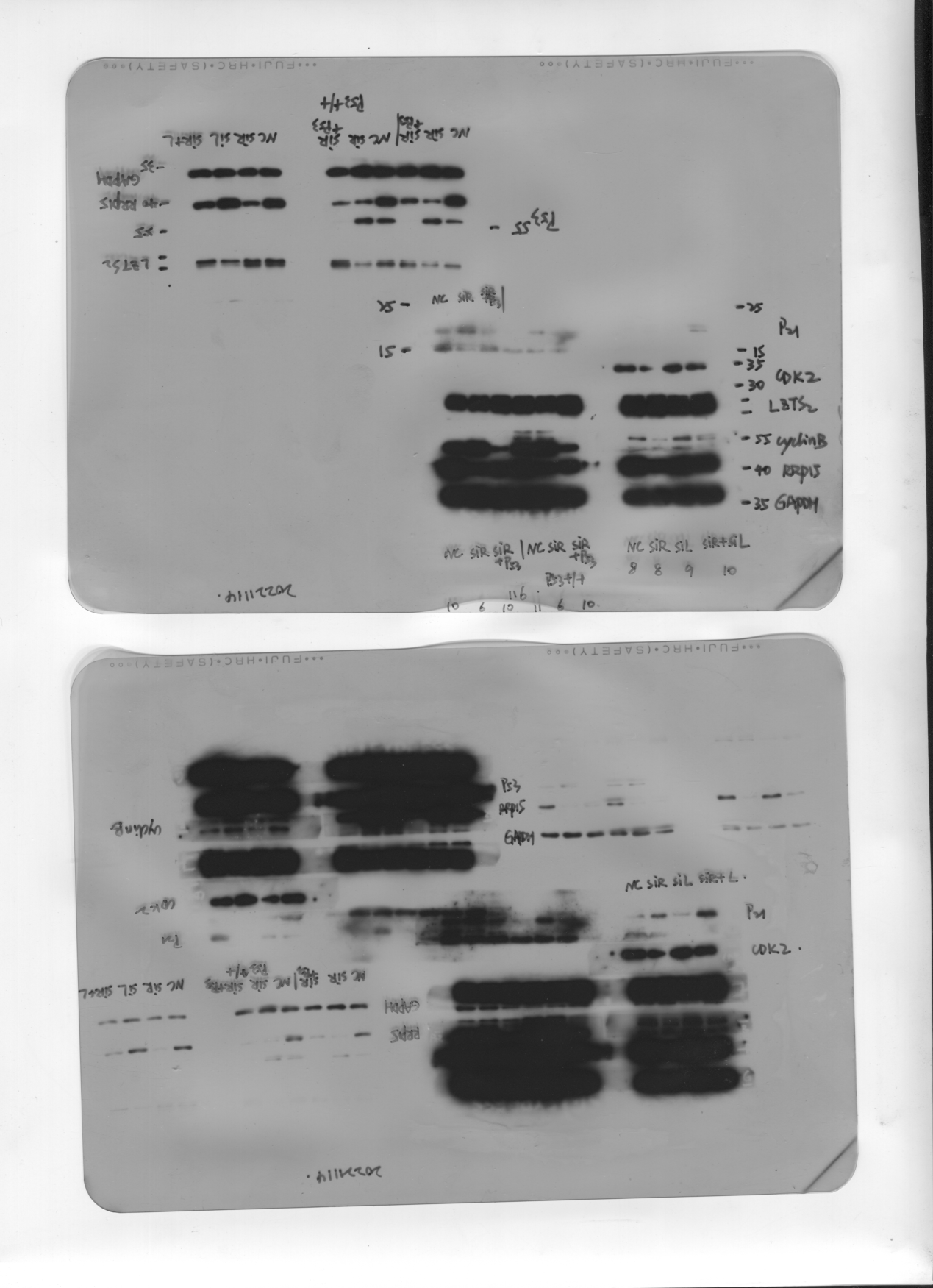

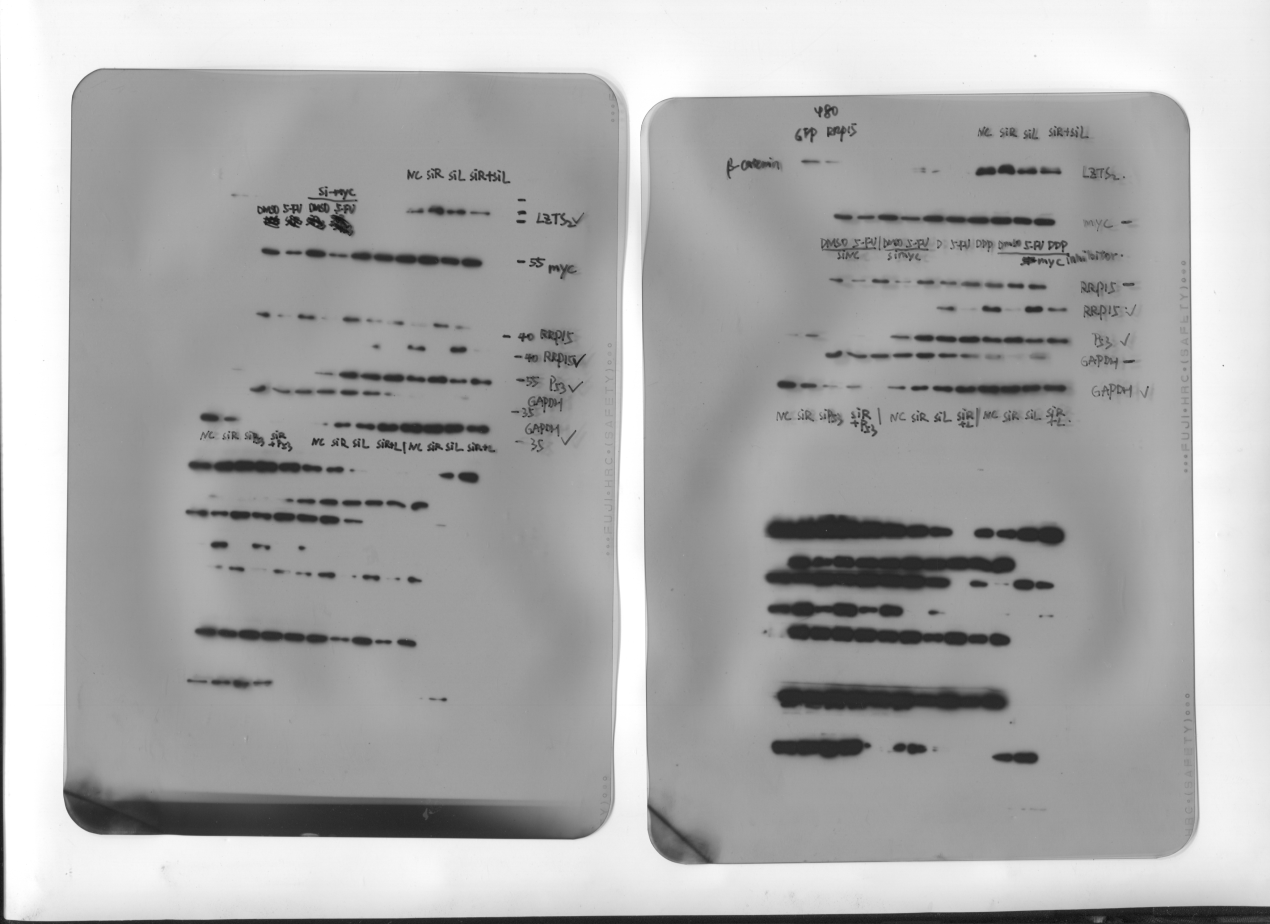

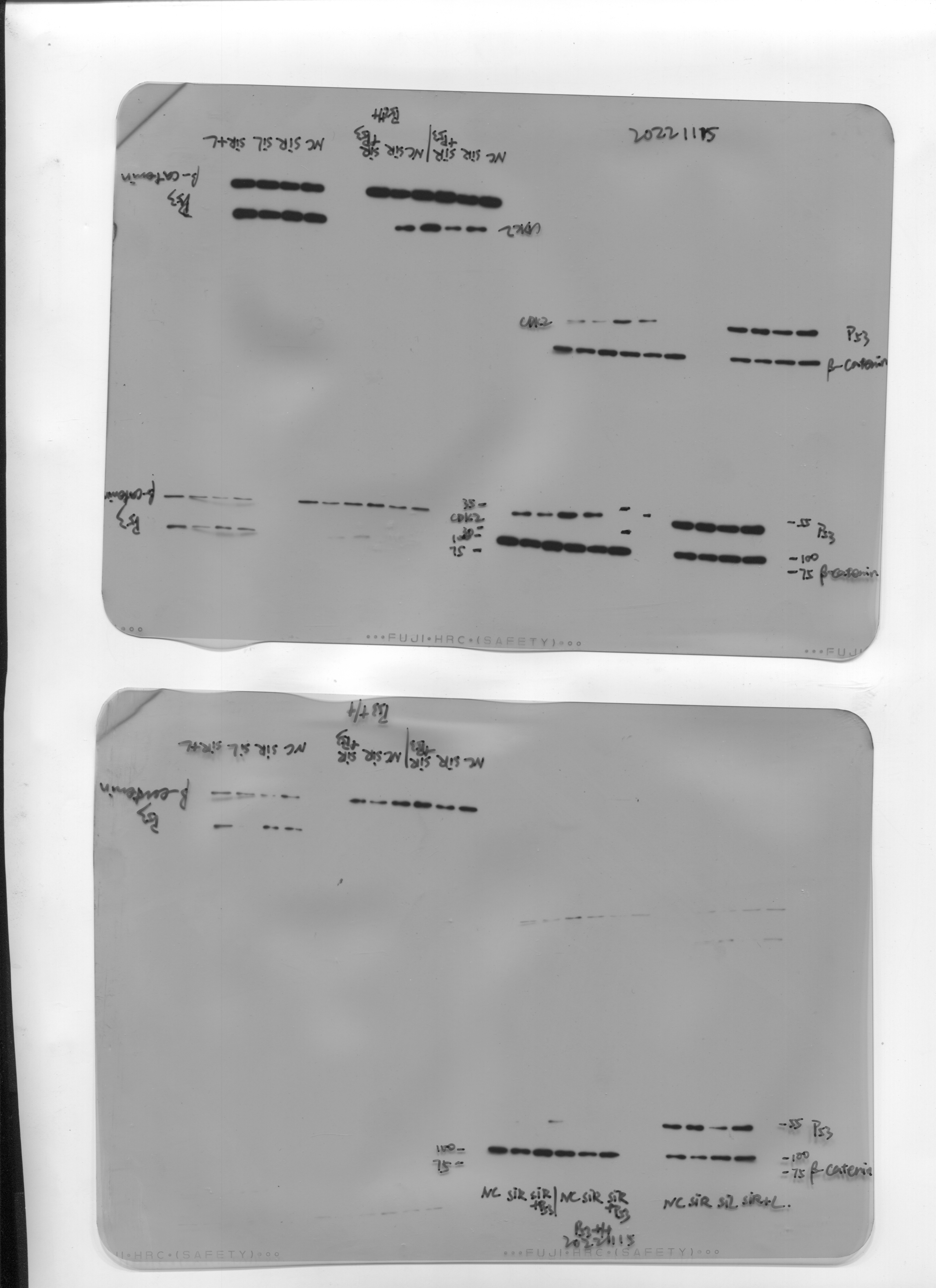
**

LZTS2: RRP15: GAPDH:

**Figure S13B:**

cyclin B1: CDK2:

LZTS2: RRP15: GAPDH:
